# Foot progression angle modifications alter hip joint contact forces and femoral growth plate mechanics in children

**DOI:** 10.3389/fbioe.2026.1799347

**Published:** 2026-04-28

**Authors:** Willi Koller, Elias Wallnöfer, Laura Rathmair, Clara Egner, Kevin Kitir, Hans Kainz

**Affiliations:** 1 Department of Biomechanics, Kinesiology and Computer Science in Sport, Centre for Sport Science and University Sports, University of Vienna, Vienna, Austria; 2 Neuromechanics Research Group, Centre for Sport Science and University Sports, University of Vienna, Vienna, Austria; 3 Vienna Doctoral School of Pharmaceutical, Nutritional and Sport Sciences, University of Vienna, Vienna, Austria

**Keywords:** finite element analysis, foot progression angle, gait modification, growth plate, joint contact forces, mechanobiology, MRI-based EMG-informed MSK simulations, stress distribution

## Abstract

**Introduction:**

Bony deformities of the lower limbs are prevalent in both children and adults and are associated with pain, gait impairments, and increased risk of osteoarthritis. While severe torsional deformities are commonly treated surgically, bone growth is regulated by mechanical stimuli, suggesting potential for non-invasive interventions. In children, longitudinal bone growth occurs at epiphyseal growth plates through endochondral ossification, where chondrocyte activity is modulated by biochemical and mechanical cues. Mechanobiological theories indicate that compressive and shear stresses influence local growth rates. Gait modifications are practical, coachable strategies to alter lower-limb loading in vivo, yet direct evidence that such modifications acutely affect growth-related mechanical stimuli within paediatric growth plates is lacking. Most prior studies report joint moments rather than subject-specific joint contact force (JCF) or micro-scale stresses. The present study addresses this gap by integrating MRI-based, electromyography (EMG)-informed musculoskeletal (MSK) modelling with subject-specific finite element (FE) analyses to quantify how gait modifications alter growth-related stresses in femoral growth plates.

**Methods:**

Three typically developing children walked on an instrumented treadmill under preferred gait and bilateral in-toeing and out-toeing conditions. MRI-based MSK models combined with EMG-informed optimization were used to estimate muscle forces and hip, knee, and patellofemoral JCFs, which were applied as loading conditions in FE models of the proximal and distal femoral growth plates. Hydrostatic compressive and octahedral shear stresses were quantified as mechanobiologically relevant growth stimuli.

**Results:**

In-toeing increased hip JCF magnitude and shifted its orientation anteriorly across all participants, accompanied by increases in hydrostatic compression and more heterogeneous shear stress distributions in the proximal femoral growth plate. Changes at the knee, patellofemoral joint, and distal growth plate were smaller and more variable between participants, likely reflecting anatomical and neuromuscular differences. Overall gait kinetics and estimated forces aligned with previous reports.

**Discussion:**

These findings provide proof-of-concept that modifiable gait patterns can acutely alter mechanobiologically relevant stresses in paediatric femoral growth plates. While responses were subject-specific, the results support the feasibility of personalised gait retraining as a low-risk, non-invasive strategy to influence growth-related mechanical loading. Larger longitudinal studies are required to determine whether such acute changes can steer bone development during maturation.

## 1 Introduction

Bony deformities of the lower limbs are common in adults as well as children and are linked to several clinical problems, e.g. increased risk for osteoarthritis, gait impairments or pain (Alexander et al., 2019; Bobroff et al., 1999; Bruderer-Hofstetter et al., 2015). Torsional deformities, i.e. increased or decreased femoral anteversion angle or tibial torsion are typically corrected with severe de-rotation osteotomies. However, it is known that mechanical stimuli guide how bones develop (Carter and Wong, 1988; Frost, 2001). Other clinical interventions, i.e. temporary hemiepiphysiodesis, make use of these mechanobiological responses to inhibit growth at one side of the bone to correct frontal knee alignment over time (Hucke et al., 2023; Stevens, 2007).

Longitudinal bone growth in children proceeds via endochondral ossification at the epiphyseal growth plates, where chondrocyte proliferation, hypertrophy, and matrix mineralisation are tightly regulated by biochemical and mechanical cues (Kronenberg, 2003; Villemure and Stokes, 2009). Mechanobiological frameworks indicate that increased compression tends to inhibit growth, whereas reduced compression or shear environments can promote growth. The type of stress (hydrostatic vs. shear) is a key determinant of chondrocyte behaviour and local growth rate (Carter and Wong, 1988; Frost, 2001). In the lower limb, both the magnitude and orientation of joint contact forces (JCFs) as well as muscle forces determine stress fields within the femoral epiphyses, linking whole-body loading to local growth stimuli (Kainz et al., 2021a; Koller et al., 2024; Yadav et al., 2017; 2021). In addition to mechanical loads, other factors such as genetics and nutrition affect bone growth (Stevens et al., 1999). The mechanical loads, however, guide the direction of growth and are therefore crucial for the shape of bones (Frost, 2001; Stevens et al., 1999; Wolff, 1892). The present study targets these local mechanical stimuli (hydrostatic pressure and shear) and makes no inference about genetics or systemic endocrine pathways.

Gait modifications are practical levers to modify lower-limb loading *in vivo* (Diamond et al., 2022; Simic et al., 2011; Uhlrich et al., 2022). However, direct evidence that clinically actionable gait modifications acutely change growth-related stimuli within paediatric growth plates is not yet available. Most gait-modification research reports joint moments or loading rather than subject-specific JCF orientation and micro-level stresses. Nevertheless, studies on adults show that altering gait patterns can modify knee loading, supporting the plausibility of steering JCF orientation (Diamond et al., 2022; Uhlrich et al., 2025). In- and out-toeing are practical, coachable gait modifications that adjust the foot progression angle (FPA) and can be applied bilaterally during walking, making them attractive levers to alter lower-limb loading *in vivo*.

Subject-specific, multi-scale simulations based on a person’s movement data and medical images can be used to quantify growth plate stresses (Kainz et al., 2020). MRI-based musculoskeletal (MSK) models capture each child’s bony geometry and muscle paths, improving anatomical fidelity over generic scaling (Modenese et al., 2021; Scheys et al., 2008). Electromyography (EMG)-informed simulations constrain muscle recruitment toward physiologic activations, yielding more realistic muscle and JCFs than static optimization alone (Lloyd and Besier, 2003). These subject-specific forces can then be applied as loading conditions in finite element (FE) analyses to compute hydrostatic (compressive) and octahedral shear stress distributions at the proximal and distal femoral growth plates that are linked to subsequent bone development (Kainz et al., 2020; Koller et al., 2025b; Lipphaus et al., 2025; Yadav et al., 2017).

Subject-specific MSK and FE pipelines have characterised paediatric growth plate mechanics under functional loading quantified with static optimization, demonstrating regional sensitivity of stresses to joint loading and anatomy (Carriero et al., 2011; Kainz et al., 2020; Koller et al., 2024). Gait-modification studies show that altering foot progression angle can systematically change loading (Uhlrich et al., 2025). However, to the best of our knowledge, no study has integrated MRI-based, EMG-informed MSK modelling to quantify subject-specific JCF magnitude and orientation in children and then propagated these loads to FE analyses of both proximal and distal femoral growth plates.

The current study addresses this knowledge gap and presents a pilot analysis of three healthy children intended to establish feasibility and mechanistic plausibility rather than generalisability. Participants were walking on an instrumented tandem treadmill under preferred gait and different stages of bilateral in-/out-toeing conditions. Using an MRI-based MSK model and EMG-informed optimization algorithm, we quantify how gait modifications alter muscle forces as well as the hip, knee and patellofemoral JCFs. Furthermore, we quantify how these changes relate to regional hydrostatic and octahedral shear stresses within the proximal and distal femoral growth plates. We hypothesised that: (i) in-/out-toeing would produce direction-specific changes in JCF orientation and magnitude compared with preferred gait; and (ii) these changes would be accompanied by shifts in the spatial distribution and magnitude of hydrostatic (compressive) and octahedral shear stresses within the proximal and distal femoral growth plates. By linking modifiable gait patterns to mechanobiologically relevant stress metrics, we aim to establish proof-of-concept for biomechanical regulation of paediatric growth. If supported, these findings would provide a mechanistic basis for personalized, non-surgical strategies, such as gait retraining, to steer growth during maturation.

## 2 Materials and methods

### 2.1 Study design and participants

**FIGURE 1:**
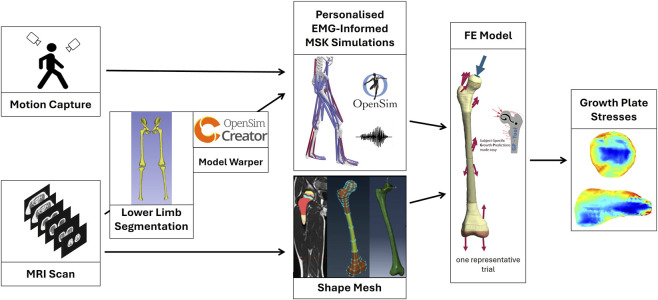
Schematic workflow visualizing the modelling pipeline. MRI scans were used to segment bones and create personalized musculoskeletal (MSK) models using OpenSim Creator. These models and corresponding motion capture data were used to perform EMG-informed simulations to estimate muscle and joint contact forces. These loading conditions were applied to a subject-specific MRI-derived FE model to evaluate stresses within the proximal and distal growth plates.

We conducted a cross-sectional, within-subject pilot study in three healthy typically developing (TD) children (Table 1). Inclusion criteria were the absence of musculoskeletal or neurological disorders. Ethical approval was obtained from University of Vienna’s local ethics committee (#01293), and parental consent with child assent was secured prior to participation. A diagram visualizing the schematic workflow of the modelling pipeline is provided in Figure 1.

**TABLE 1:** Participant information including preferred foot progression angle (FPA) and its standard deviation (SD), femoral anteversion angle (AVA), neck-shaft angle (NSA) and tibial torsion (TT). AVA and NSA were measured according to Sangeux et al. (2015). TT was measured according to Yan et al. (2019).

​	​	​	​	​	​	Left [°]				Right [°]			
ID	Age	Sex	Walking speed [m/s]	Weight [kg]	Height [cm]	FPA ± SD	AVA	NSA	TT	FPA ± SD	AVA	NSA	TT
TD1	9.3	f	1.06	40.1	131.5	3.7 ± 2.0	18.4	135.1	22.1	7.5 ± 2.7	13.4	136.7	28.2
TD2	7.6	f	1.03	26.4	128.5	1.0 ± 2.1	21.7	131.6	30.6	4.3 ± 2.6	8.7	141.6	32.9
TD3	15.1	m	1.22	49.8	173.7	0.4 ± 1.8	17.8	132.5	20.1	2.7 ± 1.6	17.8	131.6	30.8

### 2.2 MRI acquisition and anatomy reconstruction

Lower-limb MRI scans were obtained from each child using a 3T system with T1-weighted sequences at 1.04 mm × 1.04 mm × 1 mm voxel size. Pelvis, femur and tibia geometries were segmented using 3D Slicer (Fedorov et al., 2012). Furthermore, femurs were divided into several parts (proximal/distal trabecular, cortical bone, bone marrow, growth plates), similar to other studies including proximal and distal femoral growth plates (Koller et al., 2024; 2023).

### 2.3 Experimental protocol

Participants walked on an instrumented tandem treadmill (AMTI Inc., Watertown, MA, United States of America). After a 15 min familiarization phase, speed was set within the range of free normalized walking speed proposed by Schwartz et al. (2008) where participants felt comfortable and was held constant during subsequent gait modifications. Conditions included preferred gait, as well as slight and severe bilateral in-toeing and out-toeing. These modifications were coached using target FPAs shown with images. Each condition comprised a familiarisation period (approximately 1 min) followed by continuous steady-state walking of at least 1 min, from which analysis windows were selected. Preferred gait was performed before and after gait modifications.

### 2.4 Motion capture and EMG

Three-dimensional kinematics were recorded using the CGM2.5 marker set (Leboeuf et al., 2019) sampled at 200 Hz with a Vicon (Vicon Motion Systems, Oxford, United Kingdom) motion capture system with eight cameras. Ground reaction forces were simultaneously acquired from the AMTI tandem treadmill’s dual force plates at 1,000 Hz. Additionally, bilateral surface EMG (Cometa Srl, Milan, Italy) was synchronously collected from key lower-limb muscles (i.e., gluteus medius, rectus femoris, vastus medialis, semitendinosus, tibialis anterior, gastrocnemius medialis, soleus, peroneus longus) at 1,000 Hz. Electrodes were placed following the SENIAM guidelines (Hermens et al., 2000). EMG signals were band-pass filtered (20–400 Hz), demeaned, full-wave rectified, low-pass filtered (10 Hz) to form linear envelopes and time shifted by 40 ms to account for the electromechanical delay (Buchanan et al., 2004). Ground reaction forces were low-pass filtered with a fourth order Butterworth filter with a cutoff frequency of 12 Hz.

### 2.5 Musculoskeletal models

The *RajagopalLaiUhlrich* OpenSim model (Rajagopal et al., 2016; Uhlrich et al., 2022) was modified to include a knee with 2 degrees of freedom (flexion/extension and ab-/adduction) as well as an advanced formulation to allow medial and lateral knee loading separately following Lerner et al. (2015). To capture the effect of passive stabilizing structures (i.e. ligaments, joint capsule), a strong actuator (100 Nm at full activation) was added to the knee’s ab-/adduction degree of freedom similar to other studies (Guggenberger et al., 2025; Holder et al., 2020). This actuator provided the required joint moment, thereby eliminating the need for muscle-generated ab-/adduction torque. This model was used as a base model to create subject-specific MRI models using OpenSim Creator’s *Model Warper* v0.6.2 (Kewley et al., 2026).

First, corresponding landmarks were manually selected for each segmented bone (pelvis, femurs, tibias) and the base models’ geometries. Second, all meshes, markers, muscle via- and attachment points, muscle wrap objects and the mass centre of each of those segments was warped using Thin-Plate-Splines (TPS) defined by the selected landmarks similar to Stansfield et al. (2025). The patella segment and corresponding elements were warped with the femur’s TPS. Third, remaining segments (feet, torso, upper body segments) were linearly scaled based on the location of surface markers using OpenSim’s *Scaling Tool* (Delp et al., 2007). Fourth, *Inverse Kinematics* was used to calculate joint angles for all trials. Muscle lengths during all motions were checked for discontinuities and wrap objects eventually modified (Koller et al., 2025a). Finally, the *MuscleParamOptimizer* (Modenese et al., 2016) was applied to adjusted tendon slack length and optimal fibre length, ensuring muscle operating conditions comparable to the base model.

### 2.6 Stride classification based on FPA and representative trial selection

Due to potential variability of FPA during continuous steady-state walking, strides where *post hoc* classified into five groups. FPA was calculated for each trial as the orientation of the calcaneus segment around the global vertical axis quantified with *BodyKinematics* in respect to the walking direction. For each participant, the average FPA ( ${FPA}_{pref}$ ) during the stance phase and its standard deviation ( SD ) across all trials with preferred gait was computed. Then, strides were classified based on its mean FPA ( ${FPA}_{stride}$ ) according to Table 2. Factors for SD were chosen to result in approximately equally distributed stride numbers for each gait modification.

**TABLE 2:** Classification of strides ( ${FPA}_{stride}$ ) based on preferred foot progression angle (***FPA***_***pref***_) and its standard deviation (SD).

Severe in-toeing	${FPA}_{stride}<\;{FPA}_{pref}-SD*5$
Slight in-toeing	${FPA}_{pref}-SD*5<{FPA}_{stride}<\;{FPA}_{pref}-SD*1$
Preferred	${FPA}_{pref}-SD*1<{FPA}_{stride}<\;{FPA}_{pref}+SD*1$
Slight out-toeing	${FPA}_{pref}+SD*1<{FPA}_{stride}<\;{FPA}_{pref}-SD*3$
Severe out-toeing	${FPA}_{pref}+SD*3<{FPA}_{stride}$

For each gait modification class, one representative stride was selected per participant and side that was later used to perform FE analysis. In detail, time-normalized joint angles were concatenated for each stride into one vector. Then, the class’s mean and each stride’s root-mean-square (RMS) deviation to that mean was calculated. The trial with the lowest RMS deviation from the mean kinematics of each gait modification class was chosen as the representative stride.

### 2.7 Musculoskeletal simulations

Musculoskeletal simulations were first performed for the representative strides of preferred gait using OpenSim’s *Static Optimization*, which minimizes the sum of squared muscle activations. For each EMG channel, a scaling factor was calculated based on the maximum estimated muscle activations of the corresponding muscle during the representative stride. This amplitude scaling factor was used throughout all strides to preserve activation differences due to gait modifications.

Finally, muscle activations and forces for all strides were estimated using an EMG-informed modelling approach with the *MuscleRedundancySolver* blending EMG tracking with effort minimisation (De Groote et al., 2016; Falisse et al., 2017). Detailed information of the cost function is included in the Supplementary Material. From these solutions, hip and knee JCFs were computed. To summarize, *Static Optimization* was used for initial estimates of muscle activation amplitudes, while final muscle and JCFs were obtained from the EMG-informed solution.

### 2.8 Finite element simulations

**FIGURE 2:**
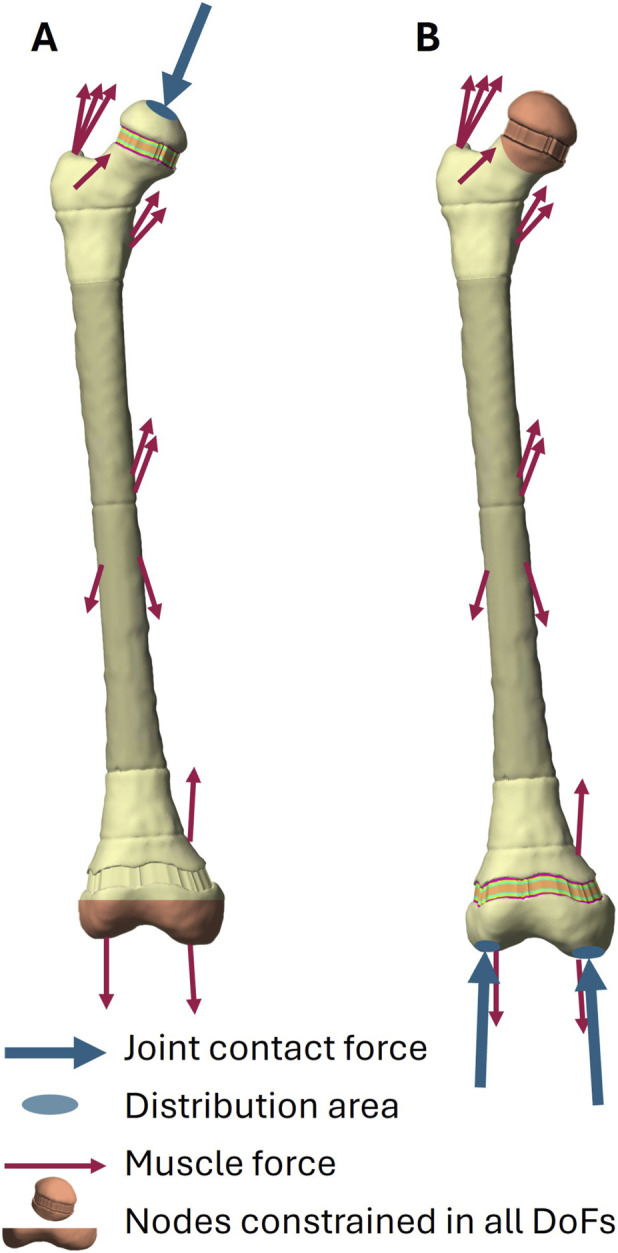
Schematic visualization of FE models showing boundary conditions, muscle force attachment sites and areas where joint contact force was applied for FE models to evaluate stresses within the proximal growth plate **(A)** and distal growth plate **(B)**.

Subject-specific finite element (FE) models of each femur were generated using the GP-Tool (Koller et al., 2023; 2024). The models consisted of linear hexahedral meshes with an element size of approximately 1.5 mm, with element orientations aligned to the principal direction of the growth plates. This element size was found to be reasonable based on a previously performed mesh convergence analysis with the same FE framework (Koller et al., 2023). For each representative trial, nine load instances were selected based on the peaks of knee or hip JCF and the valley in-between during the stance phase as it was done in previous studies (Yadav et al., 2016; Kainz et al., 2020; Koller et al., 2024). At each selected time point, muscle forces were applied as nodal loads to the corresponding muscle-attachment node, and JCFs were uniformly distributed over the closest 100 surface nodes in the direction of the JCF orientation in the FE simulations. To evaluate the stresses within the proximal growth plate, nodes at the distal epicondyles were fully constrained as boundary condition (Yadav et al., 2016). These nodes were selected by identifying those with vertical coordinates in the bottom 5% of the femur’s length. For simulations quantifying stresses in the distal growth plate, nodes at the femoral head were fully constrained (Koller et al., 2024). These nodes were defined as those whose distance from the hip joint centre was less than 1.2 times the femoral head radius. The different femoral regions (cortical bone, trabecular bone, bone marrow, growth plate) were modelled as linear elastic materials with region-specific properties (Supplementary Table S1). FEBio 3.8.0 was used to solve the static FE analyses (Maas et al., 2012). A schematic FE model visualizing the boundary conditions, loading sites of muscle and JCF is provided in Figure 2.

### 2.9 Comparison between gait modifications

All MSK simulation results were time normalized to the stance phase. Joint moments, muscle forces and JCFs were amplitude normalized to the participants’ body weights. Maximum octahedral shear and compressive stresses within the proximal and distal femoral growth plates occurring during the stance phase were visualized as heatmaps and compared qualitatively. The osteogenic index (OI), representing the growth rate due to mechanical stimuli was calculated according to Stevens et al. (1999) and similar to other studies evaluating growth rates with values for *a* and *b* of *0.02* and *0.01*, respectively (Carriero et al., 2011; Kainz et al., 2020; Koller et al., 2023). Minimum hydrostatic stress (i.e. maximum compressive), maximum octahedral shear stress and range of OI values across the growth plates were analysed numerically. Due to the limited number of three participants, results were analysed descriptively without any statistical tests similar to previous studies (Carriero et al., 2011; Kainz et al., 2020; Yadav et al., 2017).

## 3 Results

### 3.1 Kinematics, joint moments, muscle activation and internal forces

All trials were within OpenSim’s best practice recommendations regarding marker tracking errors (<2 cm RMS and <4 cm maximum error). Residual forces and reserve actuator moments were low with <10% of maximum external force and <10% of corresponding joint moment, respectively. Muscle paths showed no discontinuities for all strides. Estimated muscle activations showed close agreement with EMG-measured muscle activity.

**FIGURE 3:**
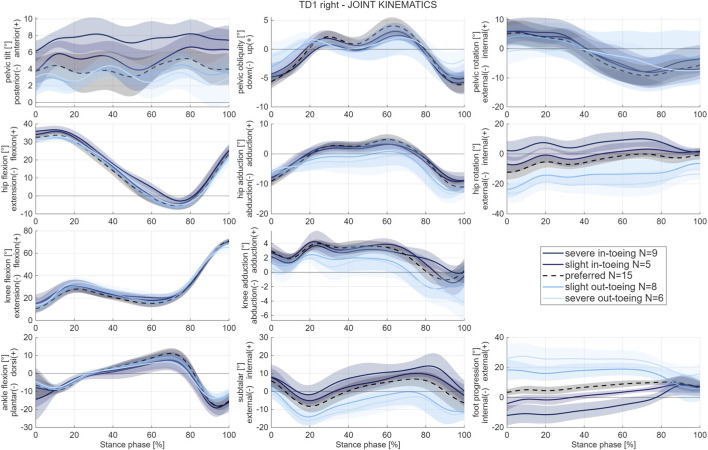
Example of mean and standard deviation (shaded) kinematic waveforms from one participant (TD1, right side). Kinematic waveforms of all participants are provided in the Supplementary Material of this paper.

Classification of strides based on the mean FPA during the stance phase separated trials into five gait modification classes with distinct kinematics. Across all participants, modifying the FPA mainly altered joint kinematics in the transverse plane, i.e. subtalar and hip internal/external rotation angle. Sagittal plane kinematics were similar across conditions except for increased anterior pelvic tilt when participants in-toed. Frontal plane kinematics varied slightly between conditions but without a common trend across participants (Figure 3).

**FIGURE 4:**
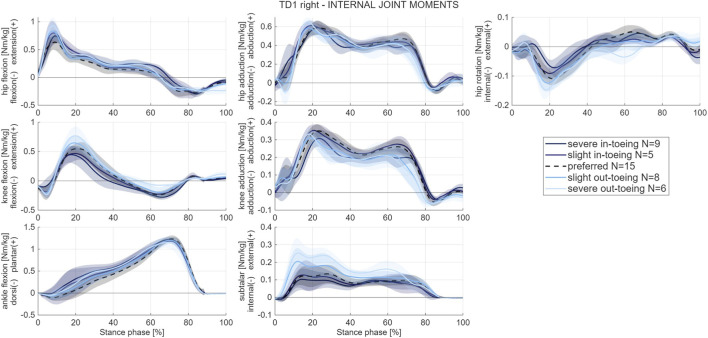
Example of mean and standard deviation (shaded) internal joint moment waveforms from one participant (TD1, right side). Internal joint moments waveforms of all participants are provided in the Supplementary Material of this paper.

A change of the knee flexion moment was found with higher internal extension moments for out-toeing gait patterns in all participants. In these gait modifications, the internal knee abduction moment was lower, especially at the second peak. Furthermore, subtalar joint moments were different between all gait modifications across participants. Other joint moments did not show clear patterns across participants (Figure 4).

**FIGURE 5:**
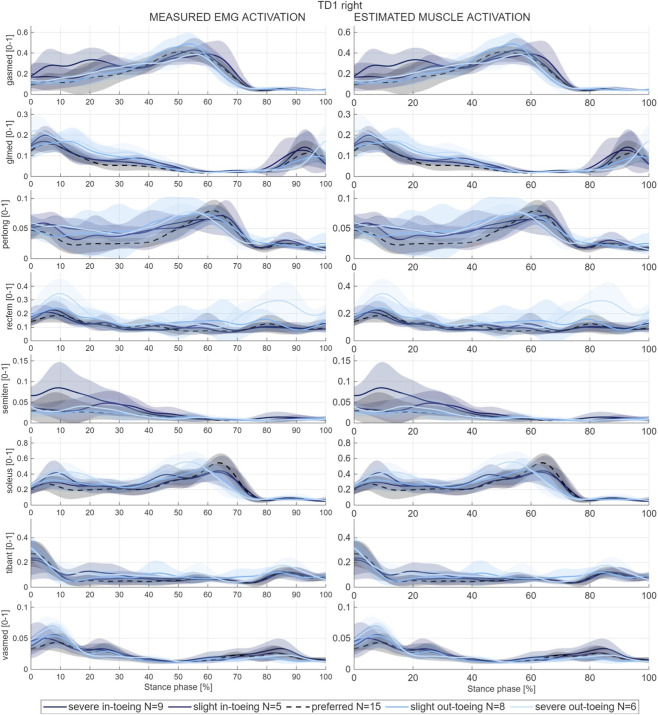
Example of mean and standard deviation (shaded) waveforms of measured muscle activation (left) with electromyography (EMG) compared with the estimated muscle activation (right) of the corresponding muscle from one participant (TD1, right side). EMG signals were amplitude-normalized based on the maximum estimated activation obtained with Static Optimization of the corresponding muscle during the representative preferred-walking stride. The normalized EMG signals were then used to inform the MSK simulations, which contributes to the good agreement between measured and estimated activations. EMG-measured and estimated muscle activation waveforms of all participants are provided in the Supplementary Material of this paper.

Measured muscle activations for muscles responsible for in- and eversion of the foot showed similar patterns in most participants. Activation of peroneus longus was increased when out-toeing (except for TD2 right) whereas semitendinosus was increased when in-toeing (except for TD3 left). Muscle coordination changes measured by EMG were in agreement with altered muscle activations estimated by the MSK simulations across individuals and gait modifications (Figure 5).

Muscle activations estimated by EMG-informed MSK simulation indicate a clear trend of higher tibialis posterior and lower antagonistic activation of the peroneal muscles for in-toeing gait pattern. The anterior component of gluteus minimus (glmin1) substantially increased, whereas the muscle’s posterior compartment (glmin3) was similar or decreased. Furthermore, in-toeing increased gluteus maximus activation. For all other muscles and modifications, no distinct trends were observable across participants, suggesting that muscle coordination during non-preferred walking is subject-specific.

**FIGURE 6:**
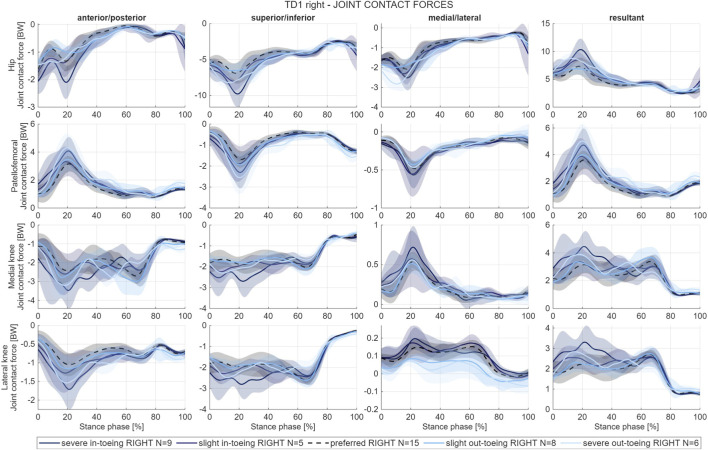
Example of mean and standard deviation (shaded) hip, patellofemoral, medial and lateral knee joint contact force waveforms from one participant (TD1, right side). Hip, patellofemoral, medial and lateral knee joint contact force waveforms of all participants are provided in the Supplementary Material of this paper.

**FIGURE 7:**
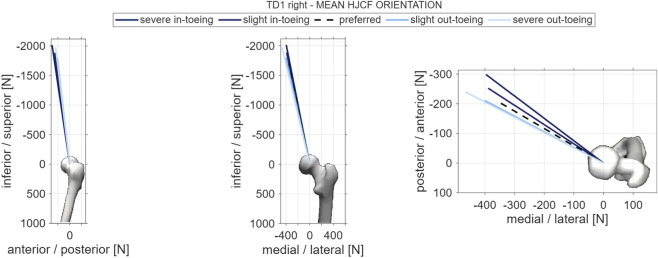
Example orientation vector of the mean resultant hip joint contact force from one participant (TD1, right side). Orientation vectors of the mean resultant hip joint contact force of all participants are provided in the Supplementary Material of this paper.

In-toeing increased maximum resultant hip JCFs compared to preferred and out-toeing gait (Figure 6), potentially due to higher gluteus maximus forces. The direction of the mean hip JCFs was generally more anteriorly in the transverse plane when participants in-toed, most likely resulting from higher forces of the anterior gluteus minimus part (Figure 7). No general pattern across participants was found for the knee and the patellofemoral JCFs, but forces tended to increase in two out of three participants when in-toeing compared to preferred walking.

Detailed figures visualizing the differences of joint angles, internal joint moments, normalized muscle fibre lengths, measured and estimated muscle activations, muscle forces and JCFs between gait modifications for each participant are included in the Supplementary Material (Supplementary Figures S1–S48).

### 3.2 Stresses within the proximal growth plate

**FIGURE 8:**
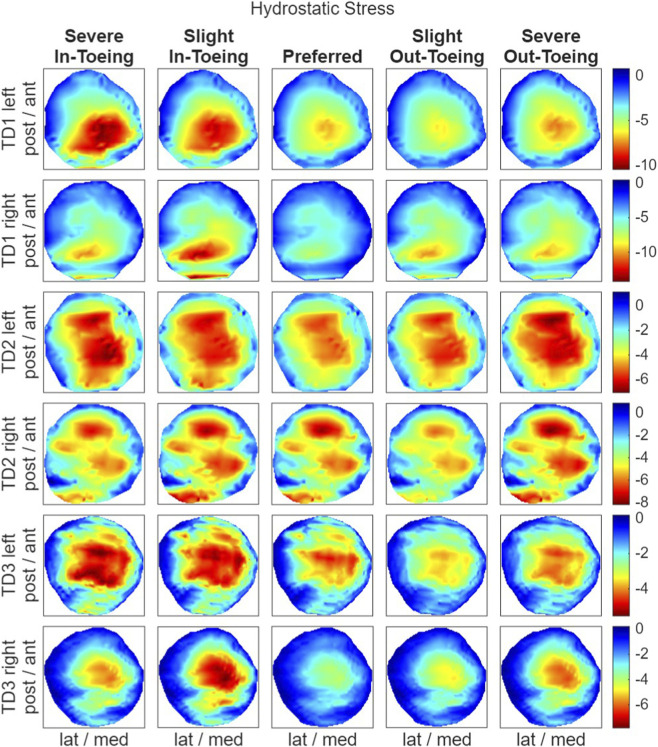
Visualizing the hydrostatic stress [MPa] occurring during the stance phase of the representative strides in the proximal growth plate. Negative values visualized in red indicate compressive stress. All heatmaps within one line represent one side of a participant and have equal colour coding. Right-leg heatmaps were mirrored along the anterior-posterior axis to ensure consistency.

**FIGURE 9:**
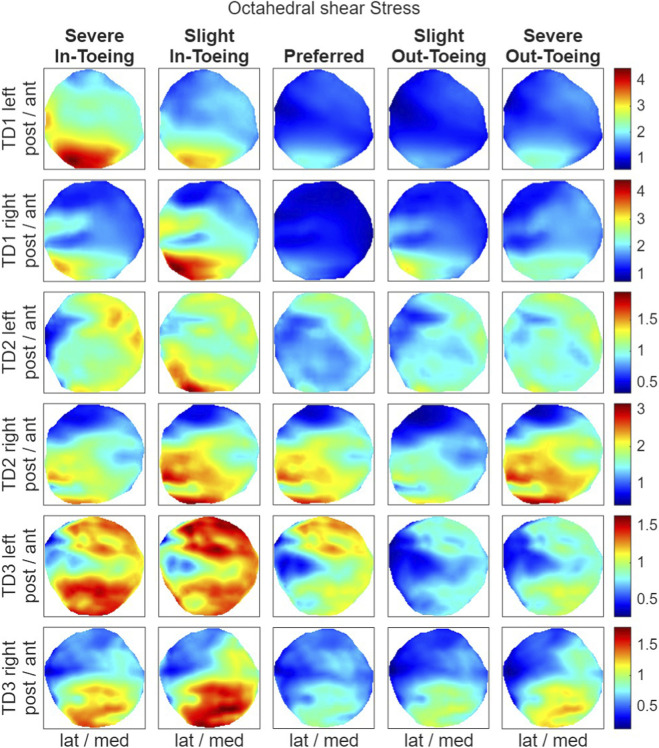
Visualizing the octahedral shear stress [MPa] occurring during the stance phase of the representative strides in the proximal growth plate. All heatmaps within one line represent one side of a participant and have equal colour coding. Right-leg heatmaps were mirrored along the anterior-posterior axis to ensure consistency.

**FIGURE 10:**
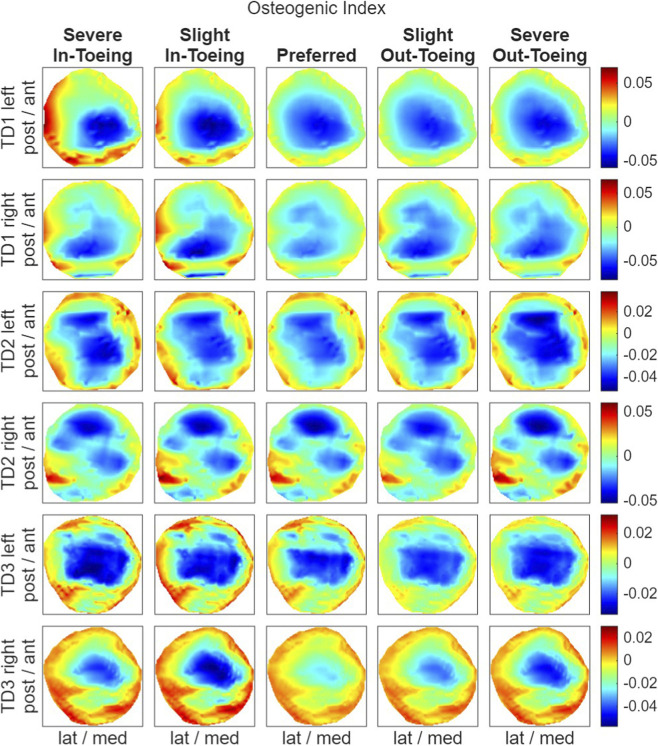
Visualizing the osteogenic index [month^-1^], representing the growth rate due to mechanical stimuli, occurring during the stance phase of the representative strides in the proximal growth plate. All heatmaps within one line represent one side of a participant and have equal colour coding. Right-leg heatmaps were mirrored along the anterior-posterior axis to ensure consistency.

**FIGURE 11:**
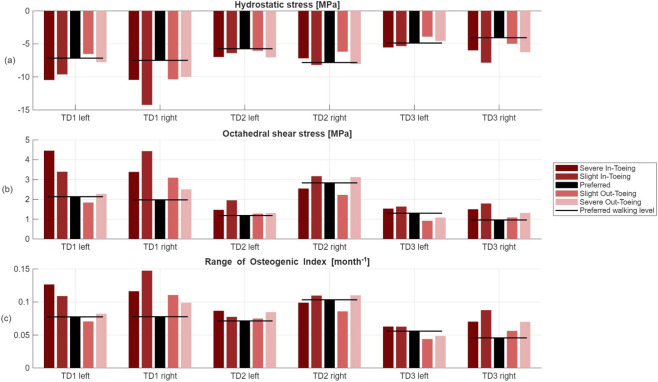
**(a)** Minimum hydrostatic stress, i.e. maximum compressive stress [MPa], **(b)** maximum octahedral shear stress [MPa] and **(c)** range of OI values across the proximal growth plate.

FE simulations revealed a ring-shaped pattern across participants in both hydrostatic stress and OI for the proximal growth plate, whereas the octahedral shear stress did not exhibit this pattern (Figures 8–10). Higher compressive stresses (i.e. more negative hydrostatic stress) within the proximal growth plate were found for in-toeing gait across participants compared to preferred walking (Figure 11a). Also, octahedral shear stresses were higher when in-toeing compared to preferred walking (Figure 11b).

The OI, representing the growth rate due to mechanical stimuli, showed a ring-shape for all gait modification, but was more uniformly distributed during preferred walking than during in-toeing. Numerical analysis confirmed lower range of OI values (except TD2 left) for preferred walking compared to in-toeing (Figure 11c). For out-toeing, no pattern across participants was found. Furthermore, in one participant (TD1), in-toeing clearly increased the OI in the lateral compartment, whereas out-toeing tended to increase OI in the medial compartment.

### 3.3 Stresses within the distal growth plate

**FIGURE 12:**
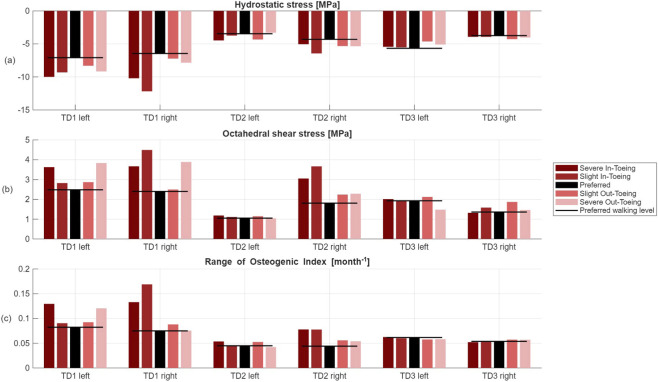
**(a)** Minimum hydrostatic stress, i.e. maximum compressive stress [MPa], **(b)** maximum octahedral shear stress [MPa] and **(c)** range of OI values across the distal growth plate.

**FIGURE 13:**
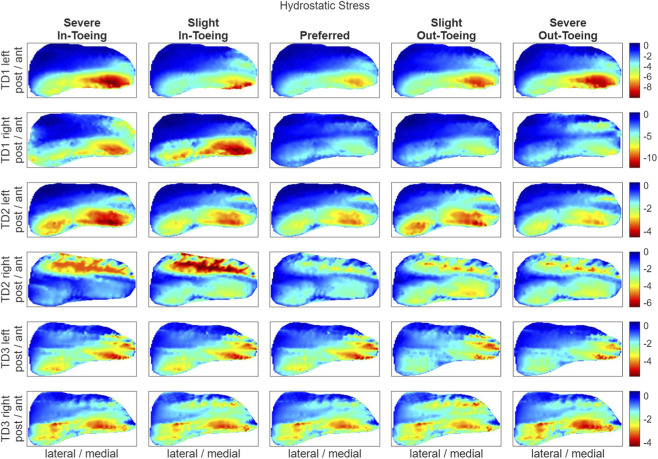
Visualizing the hydrostatic stress [MPa] occurring during the stance phase of the representative strides in the distal growth plate. Negative values visualized in red indicate compressive stress. All heatmaps within one line represent one side of a participant and have equal colour coding. Right-leg heatmaps were mirrored along the anterior-posterior axis to ensure consistency.

**FIGURE 14:**
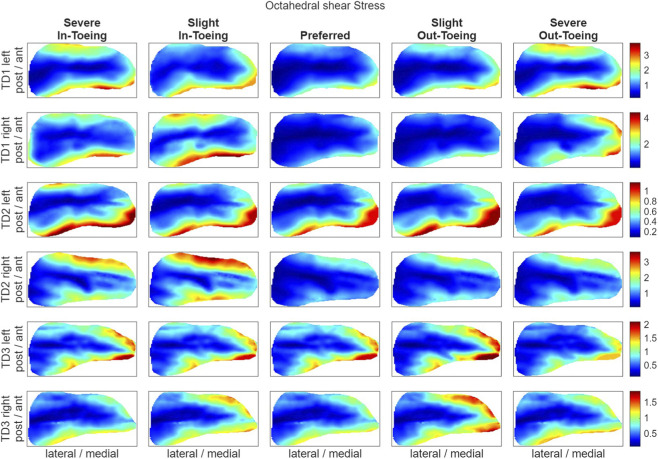
Visualizing the octahedral shear stress [MPa] occurring during the stance phase of the representative strides in the distal growth plate. All heatmaps within one line represent one side of a participant and have equal colour coding. Right-leg heatmaps were mirrored along the anterior-posterior axis to ensure consistency.

Lowest compressive stresses (i.e. less negative hydrostatic stress) in the distal growth plate were generally observed during preferred walking, except for TD3 left (Figure 12a). Both in-toeing and out-toeing increased the compressive stress, predominantly in the posterior region (Figure 13). Preferred gait yielded the lowest octahedral shear stresses magnitudes in two participants (TD1 and TD2) (Figure 12b). Increases in octahedral shear stress were localized to the anterior and posterior edges of the growth plate, with inter-participant variability (Figure 14).

**FIGURE 15:**
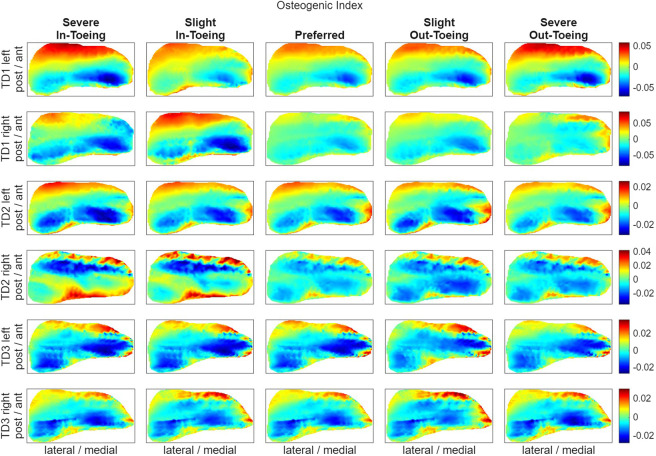
Visualizing the osteogenic index [month^-1^], representing the growth rate due to mechanical stimuli, occurring during the stance phase of the representative strides in the distal growth plate. All heatmaps within one line represent one side of a participant and have equal colour coding. Right-leg heatmaps were mirrored along the anterior-posterior axis to ensure consistency.

In the same two participants (TD1 and TD2), the range of OI values in the distal growth plate was higher during in-toeing than during preferred walking (Figure 12c). The OI shows similar patterns compared to the proximal, i.e. in-toeing shifts OI towards the lateral side and out-toeing shifts OI towards the medial side, in some participants (TD1 right and TD2 left) (Figure 15). However, a general pattern across all participants was not found.

## 4 Discussion

We investigated how gait modifications alter growth-related mechanical stimuli in paediatric femoral growth plates by integrating MRI-based, EMG-informed MSK modelling and subject-specific FE analyses. We found similar changes in kinematics, moments, muscle forces and hip JCF across all participants within our study cohort but growth-related load changes due to gait modifications seem mostly subject-specific.

Our first hypothesis, proposing that direction-specific changes of JCF magnitude and orientation happen across participants, can only be supported for the hip, where in-toeing increased the resultant JCF and shifted its orientation anteriorly in all participants. This may reflect the relatively large changes in hip rotation angles between gait modifications across participants. Because many muscles span the hip, shifts in muscle recruitment are possible. In-toeing increased force in the anterior compartment of the gluteus minimus while decreasing force in its posterior compartment, producing the observed anterior shift in JCF orientation. Alterations at the knee and patellofemoral joint were smaller and more subject-specific. This is in agreement with a recent study from Uhlrich et al. (2025) who showed that a lateralization of knee JCF is achievable with subject-specific modification of the FPA. In detail, some participants had to decrease, whereas others had to increase their FPA to achieve the same results, i.e. shift of knee JCF. Together, our findings and the results from Uhlrich et al. highlight the sensitivity of knee JCF and consequently distal growth plate stress to subtle differences in gait pattern and musculoskeletal geometry.

The second hypothesis, suggesting that growth-related stresses within the femoral growth plates show similar shifts across participants, can be supported for the proximal growth plate but not for the distal growth plate. At the proximal growth plate, in-toeing increased hydrostatic compression and produced a larger range of OI values, indicating a more heterogeneous mechanical growth stimulus. At the distal growth plate, similar results were found but only for two out of three participants. In two legs, in-toeing shifted OI laterally and out-toeing shifted it medially at both the proximal and distal growth plates, suggesting the potential to influence regional growth. Subtle difference in gait pattern, femoral morphology and growth plate shape and orientations might explain the variability in our findings. In the long run, these growth-related stress modifications at the distal growth plate might affect varus/valgus alignment of the knee joint. While these findings support the rationale for personalized gait retraining to influence growth plate loading, the participant-specific variability underscores the need for individualised assessment and intervention planning.

Overall, our participants’ preferred walking kinematics and kinetics were consistent with reports of typically developing children (Schwartz et al., 2008) and the estimated muscle and JCFs showed good agreement in terms of shape and magnitude with prior EMG-informed simulations (Hoang et al., 2019; Kainz et al., 2021b; Koller et al., 2025b). The magnitudes of octahedral shear and hydrostatic stress as well as osteogenic index for preferred walking were similar to previous studies investigating growth plate stresses during walking (Hucke et al., 2023; Kainz et al., 2020; Koller et al., 2024; Yadav et al., 2021; 2016). The ring-shaped OI distribution in the proximal growth plate corroborates previous findings (Kainz et al., 2020; Koller et al., 2023; Yadav et al., 2021), results at the distal growth plate agree with observations of a previous study investigating stresses at the distal growth plate in TD children and children with cerebral palsy (Koller et al., 2024). While findings from the latter study showed that growth-related stresses are highly influenced by the orientation of JCFs, the anterior shift of the hip JCF during in-toeing in our participants likely explains the changes we observed in proximal plate loading.

The changes of joint moments due to FPA modifications generally agree with prior work. Specifically, out-toeing reduced the late-stance peak of the knee adduction moment across all participants, while hip moments showed subject-specific changes (Guo et al., 2007; Seagers et al., 2022; Simic et al., 2011). Furthermore, out-toeing increased the internal knee extension moment in our participants, which has not yet been reported by other studies. Recent studies have shown that individualized FPA modifications can alter knee joint contact forces (Uhlrich et al., 2025; Seagers et al., 2025) whereas we did not observe a uniform trend across participants for knee and patellofemoral JCFs. This discrepancy likely reflects differences in protocol: those studies tailored the FPA direction to each participant (some in-toed, others out-toed), underscoring inter-individual variability. To the best of the authors’ knowledge, no study previously reported the effect of FPA modifications on the hip JCF during walking.

The findings of this study have clinical relevance to managing torsional and frontal malalignments in the future. Our results indicate that growth-related loads across proximal and distal growth plates are altered by FPA and may influence subsequent bone development, with implications for conditions such as increased femoral anteversion angle and knee varus/valgus alignment. Established surgical approaches, such as hemiepiphysiodesis for correcting knee varus/valgus, operate on the same principle of stress redistribution (Hucke et al., 2023; Stevens, 2007). Furthermore, a study recently showed that gait modifications, i.e., modifying FPA, are a powerful intervention to achieve long-term benefits in people with osteoarthritis (Uhlrich et al., 2025). However, whether and to what extent FPA-induced changes in physeal loading effectively modulate bone growth must be determined in longitudinal studies. In skeletally immature children, gait modification may be considered a low-risk, non-invasive strategy to redistribute loads and potentially guide bone growth potentially avoiding surgery in the long run.

This study has several limitations that need to be mentioned. First, we only included three participants which impacts the generalisability of the findings. However, our results show the feasibility of altering growth plate stresses with gait retraining. Second, we only included healthy participants in this study. Nevertheless, this does not undermine the study design or interpretation, as the used methods accounts for subject-specific MSK morphology and neuromuscular control. The coached gait modifications are practical and are likely feasible in patients without neurological pathologies, e.g., children with idiopathic bony deformities. Third, we scaled the amplitudes of EMG signals based on the results of *Static Optimization* due to the lack of (near) maximum voluntary contraction trials. However, using these obtained scaling factors for all trials ensured that differences in muscle activation levels between gait modifications were maintained in MSK simulations (Supplementary Figures S6, S14, S22, S30, S38, S46). Fourth, we estimated muscle and joint contact forces using MSK simulations because *in vivo* quantification is not feasible non-invasively. Several studies comparing estimated and measured *in vivo* loads report similar waveform patterns (Imani Nejad et al., 2020; Lerner et al., 2015; Modenese et al., 2021). While absolute muscle and JCF magnitudes may not be perfectly accurate, our analyses rely on within-subject, within-session contrasts with identical modelling parameters. Thus, any systematic bias is shared across conditions, and the reported changes are expected to reflect the true direction of effect. Fifth, we employed commonly used, simplified boundary conditions to evaluate stresses in the proximal and distal femoral growth plates, consistent with prior FE studies (Kainz et al., 2020; Koller et al., 2024; Yadav et al., 2021; 2017; 2016). A more physiological boundary condition proposed by Bavil et al. (2024) restricts motion of nodes close to the growth plates. These constraints can attenuate or distort intraphyseal stress fields and are therefore not well suited for mechanobiological analyses that rely on growth plate stresses. Nevertheless, we cannot exclude that more physiologically precise boundary conditions would influence the magnitude or spatial distribution of the computed stresses. Sixth, bone as well as cartilaginous regions (i.e., growth plates) are hierarchical, highly heterogeneous and anisotropic materials with complex, time- and density-dependent behaviour. For our focus on comparative stress patterns, we modelled tissues as linear elastic and isotropic based on literature values. A recently published density-shape model based on a paediatric bone data set (Xu et al., 2025) could support subject- or region-specific elastic moduli to improve material representation. Nonetheless, prior work indicates that the workflow is relatively robust to variations in the stiffness of the surrounding bone (Koller et al., 2023), suggesting limited impact on the principal stress-pattern conclusions. However, the influence of modelling the growth plate as viscoelastic, biphasic compared to a linear-elastic material has not yet been evaluated in detail. In our simulations, we target short-time stance-phase loading where growth plate response is dominated by interstitial fluid pressurization and is effectively near-incompressible. Hence, we assume our choice of linear elastic material with a high Poisson’s ratio (i.e., ν = 0.49) is appropriate for our modelling purpose (Villemure and Stokes, 2009). Nevertheless, future studies should model the growth plate with a viscoelastic and biphasic behaviour and evaluate its impact on growth predictions. Seventh, we investigated only gait modifications targeting the FPA. Other gait modifications, such as variations of stance width, step length or walking speed, potentially also modify growth-related loads and should be investigated in future studies. Eighth, our testing was performed on an instrumented treadmill, which may differ from natural overground gait. At matched speeds and after familiarization, kinematic and kinetic differences between treadmill and overground walking are small and often within test–retest repeatability values (Riley et al., 2007). We included a 15-min general and condition-specific familiarization, supporting the validity of our measurements. However, modifications of FPA may behave differently overground where speed and direction are self-selected. Therefore, future work should include overground trials at self-selected speeds to confirm generalization of our treadmill findings. Ninth, we combined several measurement methods with known uncertainties (e.g., marker misplacement, electrode misplacement, …) that might have influenced MSK simulation results and subsequently growth-related stresses. However, all gait conditions were recorded within a single session without removing markers or electrodes, so any static placement error was shared across conditions mitigating their impact on within subject contrasts that were analysed within this study. Nonetheless, movement dependent artefacts may vary with the gait modification and cannot be fully excluded. To minimize and monitor error, we used standardized marker placement by experienced operators, SENIAM based EMG placement with skin preparation, and we quantified simulation quality (e.g., IK marker errors, residuals/reserves) as detailed in the methods section. These checks indicated acceptable data and simulation quality.

To conclude, this study provides initial evidence that coachable gait cues, particularly in-toeing, can acutely increase hip JCF and alter compressive and shear stresses in the proximal femoral growth plate of children. Responses in the distal growth plate were more variable, potentially due to little and subject-specific changes of muscle forces as well as knee and patellofemoral JCF. The multi-scale MRI-based, EMG-informed MSK and FE pipeline proved to be feasible and sensitive to subject-specific neuromechanical changes, supporting the concept of personalised gait retraining as a non-invasive treatment strategy to influence growth-related mechanical loading. Nonetheless, the between-subject variability in our cohort warrants careful preassessment and highlights the importance of individualized evaluation. Larger, longitudinal studies with refined mechanobiological metrics are necessary to determine whether these acute changes can be harnessed to steer paediatric bone development.

## Data Availability

The datasets presented in this article are not readily available because the raw MRI scans cannot be shared. The data supporting the conclusions of this article will be made available by the authors, without undue reservation. Requests to access the datasets should be directed to WK, willi.koller@univie.ac.at.

## References

[B1] Alexander N. Studer K. Lengnick H. Payne E. Klima H. Wegener R. (2019). The impact of increased femoral antetorsion on gait deviations in healthy adolescents. J. Biomech. 86, 167–174. 10.1016/j.jbiomech.2019.02.005 30799079

[B2] Bavil A. Y. Eghan-Acquah E. Diamond L. E. Barrett R. Carty C. P. Barzan M. (2024). Effect of different constraining boundary conditions on simulated femoral stresses and strains during gait. Sci. Rep. 14, 10808. 10.1038/s41598-024-61305-x 38734763 PMC11088641

[B3] Bobroff E. D. Chambers H. G. Sartoris D. J. Wyatt M. P. Sutherland D. H. (1999). Femoral anteversion and neck-shaft angle in children with cerebral palsy. Clin. Orthop. 364, 194–204. 10.1097/00003086-199907000-00025 10416409

[B4] Bruderer‐Hofstetter M. Fenner V. Payne E. Zdenek K. Klima H. Wegener R. (2015). Gait deviations and compensations in pediatric patients with increased femoral torsion. J. Orthop. Res. 33, 155–162. 10.1002/jor.22746 25284013

[B5] Buchanan T. S. Lloyd D. G. Manal K. Besier T. F. (2004). Neuromusculoskeletal modeling: estimation of muscle forces and joint moments and movements from measurements of neural command. J. Appl. Biomech. 20, 367–395. 10.1123/jab.20.4.367 16467928 PMC1357215

[B6] Carriero A. Jonkers I. Shefelbine S. J. (2011). Mechanobiological prediction of proximal femoral deformities in children with cerebral palsy. Comput. Methods Biomech. Biomed. Engin. 14, 253–262. 10.1080/10255841003682505 20229379

[B7] Carter D. R. Wong M. (1988). The role of mechanical loading histories in the development of diarthrodial joints. J. Orthop. Res. 6, 804–816. 10.1002/jor.1100060604 3171761

[B8] De Groote F. Kinney A. L. Rao A. V. Fregly B. J. (2016). Evaluation of direct collocation optimal control problem formulations for solving the muscle redundancy problem. Ann. Biomed. Eng. 44, 2922–2936. 10.1007/s10439-016-1591-9 27001399 PMC5043004

[B9] Delp S. L. Anderson F. C. Arnold A. S. Loan P. Habib A. John C. T. (2007). OpenSim: open-source software to create and analyze dynamic simulations of movement. IEEE Trans. Biomed. Eng. 54, 1940–1950. 10.1109/TBME.2007.901024 18018689

[B10] Diamond L. E. Devaprakash D. Cornish B. Plinsinga M. L. Hams A. Hall M. (2022). Feasibility of personalised hip load modification using real-time biofeedback in hip osteoarthritis: a pilot study. Osteoarthr. Cartil. Open 4, 100230. 10.1016/j.ocarto.2021.100230 36474469 PMC9718151

[B11] Falisse A. Van Rossom S. Jonkers I. De Groote F. (2017). EMG-driven optimal estimation of Subject-SPECIFIC hill model muscle–tendon parameters of the knee joint actuators. IEEE Trans. Biomed. Eng. 64, 2253–2262. 10.1109/TBME.2016.2630009 27875132

[B12] Fedorov A. Beichel R. Kalpathy-Cramer J. Finet J. Fillion-Robin J.-C. Pujol S. (2012). 3D slicer as an image computing platform for the quantitative imaging network. Magn. Reson. Imaging 30, 1323–1341. 10.1016/j.mri.2012.05.001 22770690 PMC3466397

[B13] Frost H. M. (2001). From Wolff’s law to the Utah paradigm: insights about bone physiology and its clinical applications. Anat. Rec. 262, 398–419. 10.1002/ar.1049 11275971

[B14] Guggenberger B. Koller W. Habersack A. Kraus T. Sperl M. Svehlik M. (2025). Impact of femoral and tibial torsion on patellofemoral loading in individuals with patellofemoral instability. J. Orthop. Res. 43, 973–983. 10.1002/jor.26058 39993930 PMC11982600

[B15] Guo M. Axe M. J. Manal K. (2007). The influence of foot progression angle on the knee adduction moment during walking and stair climbing in pain free individuals with knee osteoarthritis. Gait Posture 26, 436–441. 10.1016/j.gaitpost.2006.10.008 17134902

[B16] Hermens H. J. Freriks B. Disselhorst-Klug C. Rau G. (2000). Development of recommendations for SEMG sensors and sensor placement procedures. J. Electromyogr. Kinesiol. 10, 361–374. 10.1016/S1050-6411(00)00027-4 11018445

[B17] Hoang H. X. Diamond L. E. Lloyd D. G. Pizzolato C. (2019). A calibrated EMG-informed neuromusculoskeletal model can appropriately account for muscle co-contraction in the estimation of hip joint contact forces in people with hip osteoarthritis. J. Biomech. 83, 134–142. 10.1016/j.jbiomech.2018.11.042 30527636

[B18] Holder J. Feja Z. Van Drongelen S. Adolf S. Böhm H. Meurer A. (2020). Effect of guided growth intervention on static leg alignment and dynamic knee contact forces during gait. Gait Posture 78, 80–88. 10.1016/j.gaitpost.2020.03.012 32298950

[B19] Hucke L. Holder J. Van Drongelen S. Stief F. Gámez A. J. Huß A. (2023). Influence of tension-band plates on the mechanical loading of the femoral growth plate during guided growth due to coronal plane deformities. Front. Bioeng. Biotechnol. 11, 1165963. 10.3389/fbioe.2023.1165963 37415789 PMC10321528

[B20] Imani Nejad Z. Khalili K. Hosseini Nasab S. H. Schütz P. Damm P. Trepczynski A. (2020). The capacity of generic musculoskeletal simulations to predict knee joint loading using the CAMS-knee datasets. Ann. Biomed. Eng. 48, 1430–1440. 10.1007/s10439-020-02465-5 32002734 PMC7089909

[B21] Kainz H. Killen B. A. Wesseling M. Perez-Boerema F. Pitto L. Garcia Aznar J. M. (2020). A multi-scale modelling framework combining musculoskeletal rigid-body simulations with adaptive finite element analyses, to evaluate the impact of femoral geometry on hip joint contact forces and femoral bone growth. PLOS ONE 15, e0235966. 10.1371/journal.pone.0235966 32702015 PMC7377390

[B22] Kainz H. Killen B. A. Van Campenhout A. Desloovere K. Garcia Aznar J. M. Shefelbine S. (2021a). ESB clinical biomechanics award 2020: pelvis and hip movement strategies discriminate typical and pathological femoral growth – insights gained from a multi-scale mechanobiological modelling framework. Clin. Biomech. 87, 105405. 10.1016/j.clinbiomech.2021.105405 34161909

[B23] Kainz H. Wesseling M. Jonkers I. (2021b). Generic scaled *versus* subject-specific models for the calculation of musculoskeletal loading in cerebral palsy gait: effect of personalized musculoskeletal geometry outweighs the effect of personalized neural control. Clin. Biomech. 87, 105402. 10.1016/j.clinbiomech.2021.105402 34098149

[B24] Kewley A. van Beesel J. Seth A. (2026). OpenSim creator: an interactive user interface for building OpenSim musculoskeletal models. J. Open Source Softw. 11, 8284. 10.21105/joss.08284

[B25] Koller W. Gonçalves B. Baca A. Kainz H. (2023). Intra- and inter-subject variability of femoral growth plate stresses in typically developing children and children with cerebral palsy. Front. Bioeng. Biotechnol. 11, 1140527. 10.3389/fbioe.2023.1140527 36911204 PMC9999378

[B26] Koller W. Wallnöfer E. Holder J. Kranzl A. Mindler G. Baca A. (2024). ESMAC best paper award 2023: increased knee flexion in participants with cerebral palsy results in altered stresses at the distal femoral growth plate compared to a typically developing cohort. Gait Posture 113, 158–166. 10.1016/j.gaitpost.2024.06.012 38905850

[B27] Koller W. Horsak B. Kranzl A. Unglaube F. Baca A. Kainz H. (2025a). Physiological plausible muscle paths: a MATLAB tool for detecting and resolving muscle path discontinuities in musculoskeletal OpenSim models. Gait Posture 117, S21–S22. 10.1016/j.gaitpost.2025.01.063

[B28] Koller W. Svehlik M. Wallnöfer E. Kranzl A. Mindler G. Baca A. (2025b). Femoral bone growth predictions based on personalized multi-scale simulations: validation and sensitivity analysis of a mechanobiological model. Biomech. Model. Mechanobiol. 24, 879–894. 10.1007/s10237-025-01942-x 40227492 PMC12162800

[B29] Kronenberg H. M. (2003). Developmental regulation of the growth plate. Nature 423, 332–336. 10.1038/nature01657 12748651

[B30] Leboeuf F. Baker R. Barré A. Reay J. Jones R. Sangeux M. (2019). The conventional gait model, an open-source implementation that reproduces the past but prepares for the future. Gait Posture 69, 235–241. 10.1016/j.gaitpost.2019.04.015 31027876

[B31] Lerner Z. F. DeMers M. S. Delp S. L. Browning R. C. (2015). How tibiofemoral alignment and contact locations affect predictions of medial and lateral tibiofemoral contact forces. J. Biomech. 48, 644–650. 10.1016/j.jbiomech.2014.12.049 25595425 PMC4330122

[B32] Lipphaus A. Tröbs R.-B. Klimek M. Selkmann S. Witzel U. , (2025). Computational mechanobiological model combining epiphyseal, apophyseal, and appositional growth and inner bone remodeling of the juvenile femur. 10.21203/rs.3.rs-7224633/v1 PMC13374303 42458460

[B33] Lloyd D. G. Besier T. F. (2003). An EMG-driven musculoskeletal model to estimate muscle forces and knee joint moments *in vivo* . J. Biomech. 36, 765–776. 10.1016/S0021-9290(03)00010-1 12742444

[B34] Maas S. A. Ellis B. J. Ateshian G. A. Weiss J. A. (2012). FEBio: finite elements for biomechanics. J. Biomech. Eng. 134, 011005. 10.1115/1.4005694 22482660 PMC3705975

[B35] Modenese L. Ceseracciu E. Reggiani M. Lloyd D. G. (2016). Estimation of musculotendon parameters for scaled and subject specific musculoskeletal models using an optimization technique. J. Biomech. 49, 141–148. 10.1016/j.jbiomech.2015.11.006 26776930

[B36] Modenese L. Barzan M. Carty C. P. (2021). Dependency of lower limb joint reaction forces on femoral version. Gait Posture 88, 318–321. 10.1016/j.gaitpost.2021.06.014 34246172

[B37] Rajagopal A. Dembia C. L. DeMers M. S. Delp D. D. Hicks J. L. Delp S. L. (2016). Full-body musculoskeletal model for muscle-driven simulation of human gait. IEEE Trans. Biomed. Eng. 63, 2068–2079. 10.1109/TBME.2016.2586891 27392337 PMC5507211

[B38] Riley P. O. Paolini G. Della Croce U. Paylo K. W. Kerrigan D. C. (2007). A kinematic and kinetic comparison of overground and treadmill walking in healthy subjects. Gait Posture 26, 17–24. 10.1016/j.gaitpost.2006.07.003 16905322

[B39] Sangeux M. Pascoe J. Graham H. K. Ramanauskas F. Cain T. (2015). Three-dimensional measurement of femoral neck anteversion and neck shaft angle. J. Comput. Assist. Tomogr. 39, 83–85. 10.1097/RCT.0000000000000161 25354092

[B40] Scheys L. Van Campenhout A. Spaepen A. Suetens P. Jonkers I. (2008). Personalized MR-based musculoskeletal models compared to rescaled generic models in the presence of increased femoral anteversion: effect on hip moment arm lengths. Gait Posture 28, 358–365. 10.1016/j.gaitpost.2008.05.002 18571416

[B41] Schwartz M. H. Rozumalski A. Trost J. P. (2008). The effect of walking speed on the gait of typically developing children. J. Biomech. 41, 1639–1650. 10.1016/j.jbiomech.2008.03.015 18466909

[B42] Seagers K. Uhlrich S. D. Kolesar J. A. Berkson M. Kaneda J. M. Beaupre G. S. (2022). Changes in foot progression angle during gait reduce the knee adduction moment and do not increase hip moments in individuals with knee osteoarthritis. J. Biomech. 141, 111204. 10.1016/j.jbiomech.2022.111204 35772243 PMC9466647

[B43] Seagers K. Kolesar J. A. Mazzoli V. Halilaj E. Delp S. L. Uhlrich S. D. (2025). Immediate reductions in compressive and shear forces in the knee from gait retraining are associated with slowed cartilage degeneration after 1 year in medial knee osteoarthritis: a retrospective observational cohort study. Osteoarthr. Cartil. 34, 614–622. 10.1016/j.joca.2025.12.007 41389909 PMC13195794

[B44] Simic M. Hinman R. S. Wrigley T. V. Bennell K. L. Hunt M. A. (2011). Gait modification strategies for altering medial knee joint load: a systematic review. Arthritis Care Res. 63, 405–426. 10.1002/acr.20380 20981808

[B45] Stansfield E. Koller W. Gonçalves B. Kainz H. , (2025). A workflow to create personalised musculoskeletal models based on magnetic resonance images. 10.1101/2025.07.23.666289 PMC13029800 41838666

[B46] Stevens P. M. (2007). Guided growth for angular correction: a preliminary series using a tension band plate. J. Pediatr. Orthop. 27, 253–259. 10.1097/BPO.0b013e31803433a1 17414005

[B47] Stevens S. S. Beaupré G. S. Carter D. R. (1999). Computer model of endochondral growth and ossification in long bones: biological and mechanobiological influences. J. Orthop. Res. 17, 646–653. 10.1002/jor.1100170505 10569472

[B48] Uhlrich S. D. Jackson R. W. Seth A. Kolesar J. A. Delp S. L. (2022). Muscle coordination retraining inspired by musculoskeletal simulations reduces knee contact force. Sci. Rep. 12, 9842. 10.1038/s41598-022-13386-9 35798755 PMC9262899

[B49] Uhlrich S. D. Mazzoli V. Silder A. Finlay A. K. Kogan F. Gold G. E. (2025). Personalised gait retraining for medial compartment knee osteoarthritis: a randomised controlled trial. Lancet Rheumatol. 7, e708–e718. 10.1016/S2665-9913(25)00151-1 40816302 PMC13034617

[B50] Villemure I. Stokes I. A. F. (2009). Growth plate mechanics and mechanobiology. A survey of present understanding. J. Biomech. 42, 1793–1803. 10.1016/j.jbiomech.2009.05.021 19540500 PMC2739053

[B51] Wolff J. (1892). Das Gesetz der Transformation der Knochen (The Law of Bone Remodelling). Berl. Ger. Springer-Verl.

[B52] Xu Y. Brüling J. Carman L. Yeung T. Besier T. F. Choisne J. (2025). A statistical shape and density model can accurately predict bone morphology and regional femoral bone mineral density variation in children. Bone 193, 117419. 10.1016/j.bone.2025.117419 39892636

[B53] Yadav P. Shefelbine S. J. Gutierrez-Farewik E. M. (2016). Effect of growth plate geometry and growth direction on prediction of proximal femoral morphology. J. Biomech. 49, 1613–1619. 10.1016/j.jbiomech.2016.03.039 27063249

[B54] Yadav P. Shefelbine S. J. Pontén E. Gutierrez-Farewik E. M. (2017). Influence of muscle groups’ activation on proximal femoral growth tendency. Biomech. Model. Mechanobiol. 16, 1869–1883. 10.1007/s10237-017-0925-3 28639152 PMC5671539

[B55] Yadav P. Fernández M. P. Gutierrez-Farewik E. M. (2021). Influence of loading direction due to physical activity on proximal femoral growth tendency. Med. Eng. Phys. S1350453321000217. 90, 83–91. 10.1016/j.medengphy.2021.02.008 33781483

[B56] Yan W. Xu X. Xu Q. Yan W. Sun Z. Jiang Q. (2019). Femoral and tibial torsion measurements based on EOS imaging compared to 3D CT reconstruction measurements. Ann. Transl. Med. 7, 460. 10.21037/atm.2019.08.49 31700896 PMC6803189

