# Supplementary material for: Foot progression angle modifications alter hip joint contact forces and femoral growth plate mechanics in children
Source: Front Bioeng Biotechnol. 2026 Apr 28;14:1799347. doi: 10.3389/fbioe.2026.1799347 (PMC13161070; doi:10.3389/fbioe.2026.1799347)
Supplement: Supplementary file 1 [file DataSheet1.pdf]

Supplementary Material

Foot Progression Angle Modifications Alter Hip Joint Contact Forces and Femoral Growth-Plate Mechanics in Children

Willi Koller*, Elias Wallnöfer, Laura Rathmair, Clara Egner, Kevin Kitir, Hans Kainz

*** Correspondence:** Corresponding Author: willi.koller@univie.ac.at

Table of contents

1 EMG-informed simulations with MuscleRedundancySolver 2

2 Material properties for finite element simulations 2

3 Detailed results for TD1 – left side 3

4 Detailed results for TD1 – right side 9

5 Detailed results for TD2 – left side 15

6 Detailed results for TD2 – right side 21

7 Detailed results for TD3 – left side 27

8 Detailed results for TD3 – right side 33

1 EMG-informed simulations with MuscleRedundancySolver

We estimated muscle forces for each stride with the MuscleRedundancySolver (De Groote et al., 2016; Falisse et al., 2017) by minimizing a tracking–effort objective over the stance phase:

$$J = wA \cdot \int \left(e^2(t) + a^2(t)\right) + wT_{res} \cdot \int {a_T}^2 \ (t) + wEMG \cdot \int \left(e(t) - \hat{e}(t)\right)^2 \qquad (1)$$

- Effort term (wA): $e(t)$ is the estimated muscle excitation and $a(t)$ the estimated activation
- Reserve penalty (wT_{res}): $a_T(t)$ is the activation of reserve actuators
- EMG tracking ($wEMG$): $\hat{e}(t)$ is the processed EMG

Weights were set to $wA = 1$, $wT_{res} = 10\ 000$, and $wEMG = 10$. We limited the deviation between the estimated excitation and the scaled EMG to 0.2 at every time step (i.e., $|\ e(t) - \hat{e}(t)\ | \leq 0.2$), allowing minor shape differences while maintaining agreement with EMG.

De Groote, F., Kinney, A.L., Rao, A.V., Fregly, B.J., 2016. Evaluation of Direct Collocation Optimal Control Problem Formulations for Solving the Muscle Redundancy Problem. Ann. Biomed. Eng. 44, 2922–2936. https://doi.org/10.1007/s10439-016-1591-9

Falisse, A., Van Rossom, S., Jonkers, I., De Groote, F., 2017. EMG-Driven Optimal Estimation of Subject-SPECIFIC Hill Model Muscle–Tendon Parameters of the Knee Joint Actuators. IEEE Trans. Biomed. Eng. 64, 2253–2262. https://doi.org/10.1109/TBME.2016.2630009

2 Material properties for finite element simulations

Table S1: Assigned linear elastic material properties for femur regions

**Anatomical structure**	**Youngs' modulus [MPa]**	**Poisson's ratio**
growth plate	1000	0.49
trabecular bone	10 000	0.3
cortical bone	20 000	0.3
bone marrow	100	0.3

3 Detailed results for TD1 – left side

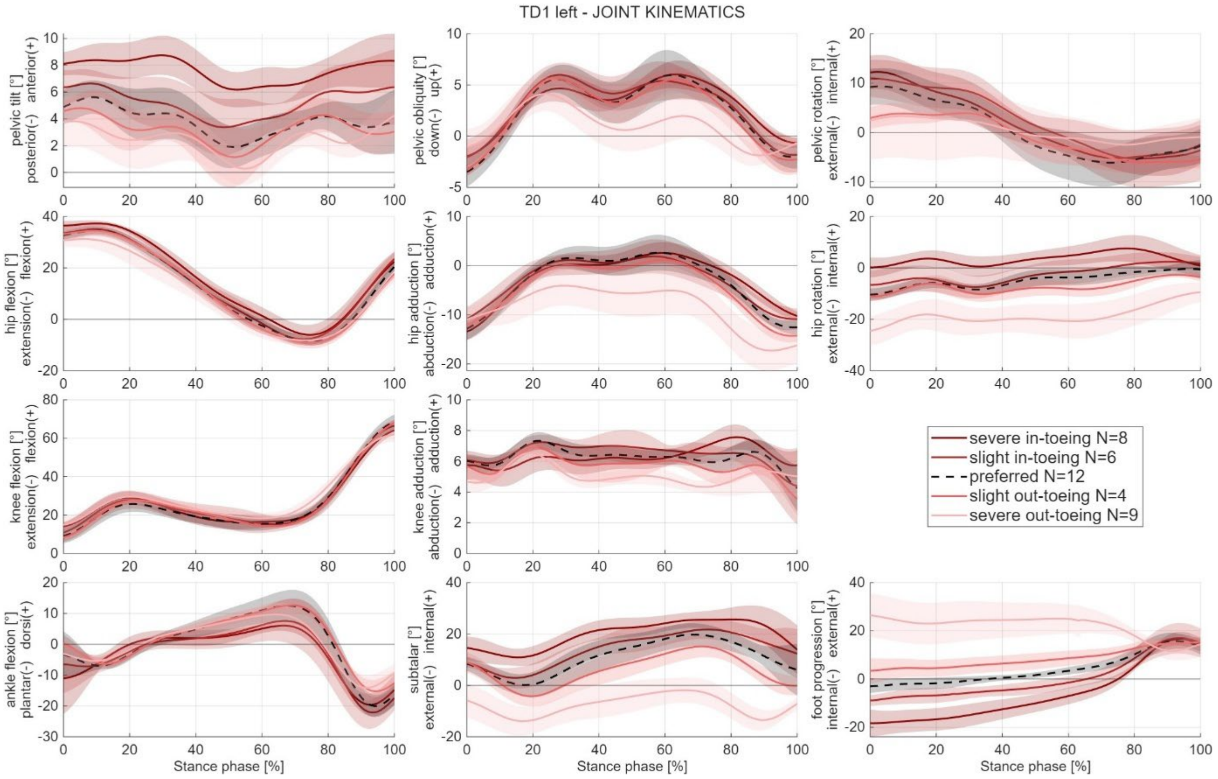

Figure S1: Mean and standard deviation (shaded) of joint angles quantified with OpenSim's Inverse Kinematics

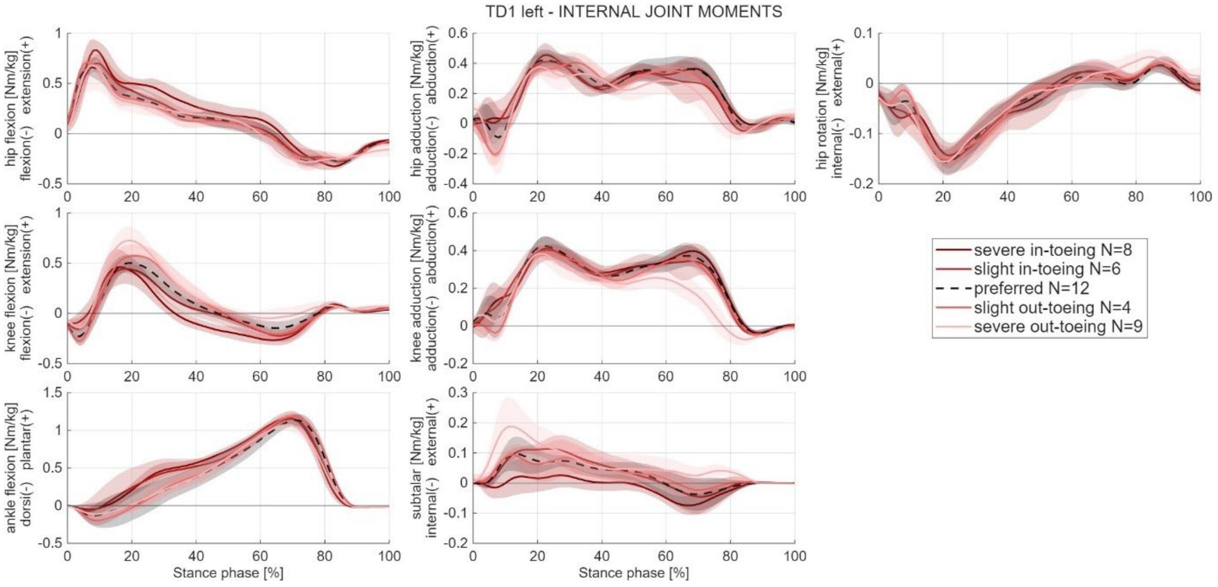

Figure S2: Mean and standard deviation (shaded) of internal joint moments quantified with OpenSim's Inverse Dynamics

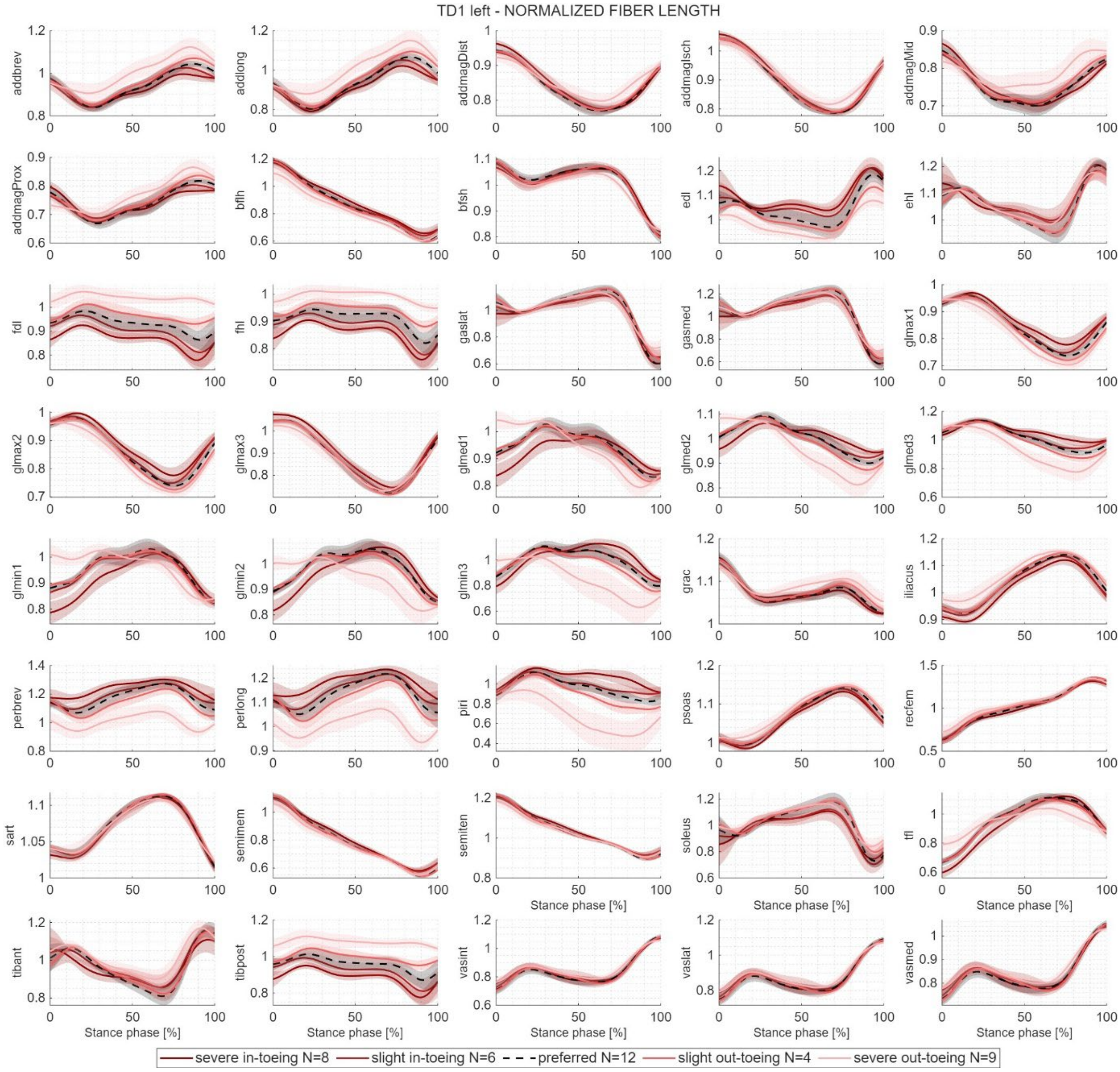

Figure S3: Mean and standard deviation (shaded) of normalized muscle fiber length quantified with OpenSim's Muscle Analysis

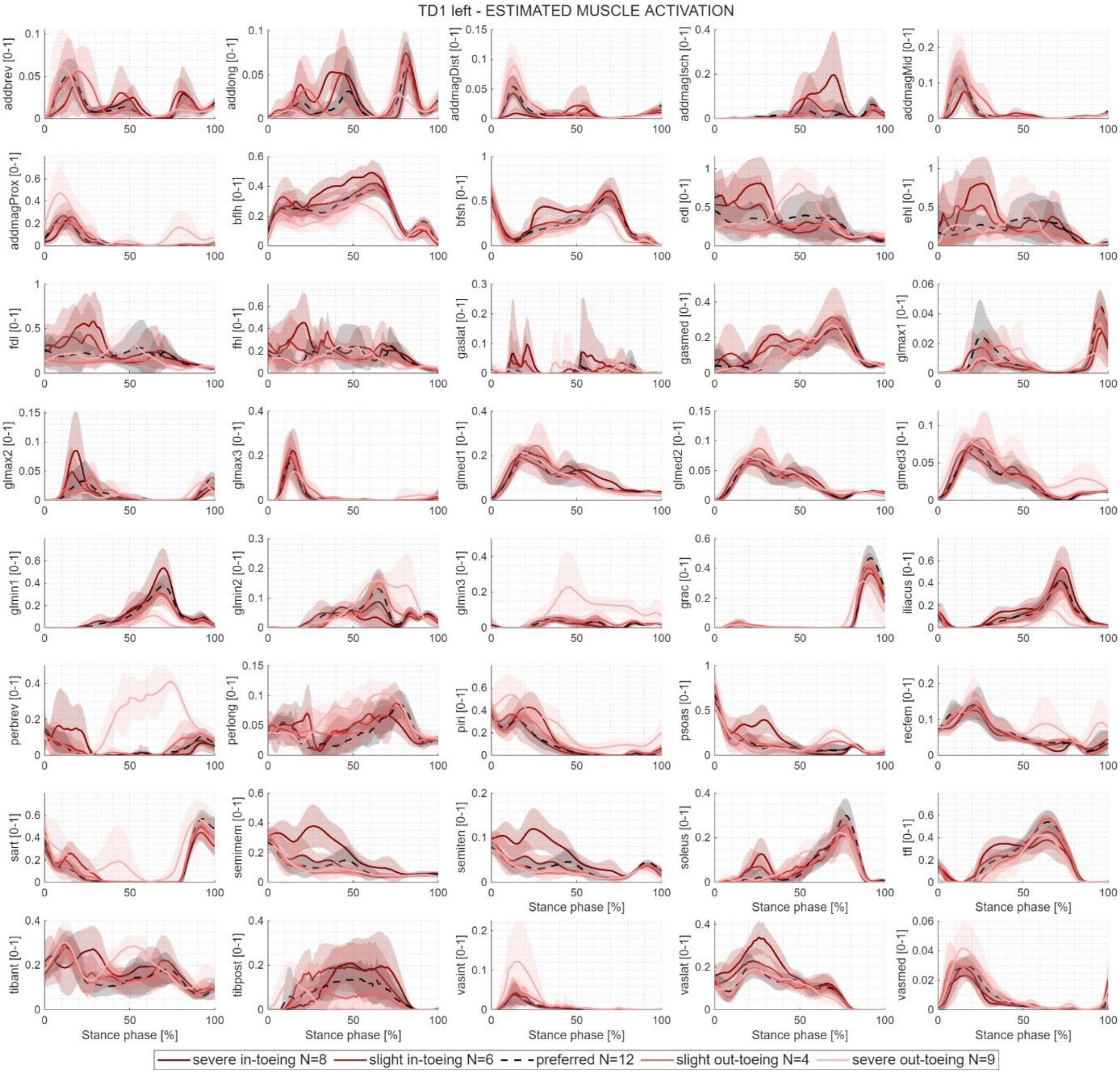

Figure S4: Mean and standard deviation (shaded) of estimated muscle activations obtained by EMG-informed simulations performed with the MuscleRedundancySolver.

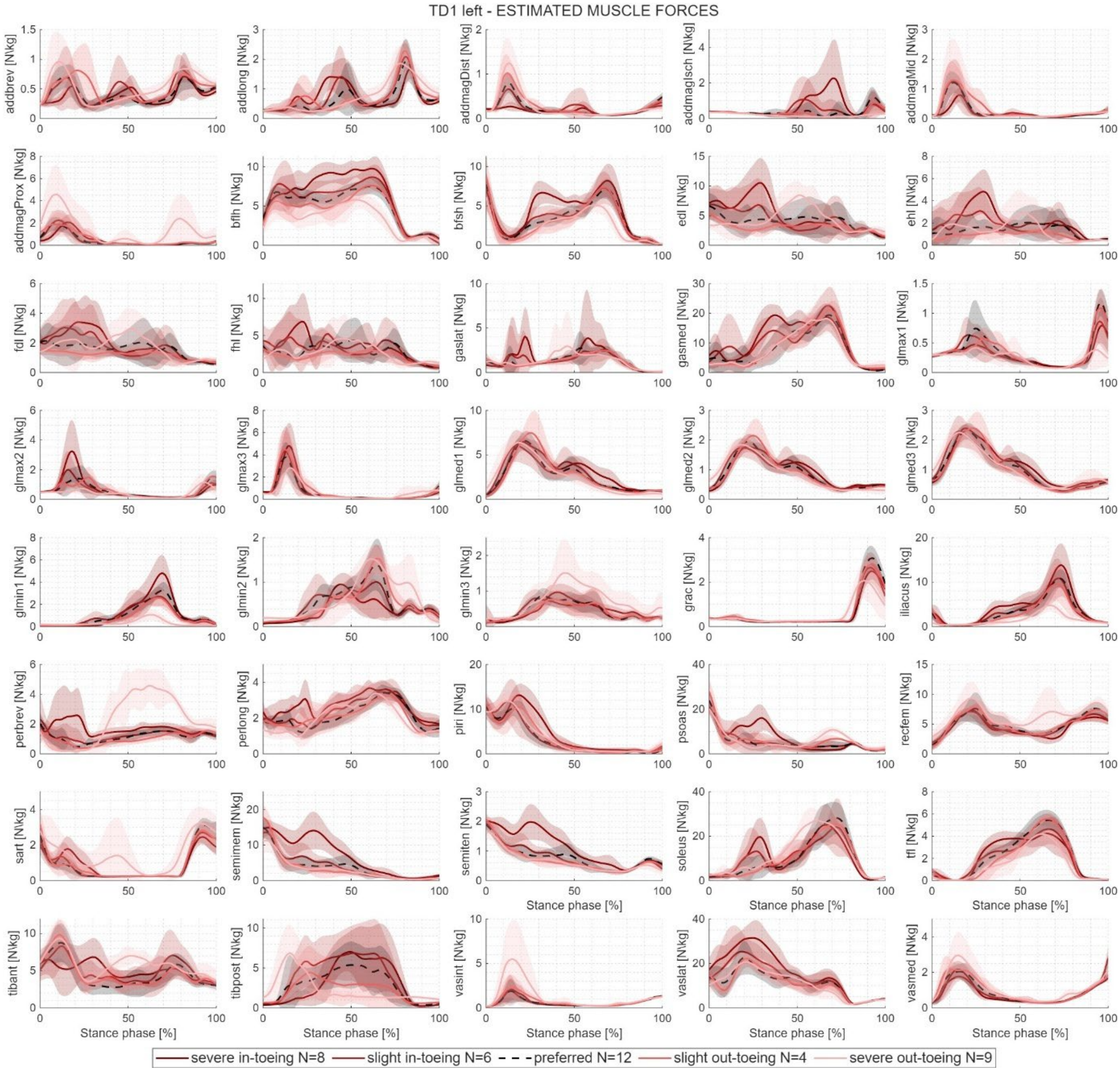

Figure S5: Mean and standard deviation (shaded) of estimated muscle forces obtained by EMG-informed simulations performed with the MuscleRedundancySolver.

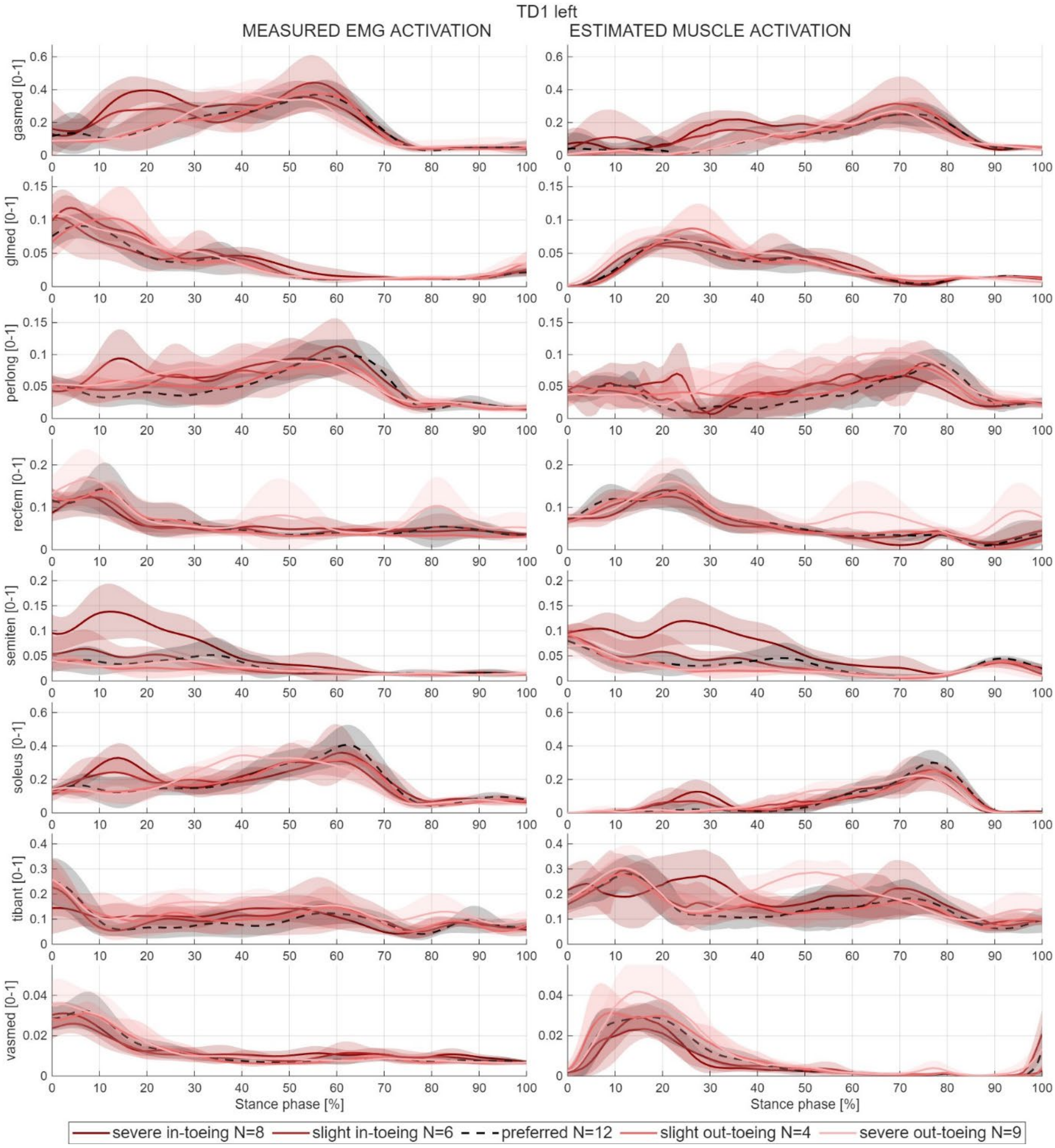

Figure S6: Mean and standard deviation (shaded) of measured muscle activation with electromyography (EMG) compared with the estimated muscle activation of the corresponding muscle in musculoskeletal simulations. Amplitude of EMG was scaled based on each muscle's maximum activation obtained from Static Optimization for the representative preferred gait trial.

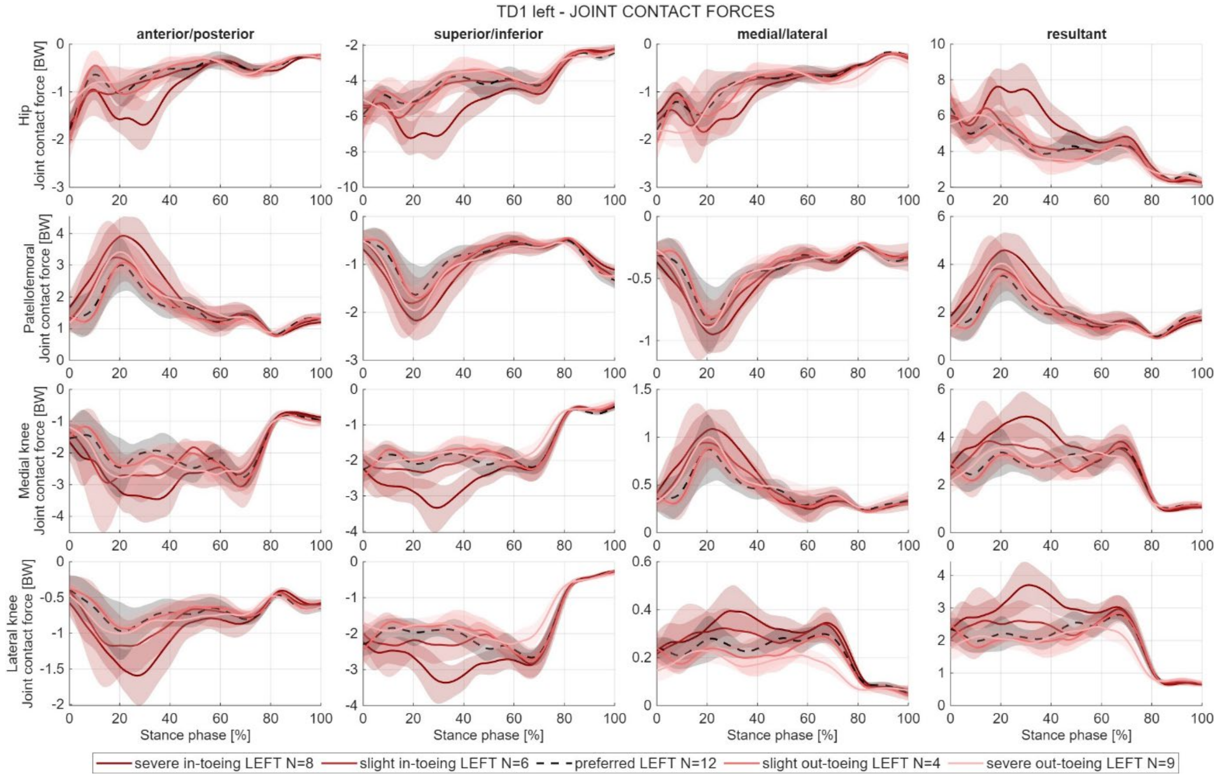

Figure S7: Mean and standard deviation (shaded) of hip, patellofemoral, medial and lateral knee joint contact forces obtained with OpenSim's Joint Reaction Analysis.

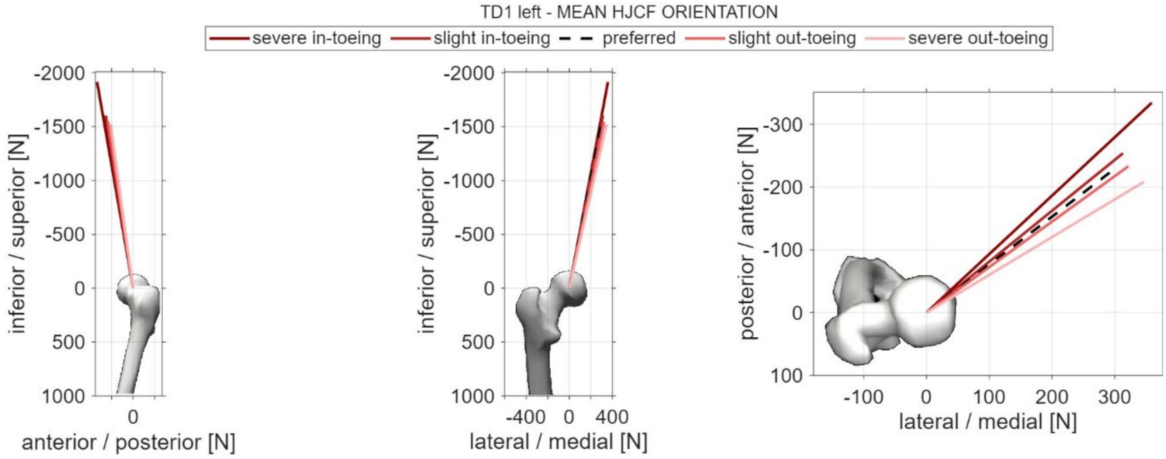

Figure S8:Visualization of the orientation vector of the mean resultant hip joint contact force (HJCF)

4 Detailed results for TD1 – right side

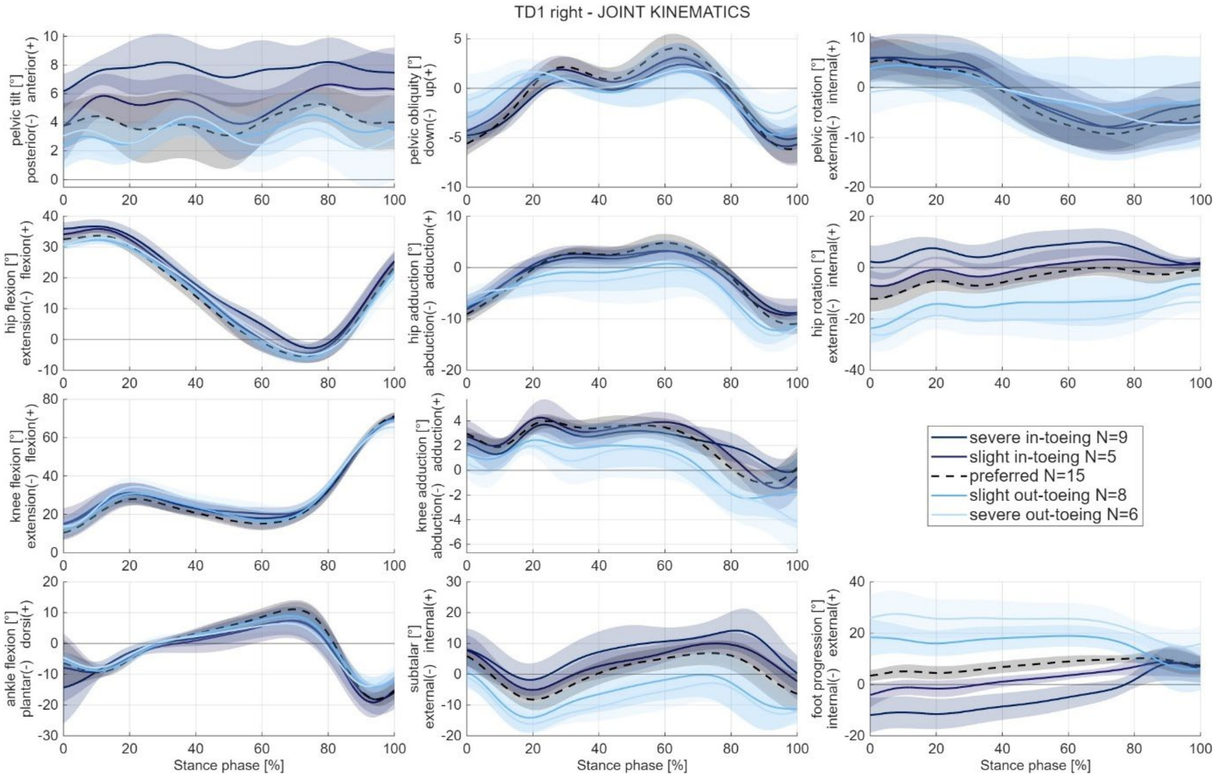

Figure S9: Mean and standard deviation (shaded) of joint angles quantified with OpenSim's Inverse Kinematics

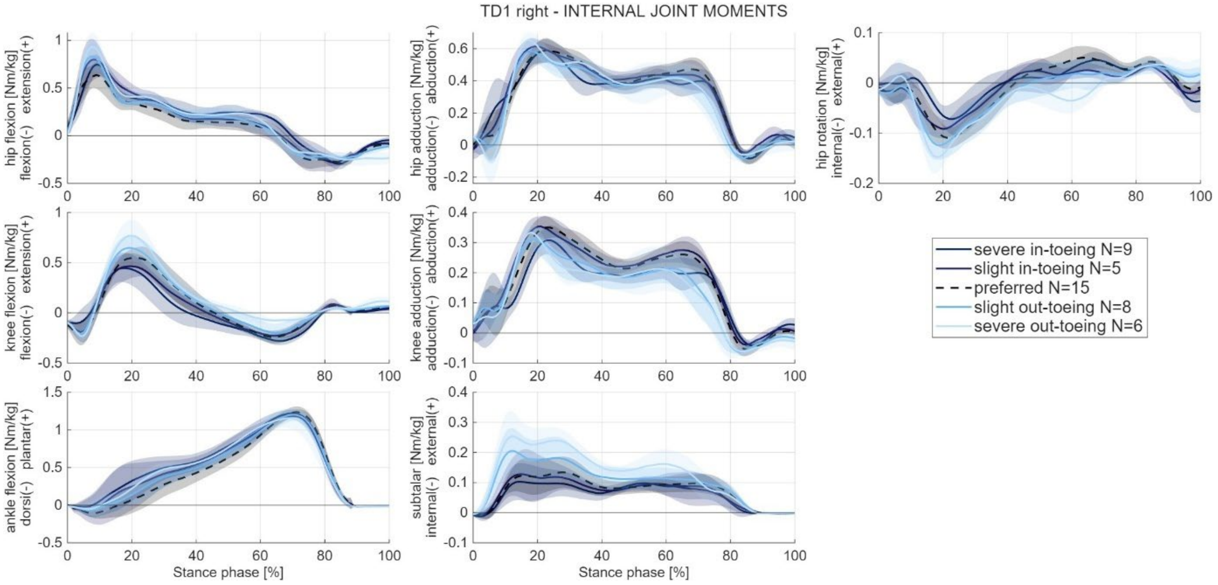

Figure S10: Mean and standard deviation (shaded) of internal joint moments quantified with OpenSim's Inverse Dynamics

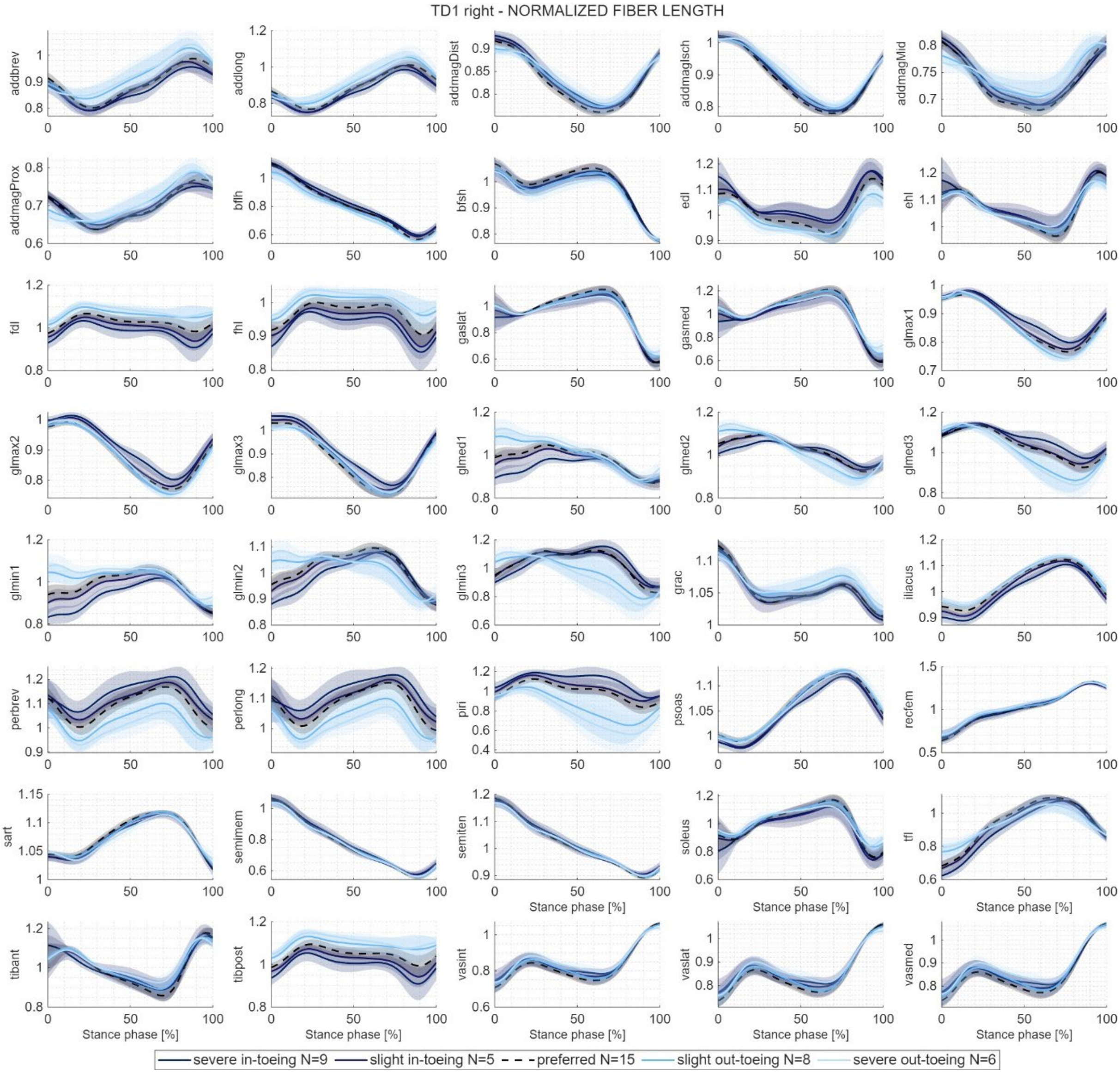

Figure S11: Mean and standard deviation (shaded) of normalized muscle fiber length quantified with OpenSim's Muscle Analysis

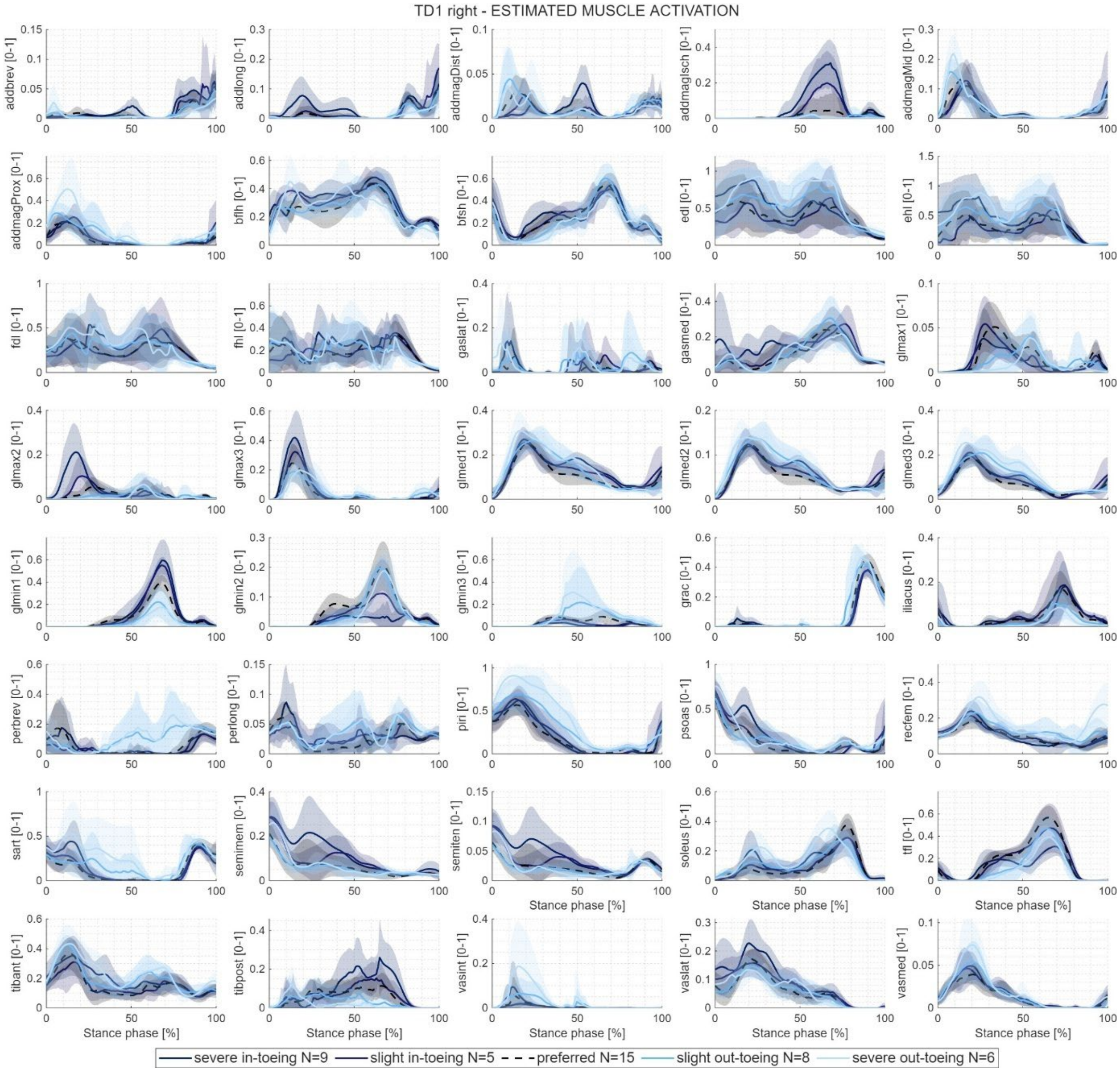

Figure S12: Mean and standard deviation (shaded) of estimated muscle activations obtained by EMG-informed simulations performed with the MuscleRedundancySolver.

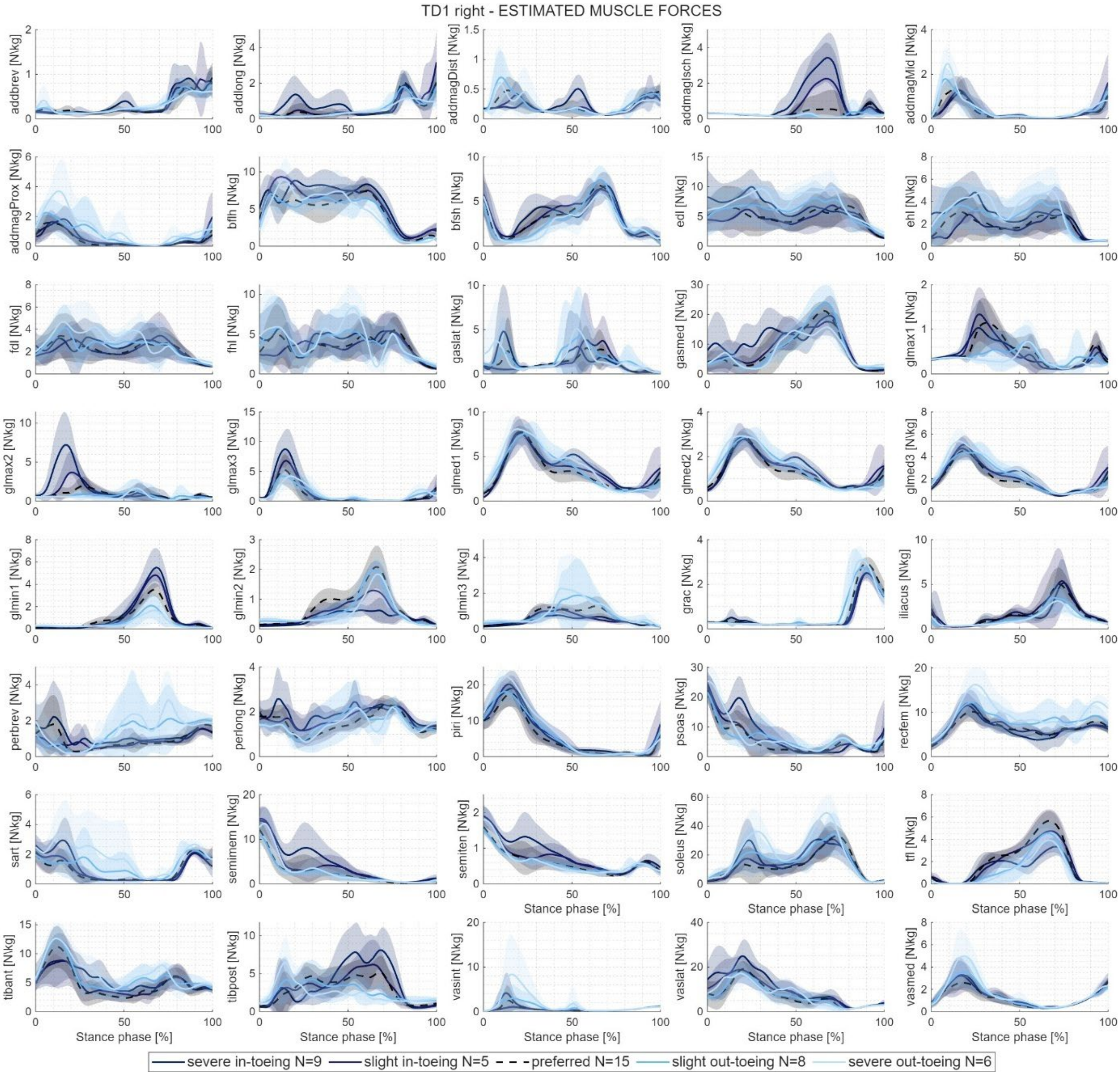

Figure S13: Mean and standard deviation (shaded) of estimated muscle forces obtained by EMG-informed simulations performed with the MuscleRedundancySolver.

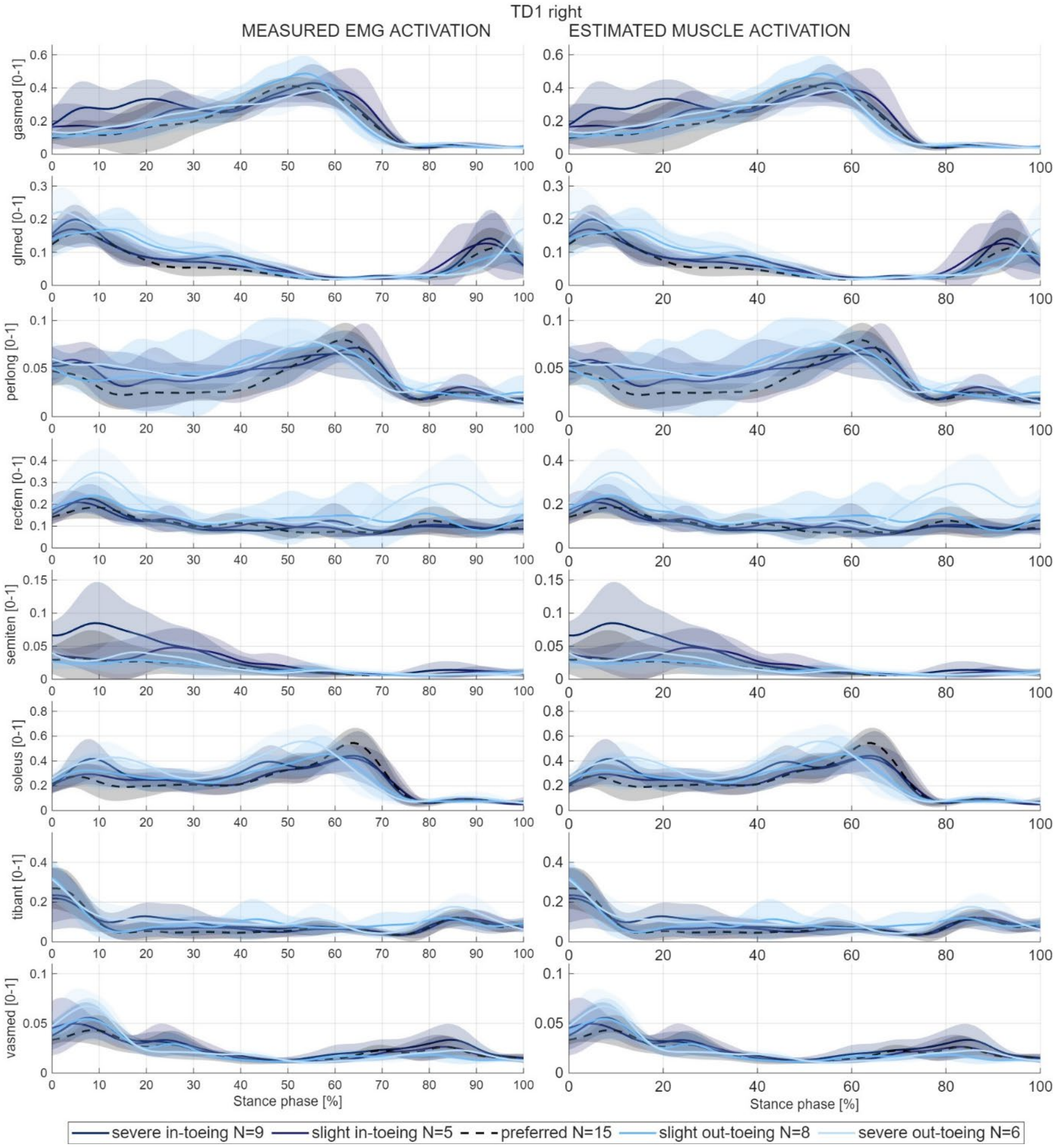

Figure S14: Mean and standard deviation (shaded) of measured muscle activation with electromyography (EMG) compared with the estimated muscle activation of the corresponding muscle in musculoskeletal simulations. Amplitude of EMG was scaled based on each muscle's maximum activation obtained from Static Optimization for the representative preferred gait trial.

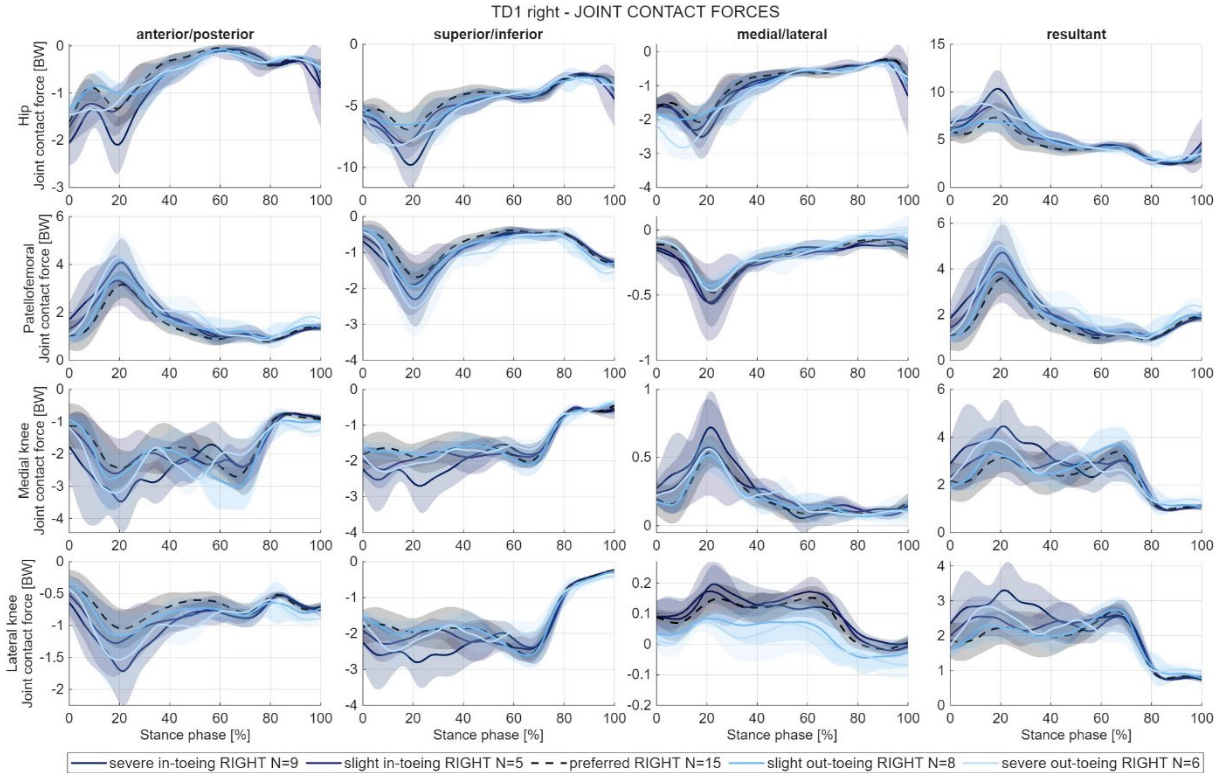

Figure S15: Mean and standard deviation (shaded) of hip, patellofemoral, medial and lateral knee joint contact forces obtained with OpenSim's Joint Reaction Analysis.

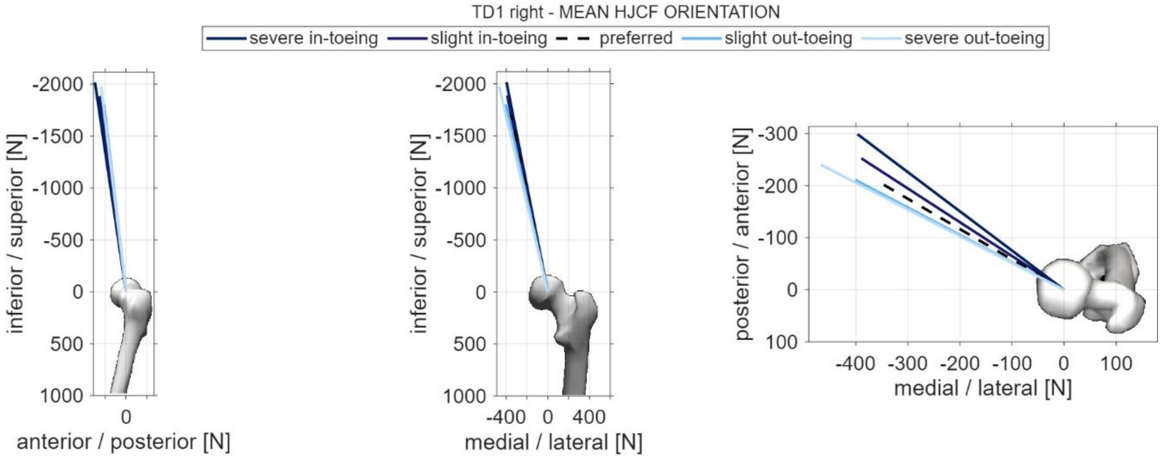

Figure S16:Visualization of the orientation vector of the mean resultant hip joint contact force (HJCF)

5 Detailed results for TD2 – left side

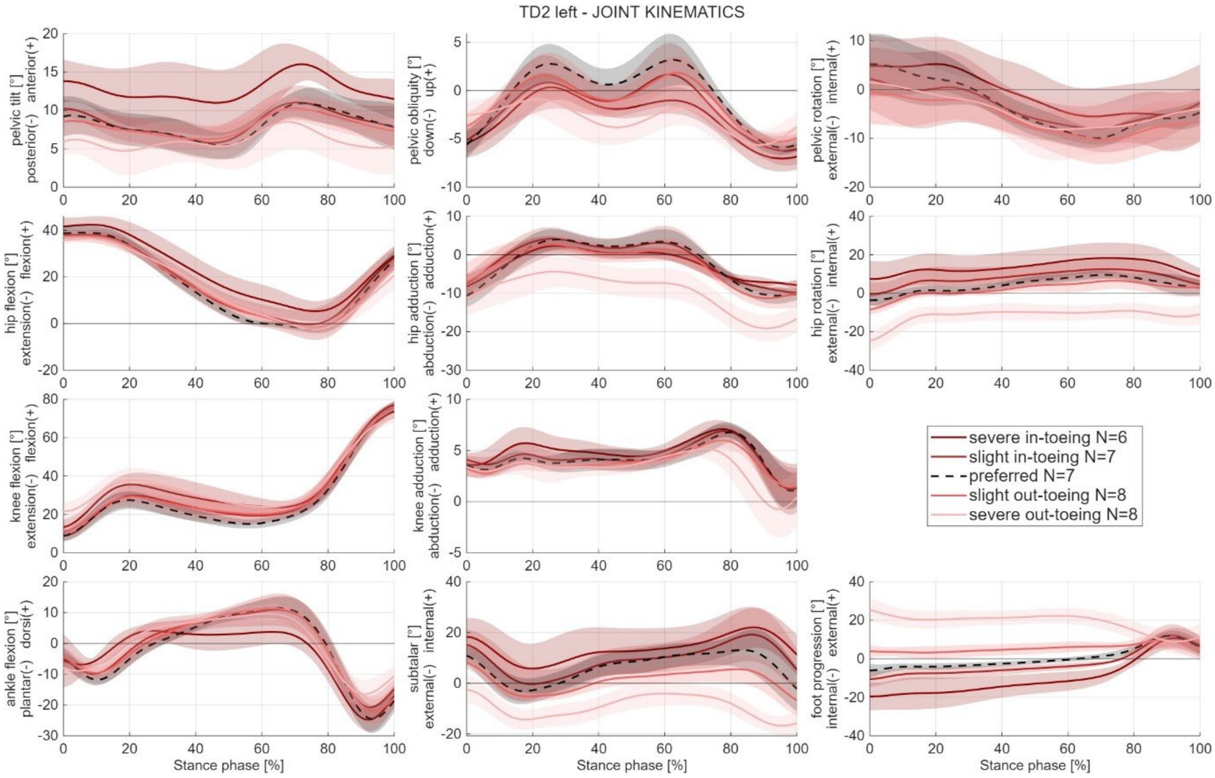

Figure S17: Mean and standard deviation (shaded) of joint angles quantified with OpenSim's Inverse Kinematics

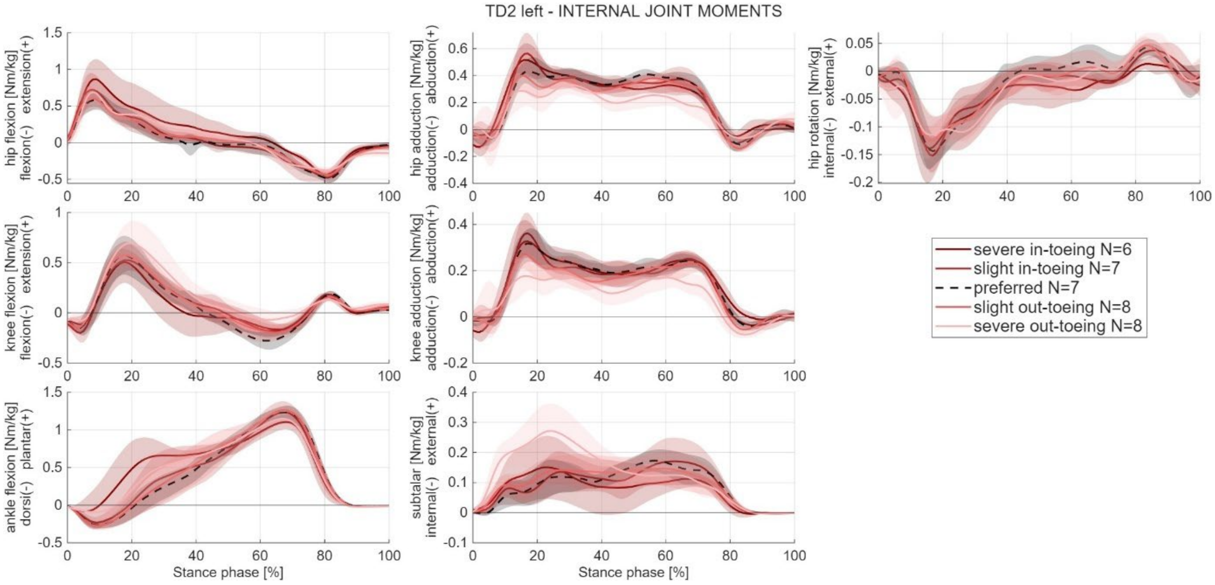

Figure S18: Mean and standard deviation (shaded) of internal joint moments quantified with OpenSim's Inverse Dynamics

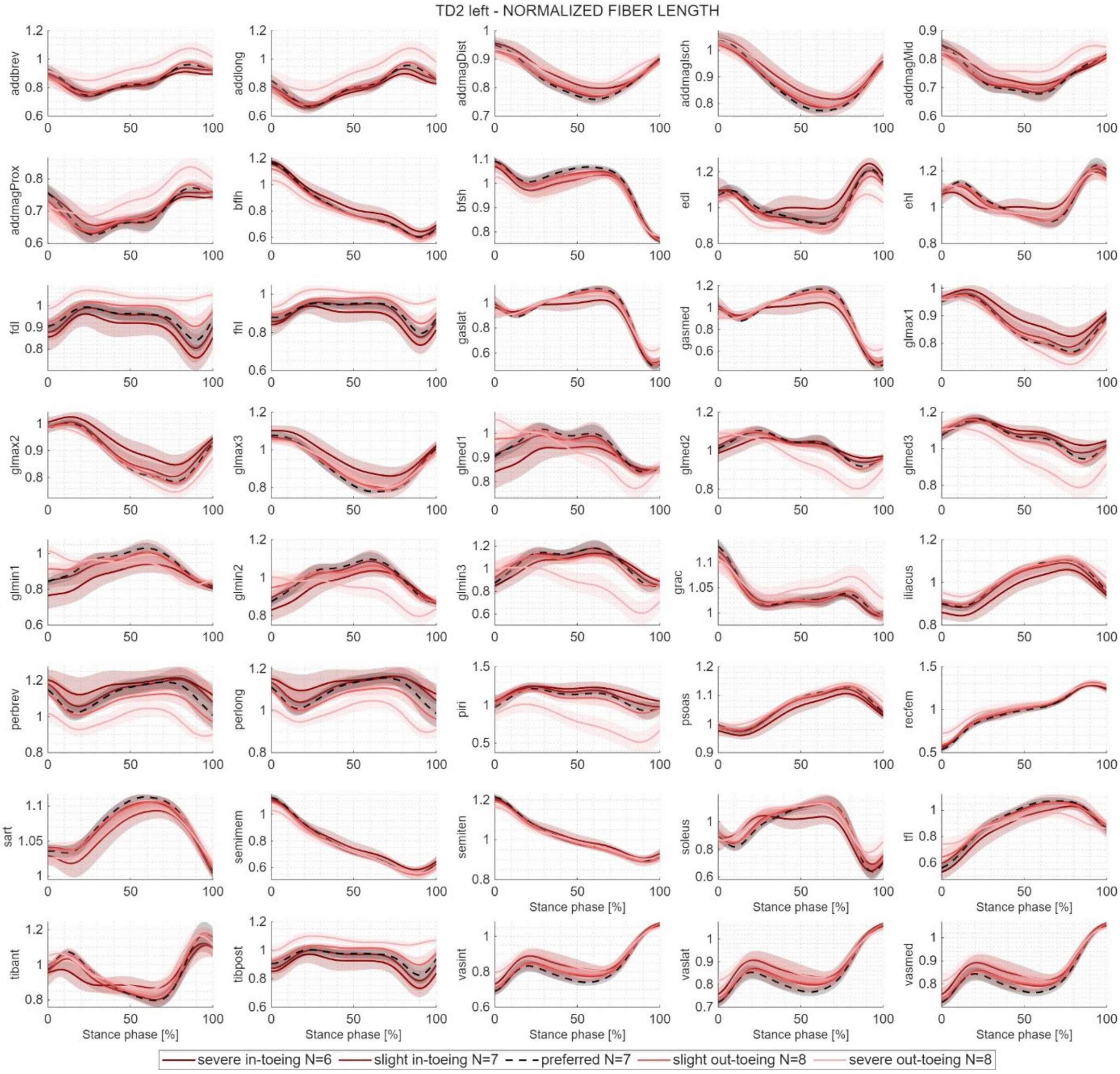

Figure S19: Mean and standard deviation (shaded) of normalized muscle fiber length quantified with OpenSim's Muscle Analysis

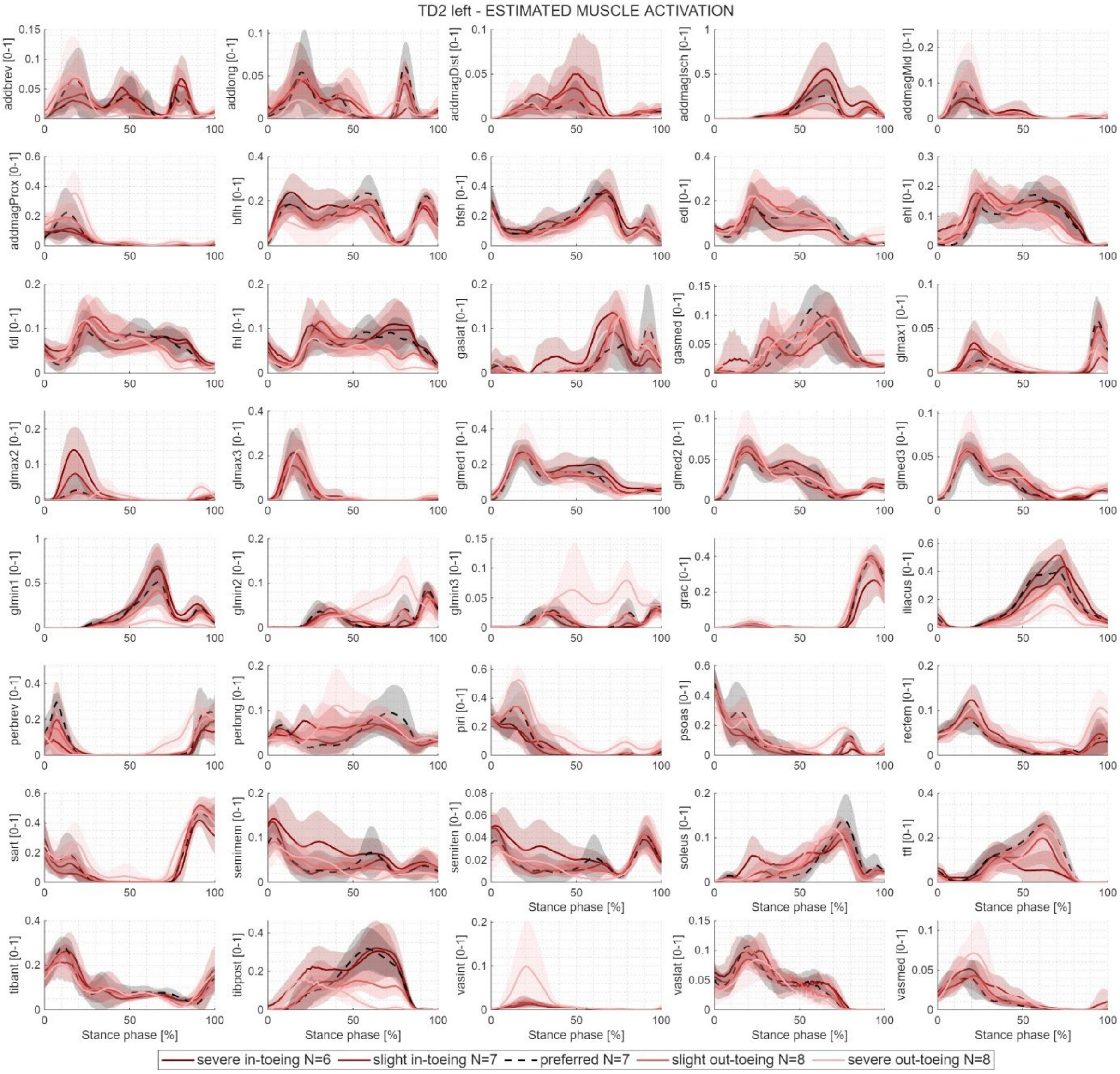

Figure S20: Mean and standard deviation (shaded) of estimated muscle activations obtained by EMG-informed simulations performed with the MuscleRedundancySolver.

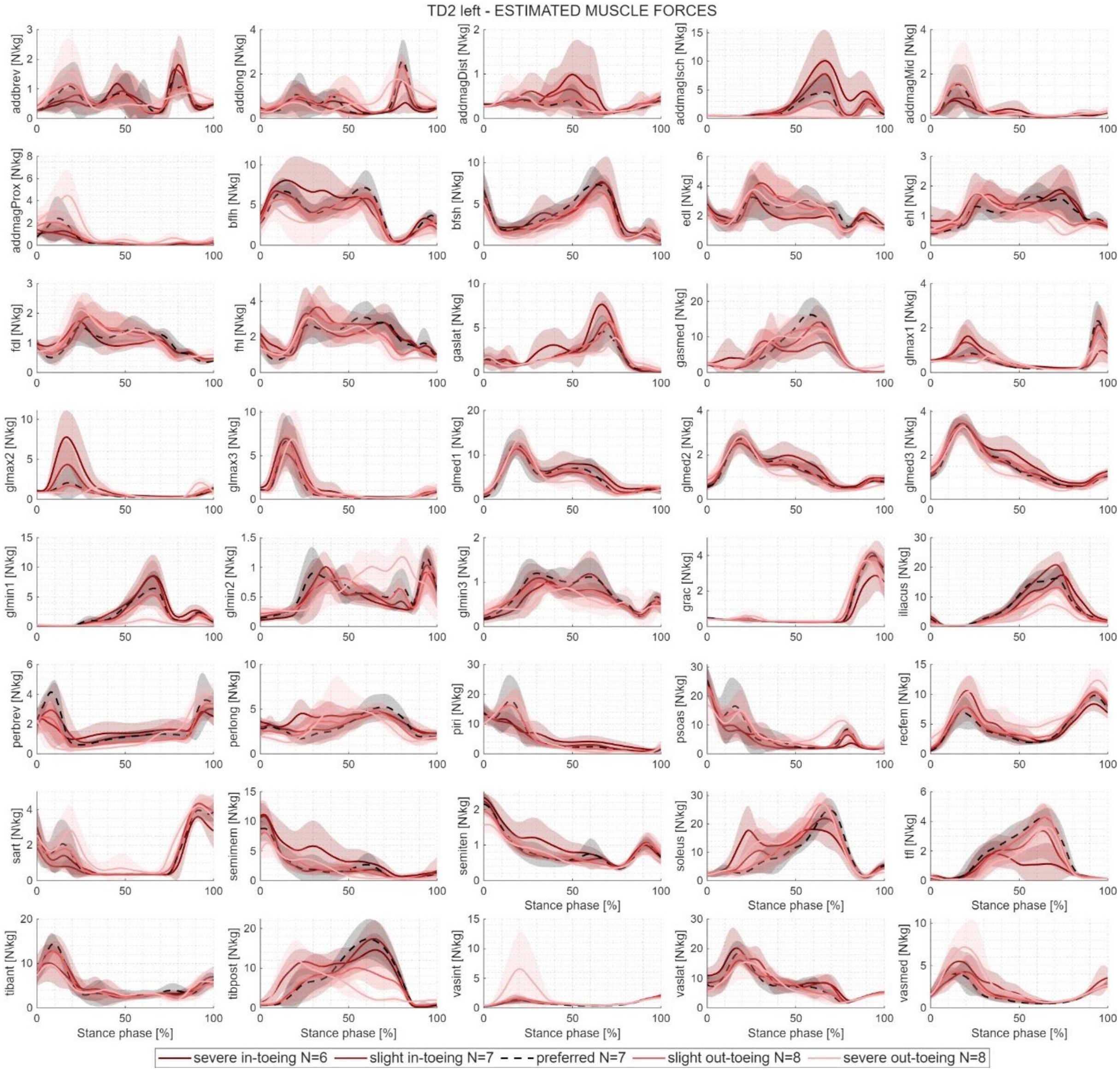

Figure S21: Mean and standard deviation (shaded) of estimated muscle forces obtained by EMG-informed simulations performed with the MuscleRedundancySolver.

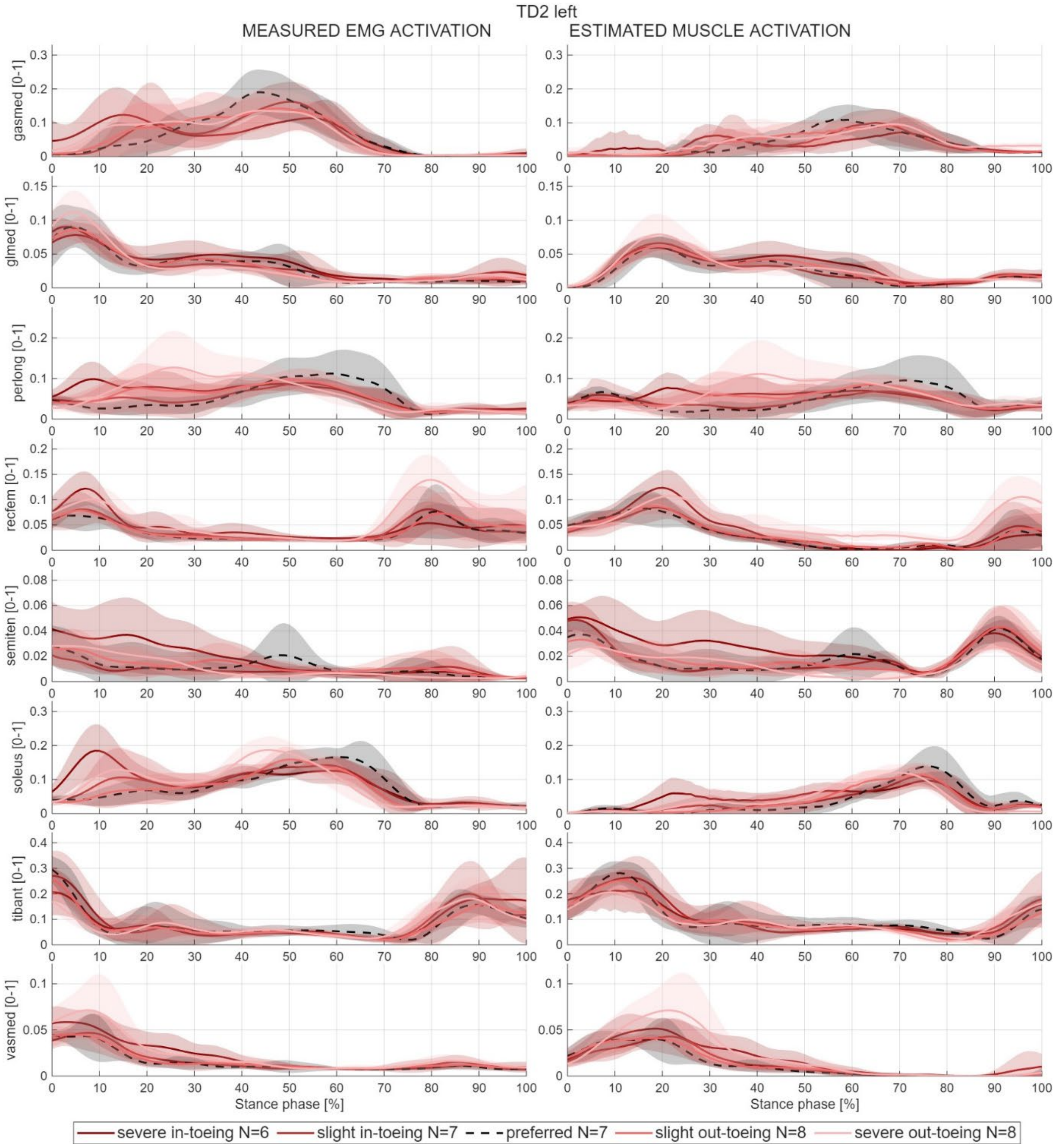

Figure S22: Mean and standard deviation (shaded) of measured muscle activation with electromyography (EMG) compared with the estimated muscle activation of the corresponding muscle in musculoskeletal simulations. Amplitude of EMG was scaled based on each muscle's maximum activation obtained from Static Optimization for the representative preferred gait trial.

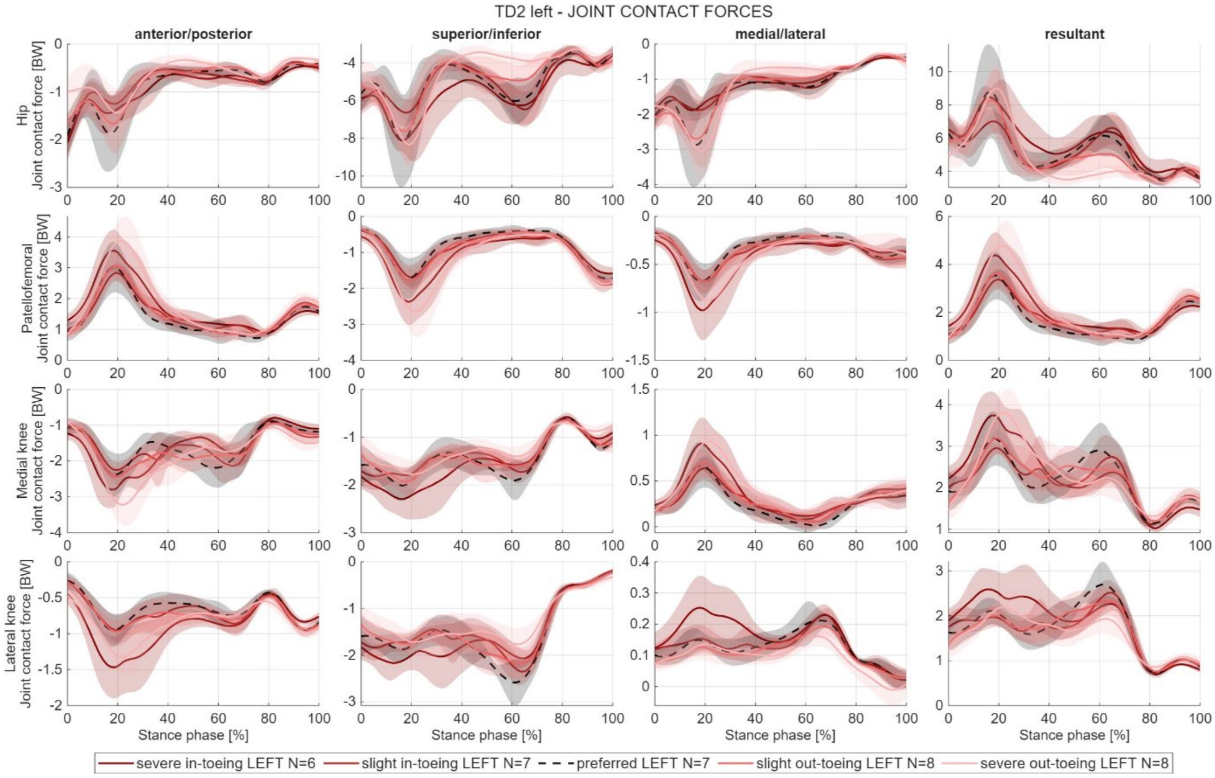

Figure S23: Mean and standard deviation (shaded) of hip, patellofemoral, medial and lateral knee joint contact forces obtained with OpenSim's Joint Reaction Analysis.

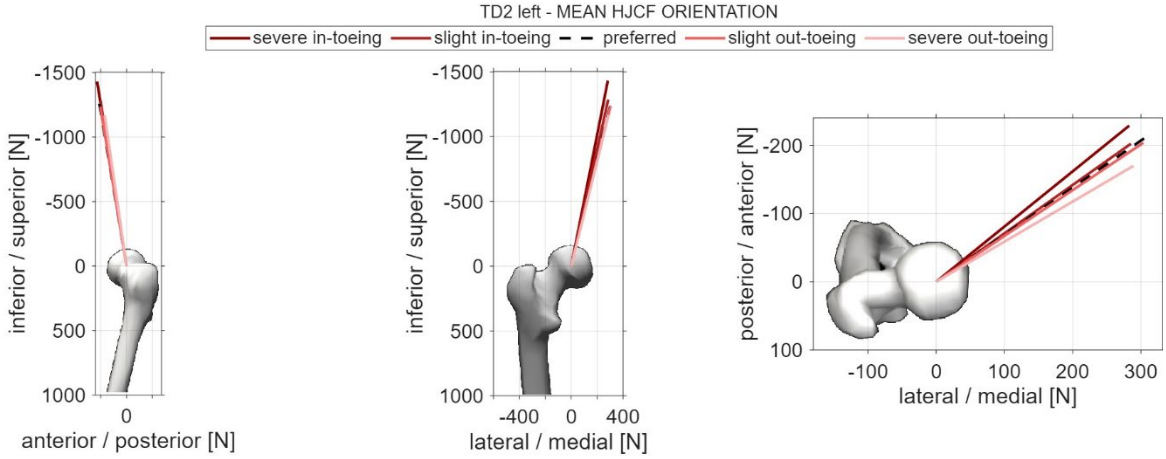

Figure S24:Visualization of the orientation vector of the mean resultant hip joint contact force (HJCF)

6 Detailed results for TD2 – right side

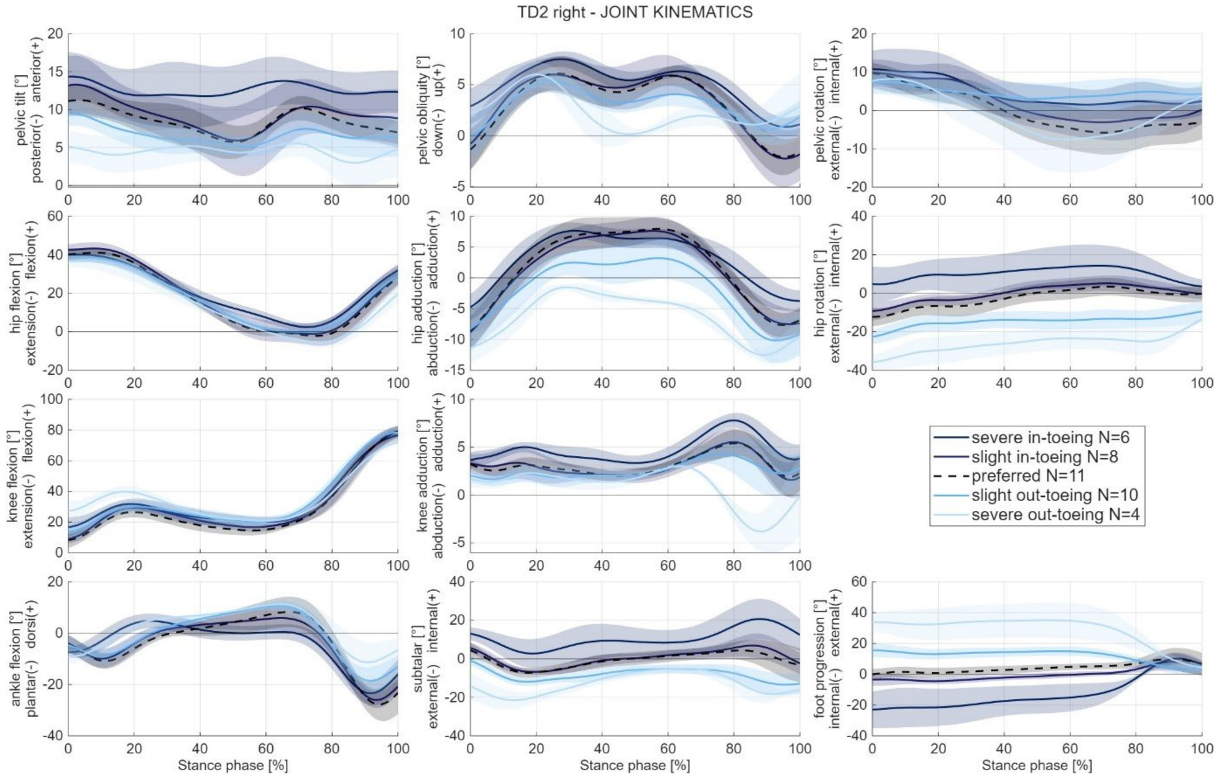

Figure S25: Mean and standard deviation (shaded) of joint angles quantified with OpenSim's Inverse Kinematics

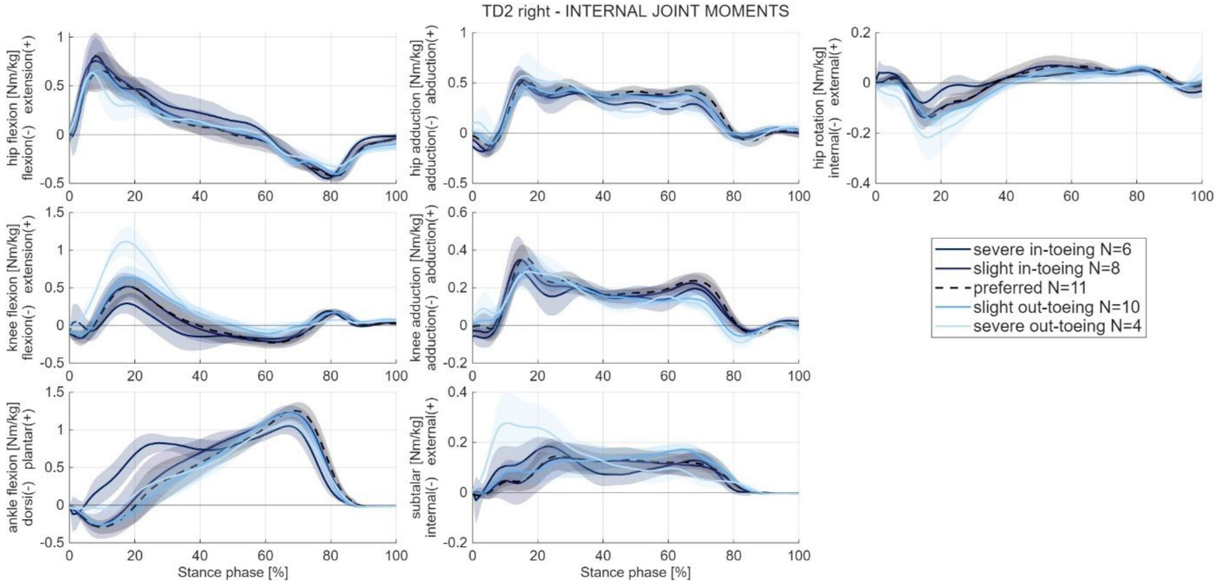

Figure S26: Mean and standard deviation (shaded) of internal joint moments quantified with OpenSim's Inverse Dynamic

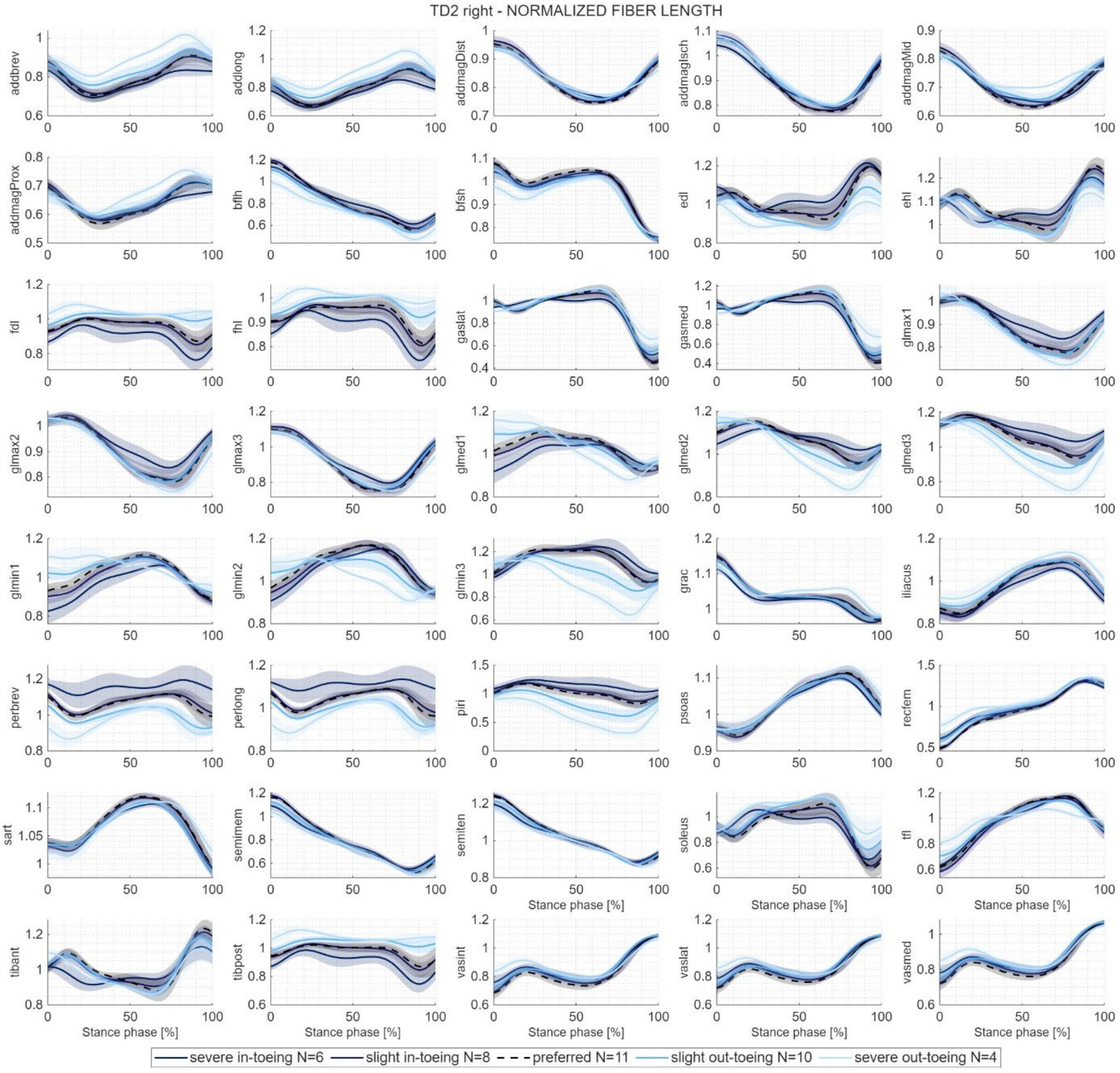

Figure S27: Mean and standard deviation (shaded) of normalized muscle fiber length quantified with OpenSim's Muscle Analysis

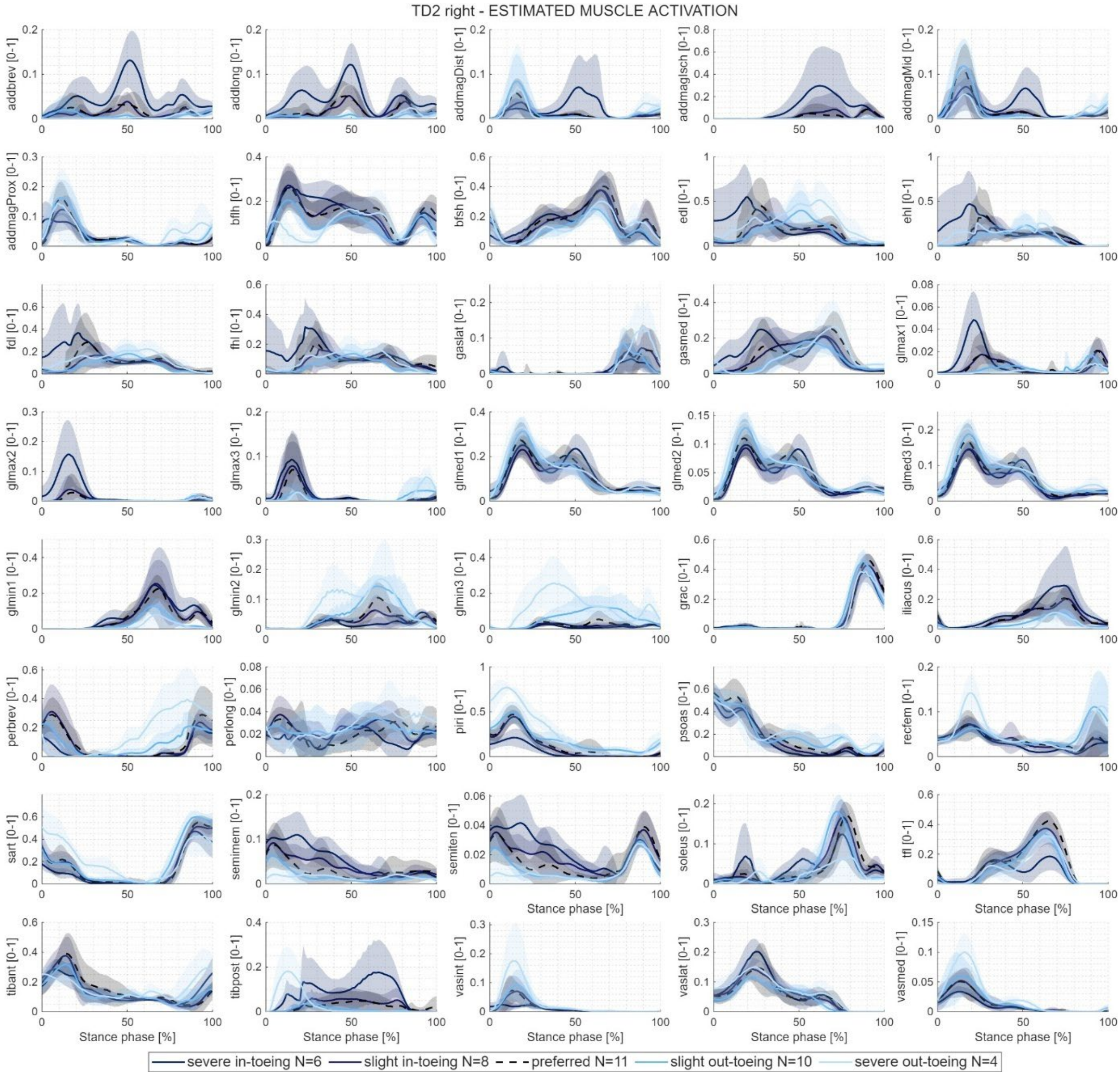

Figure S28: Mean and standard deviation (shaded) of estimated muscle activations obtained by EMG-informed simulations performed with the MuscleRedundancySolver.

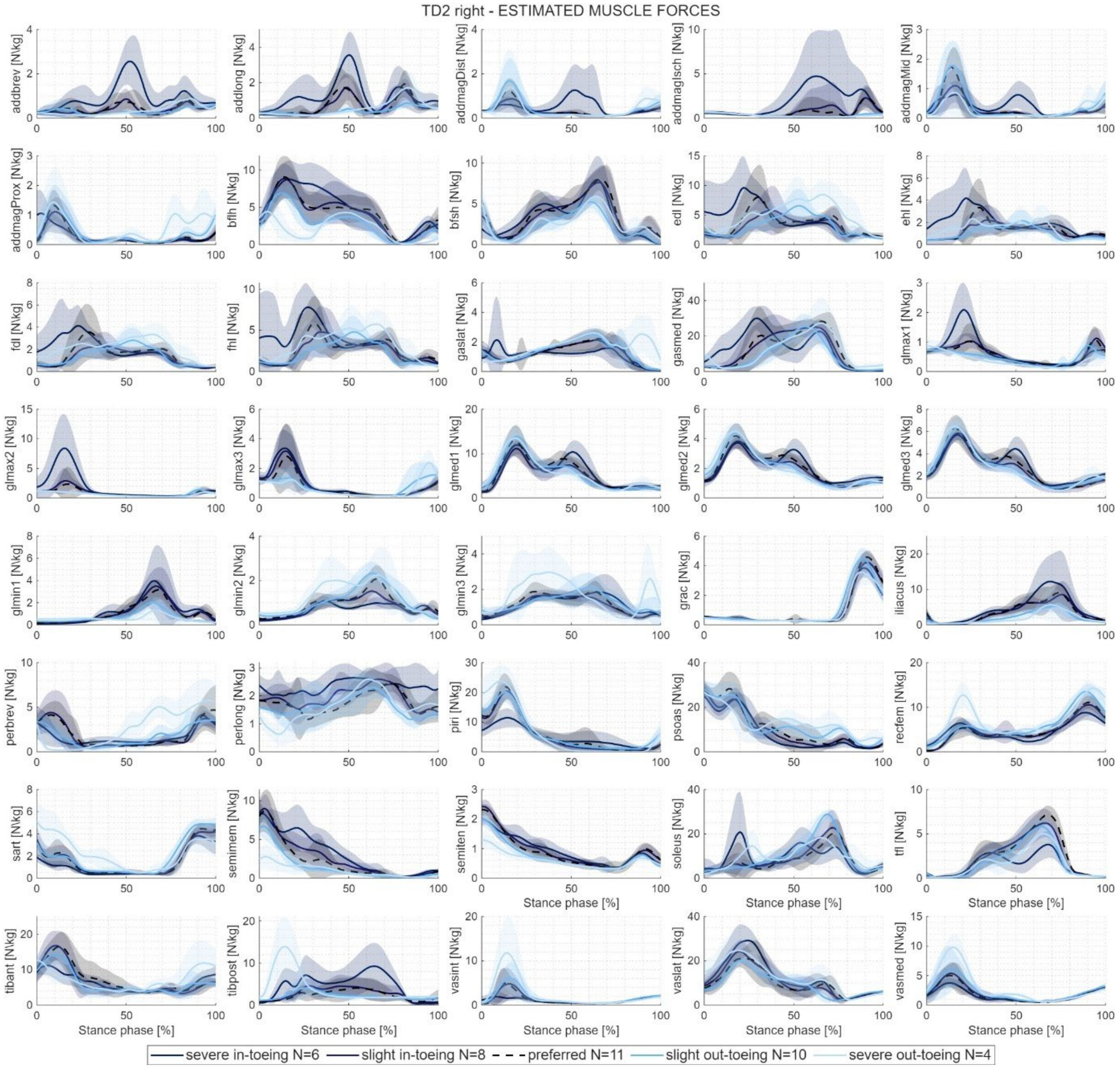

Figure S29: Mean and standard deviation (shaded) of estimated muscle forces obtained by EMG-informed simulations performed with the MuscleRedundancySolver.

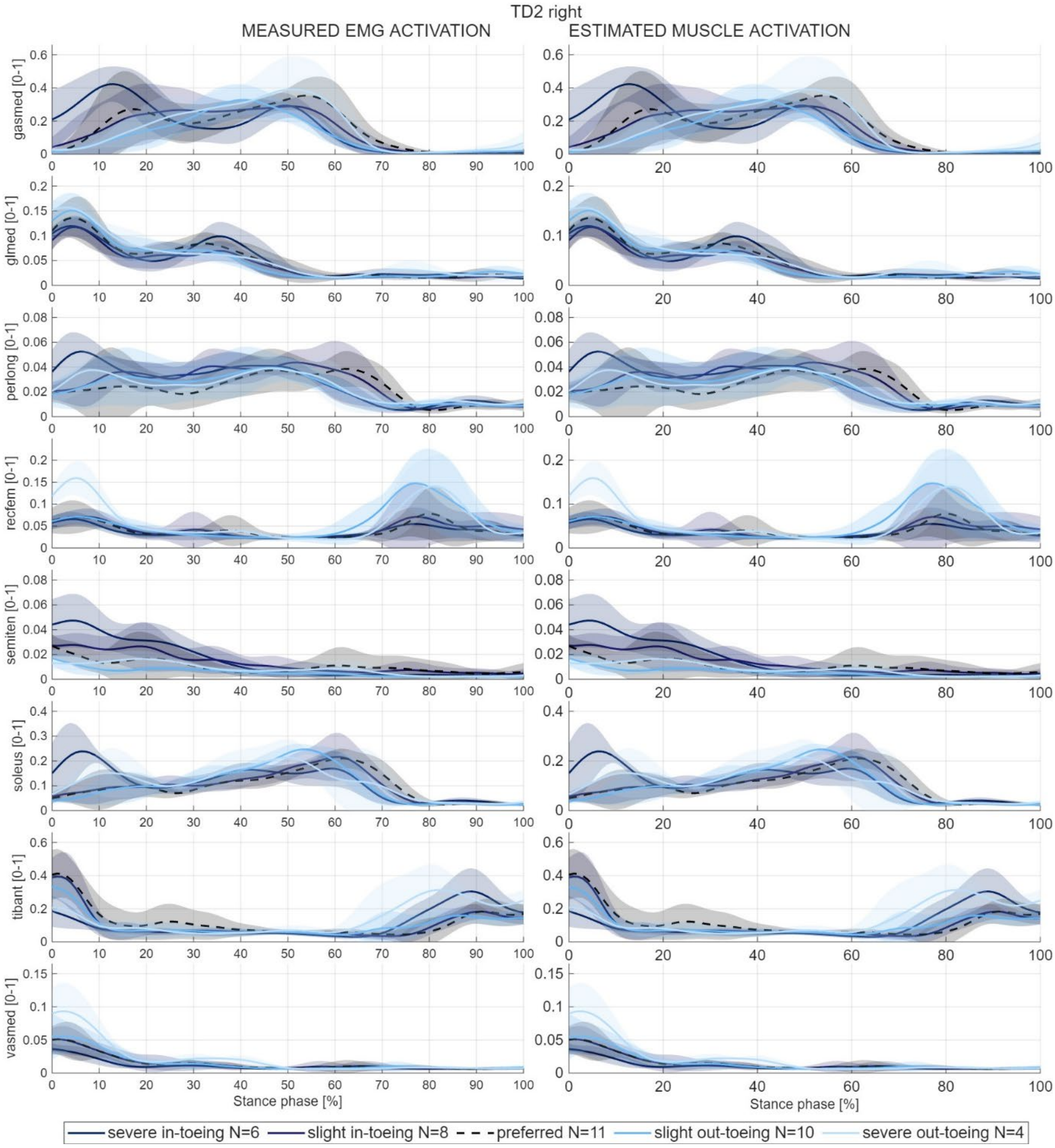

Figure S30: Mean and standard deviation (shaded) of measured muscle activation with electromyography (EMG) compared with the estimated muscle activation of the corresponding muscle in musculoskeletal simulations. Amplitude of EMG was scaled based on each muscle's maximum activation obtained from Static Optimization for the representative preferred gait trial.

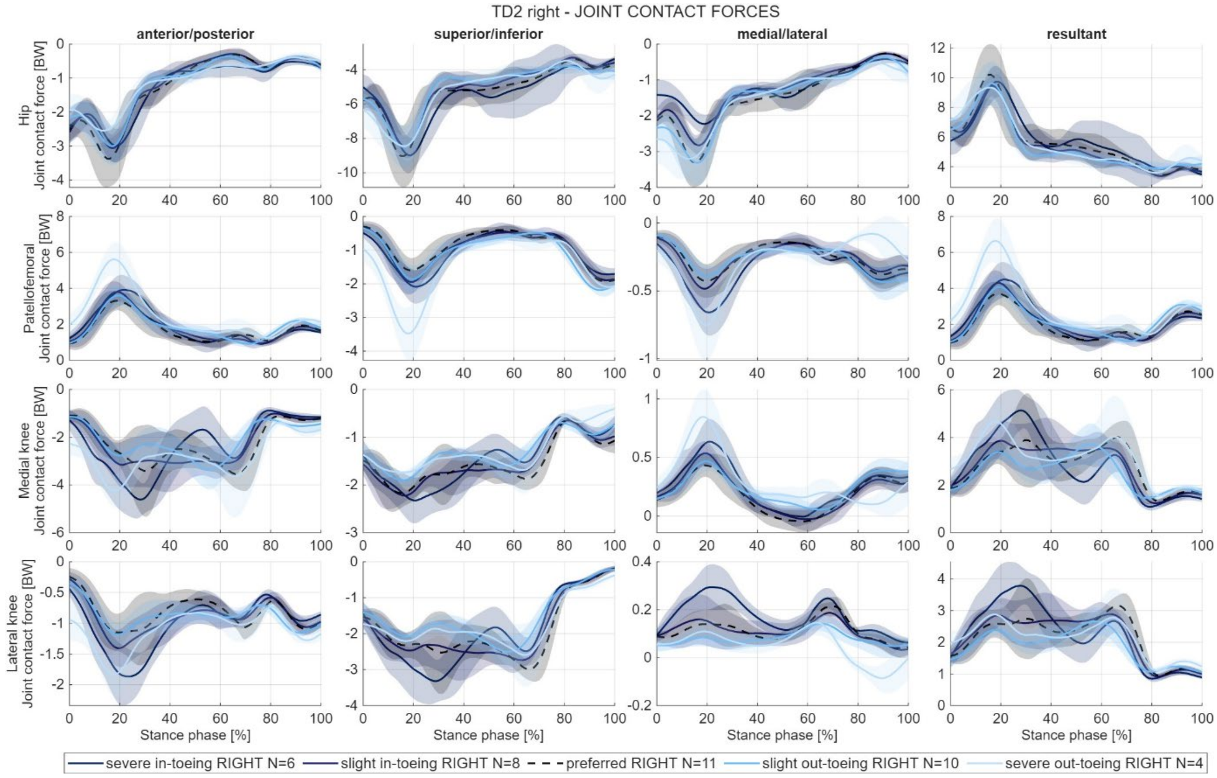

Figure S31: Mean and standard deviation (shaded) of hip, patellofemoral, medial and lateral knee joint contact forces obtained with OpenSim's Joint Reaction Analysis.

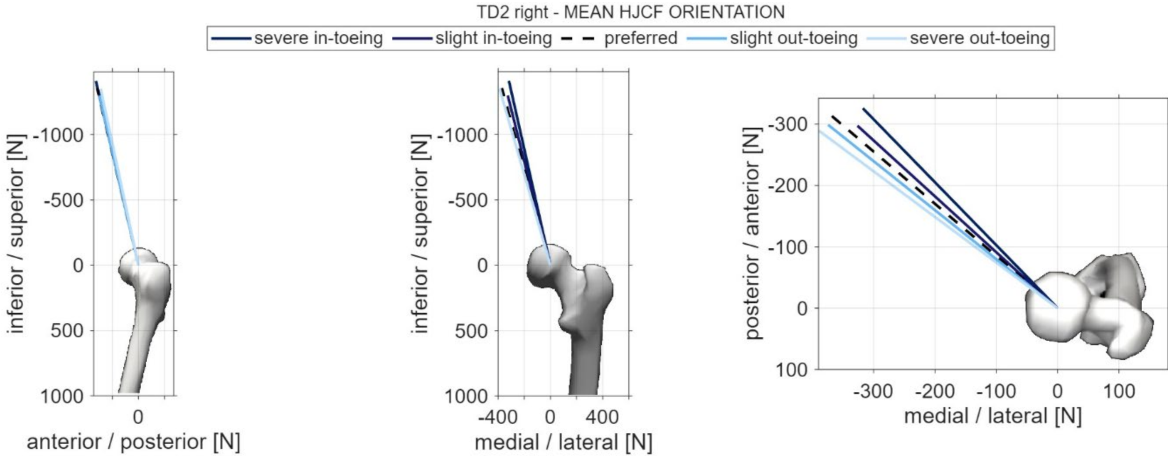

Figure S32:Visualization of the orientation vector of the mean resultant hip joint contact force (HJCF)

7 Detailed results for TD3 – left side

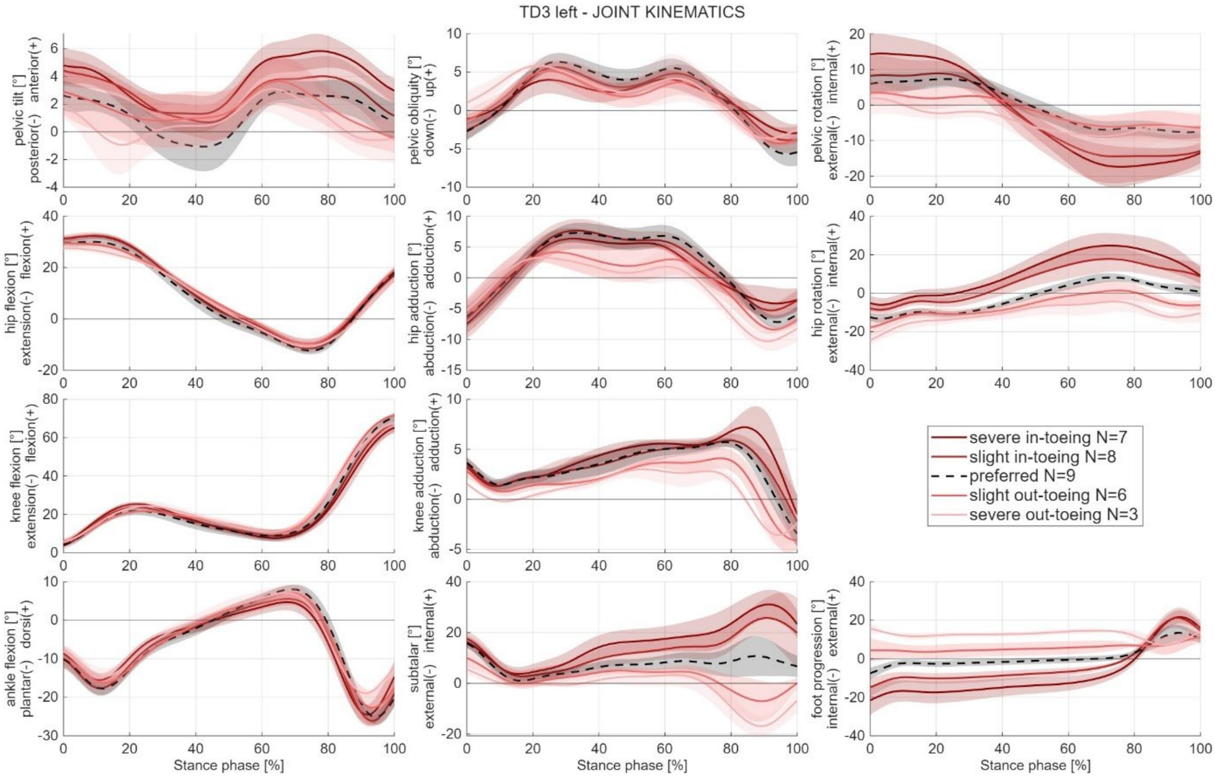

Figure S33: Mean and standard deviation (shaded) of joint angles quantified with OpenSim's Inverse Kinematics

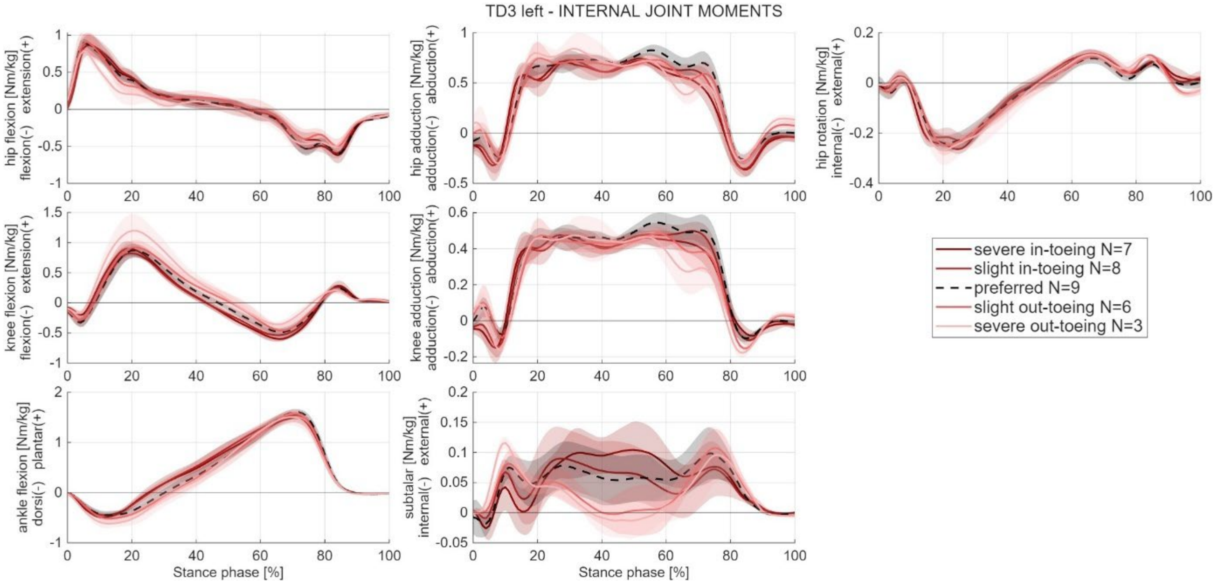

Figure S34: Mean and standard deviation (shaded) of internal joint moments quantified with OpenSim's Inverse Dynamics

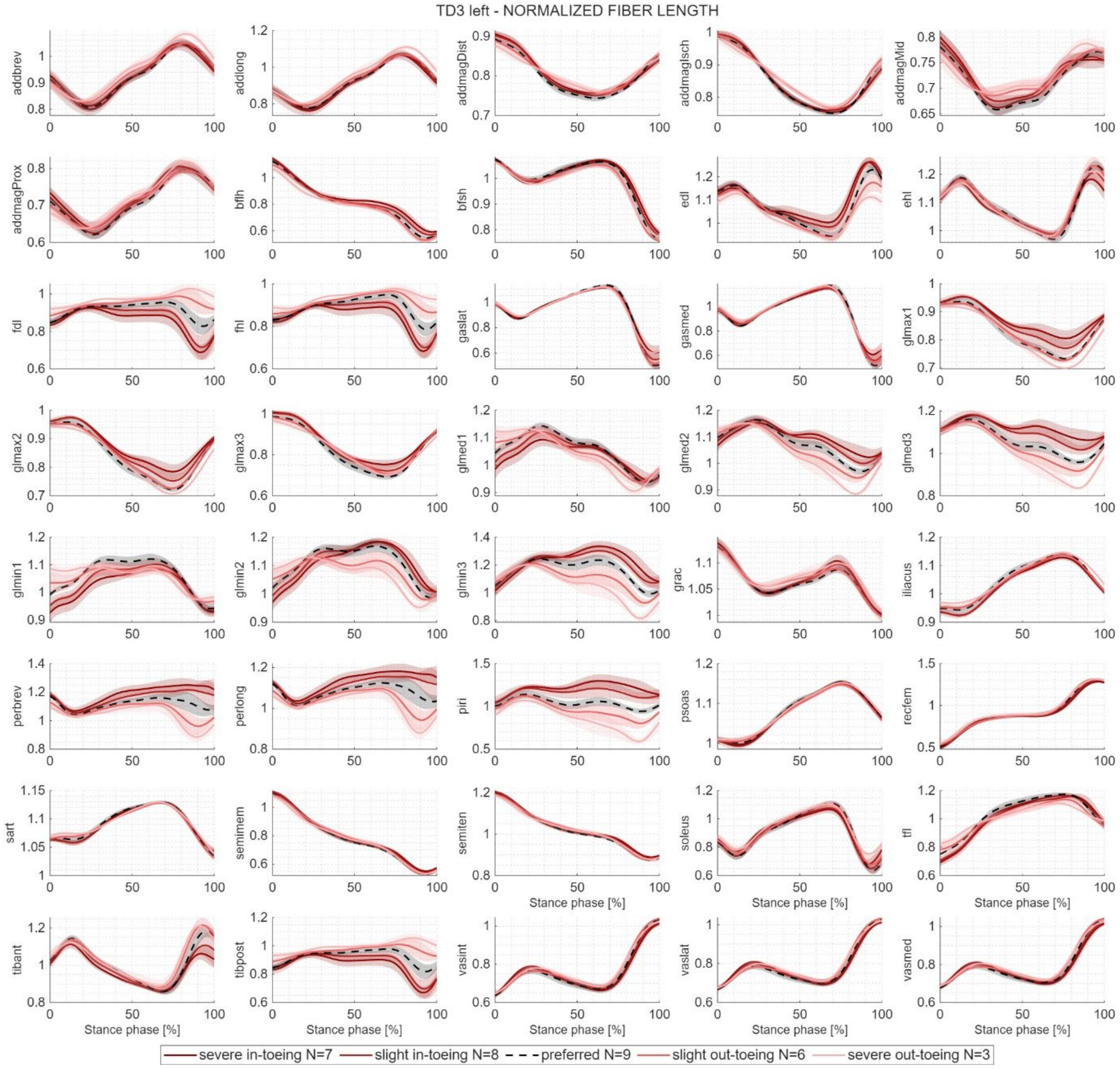

Figure S35: Mean and standard deviation (shaded) of normalized muscle fiber length quantified with OpenSim's Muscle Analysis

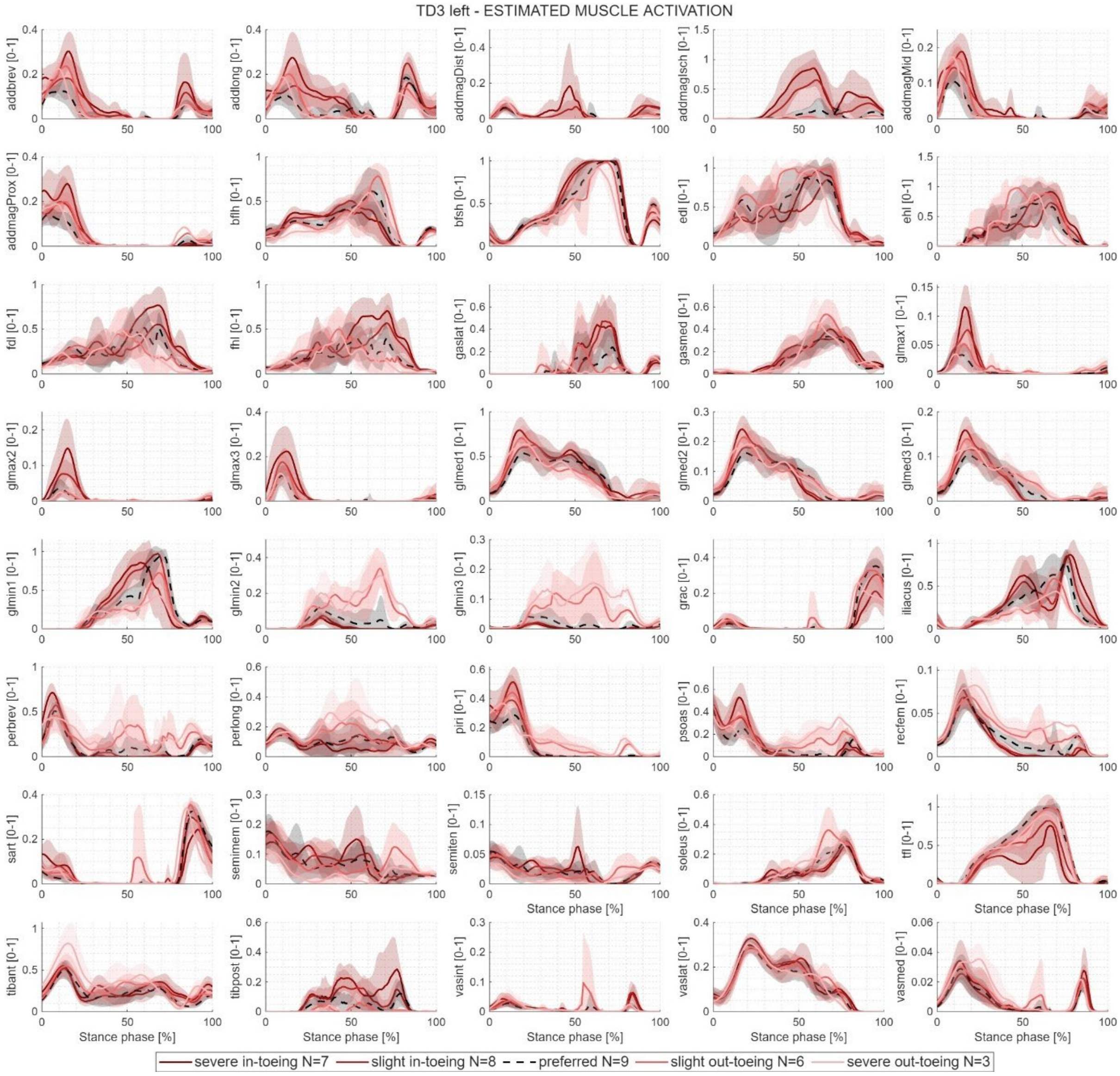

Figure S36: Mean and standard deviation (shaded) of estimated muscle activations obtained by EMG-informed simulations performed with the MuscleRedundancySolver.

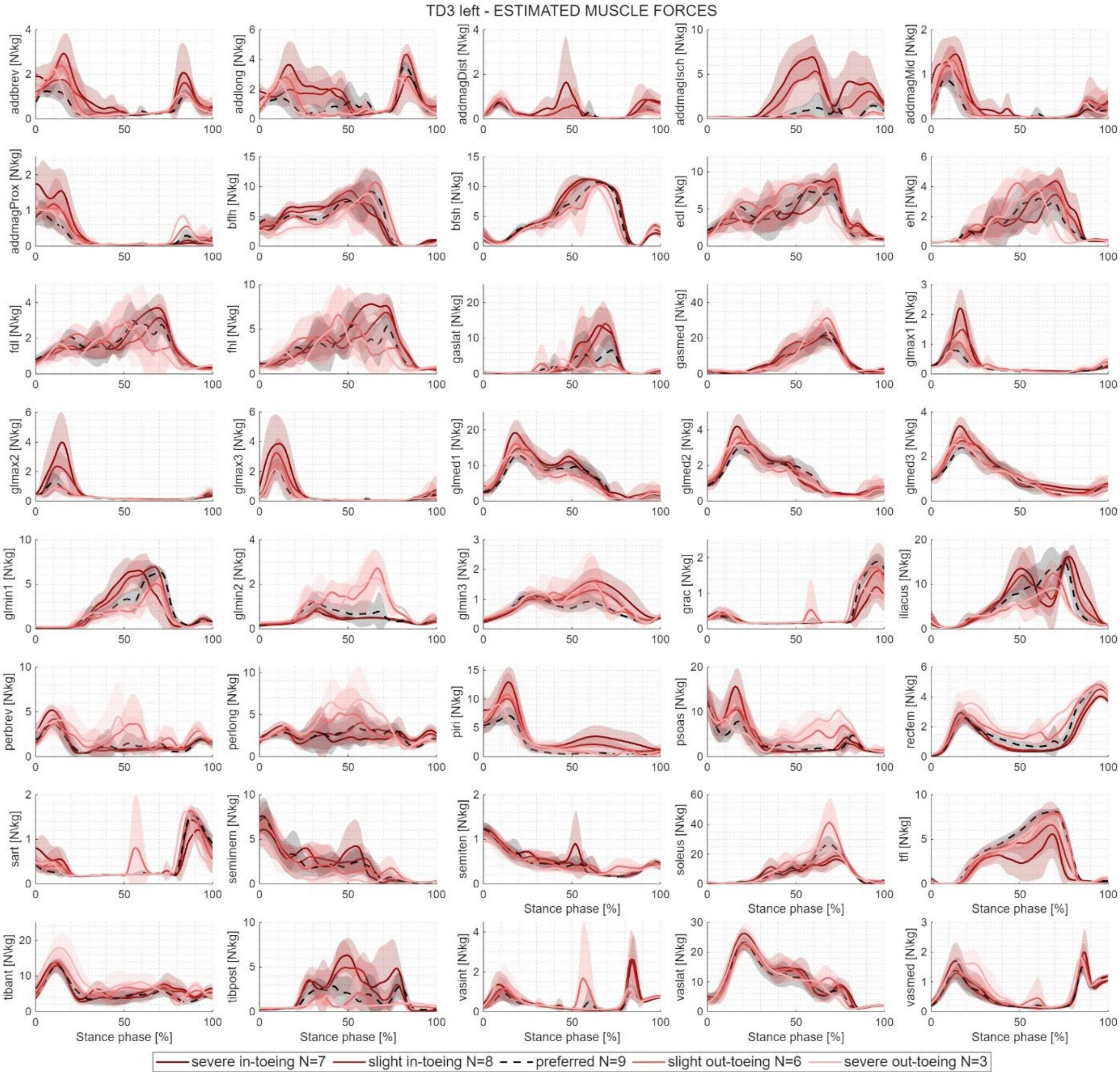

Figure S37: Mean and standard deviation (shaded) of estimated muscle forces obtained by EMG-informed simulations performed with the MuscleRedundancySolver.

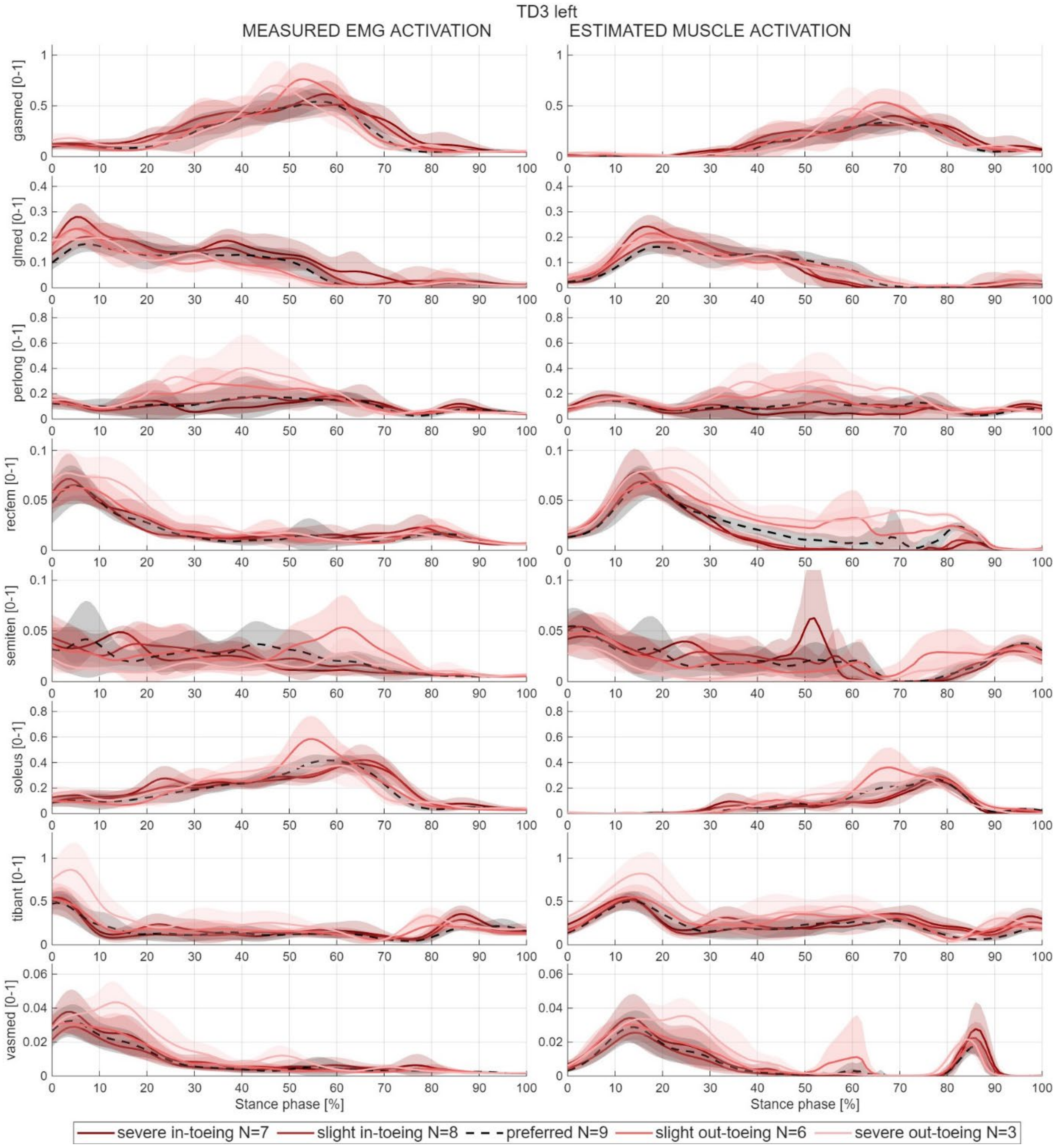

Figure S38: Mean and standard deviation (shaded) of measured muscle activation with electromyography (EMG) compared with the estimated muscle activation of the corresponding muscle in musculoskeletal simulations. Amplitude of EMG was scaled based on each muscle's maximum activation obtained from Static Optimization for the representative preferred gait trial.

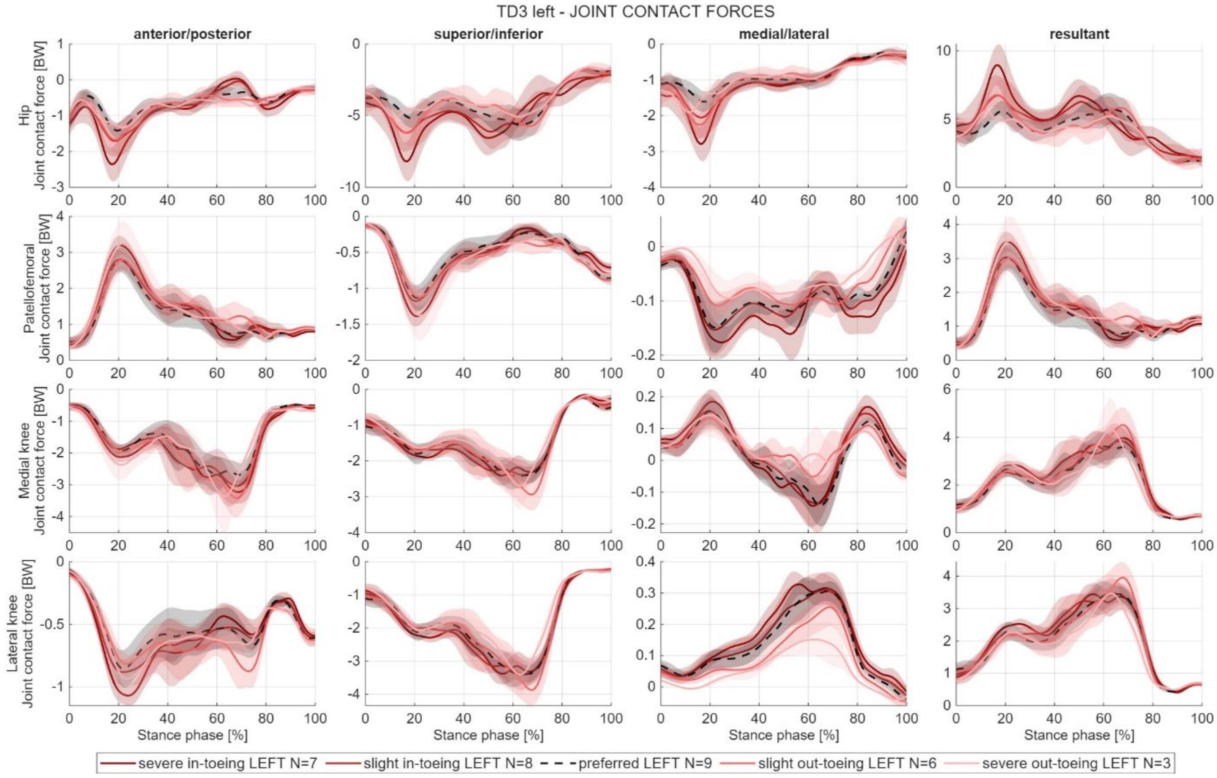

Figure S39: Mean and standard deviation (shaded) of hip, patellofemoral, medial and lateral knee joint contact forces obtained with OpenSim's Joint Reaction Analysis.

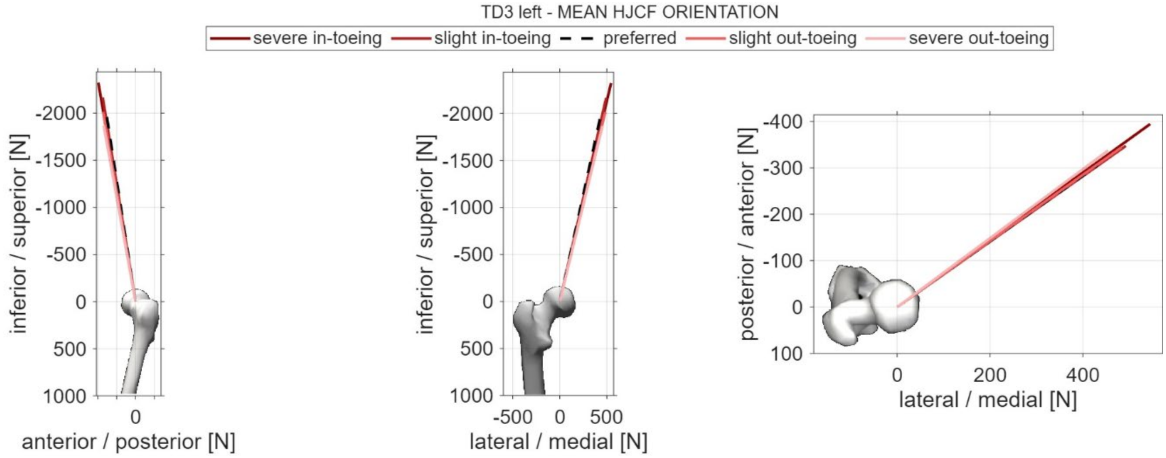

Figure S40:Visualization of the orientation vector of the mean resultant hip joint contact force (HJCF)

8 Detailed results for TD3 – right side

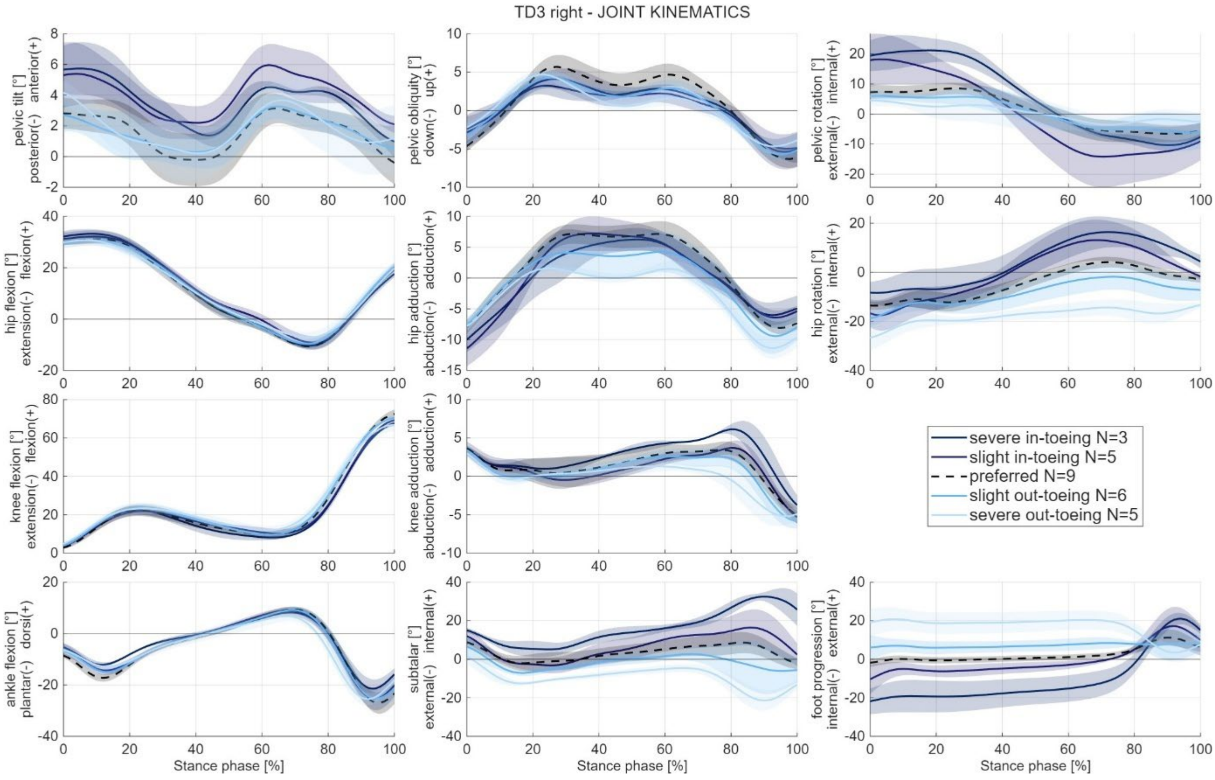

Figure S41: Mean and standard deviation (shaded) of joint angles quantified with OpenSim's Inverse Kinematics

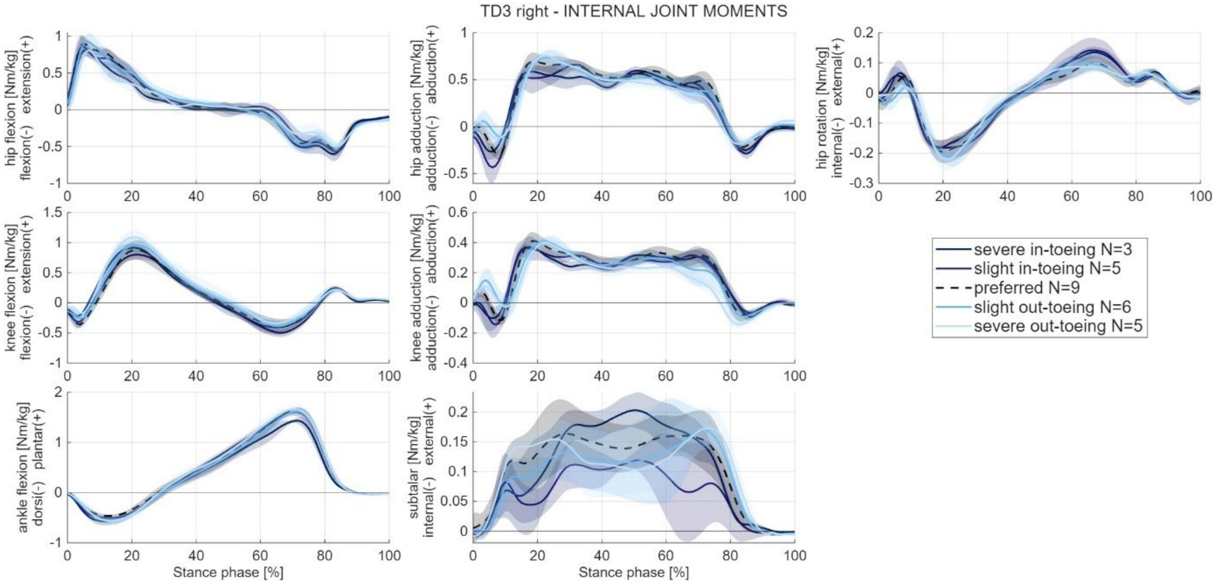

Figure S42: Mean and standard deviation (shaded) of internal joint moments quantified with OpenSim's Inverse Dynamics

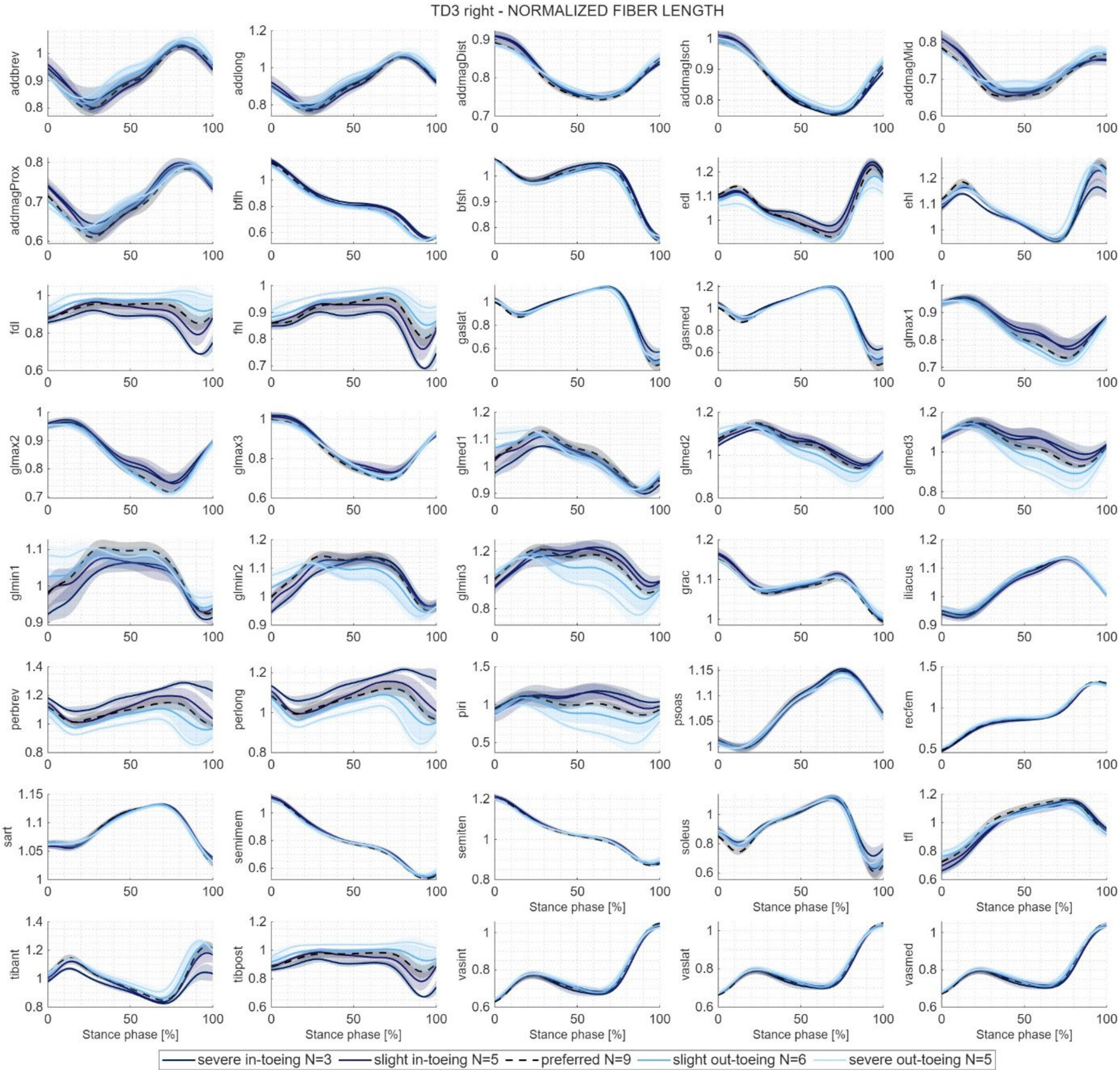

Figure S43: Mean and standard deviation (shaded) of normalized muscle fiber length quantified with OpenSim's Muscle Analysis

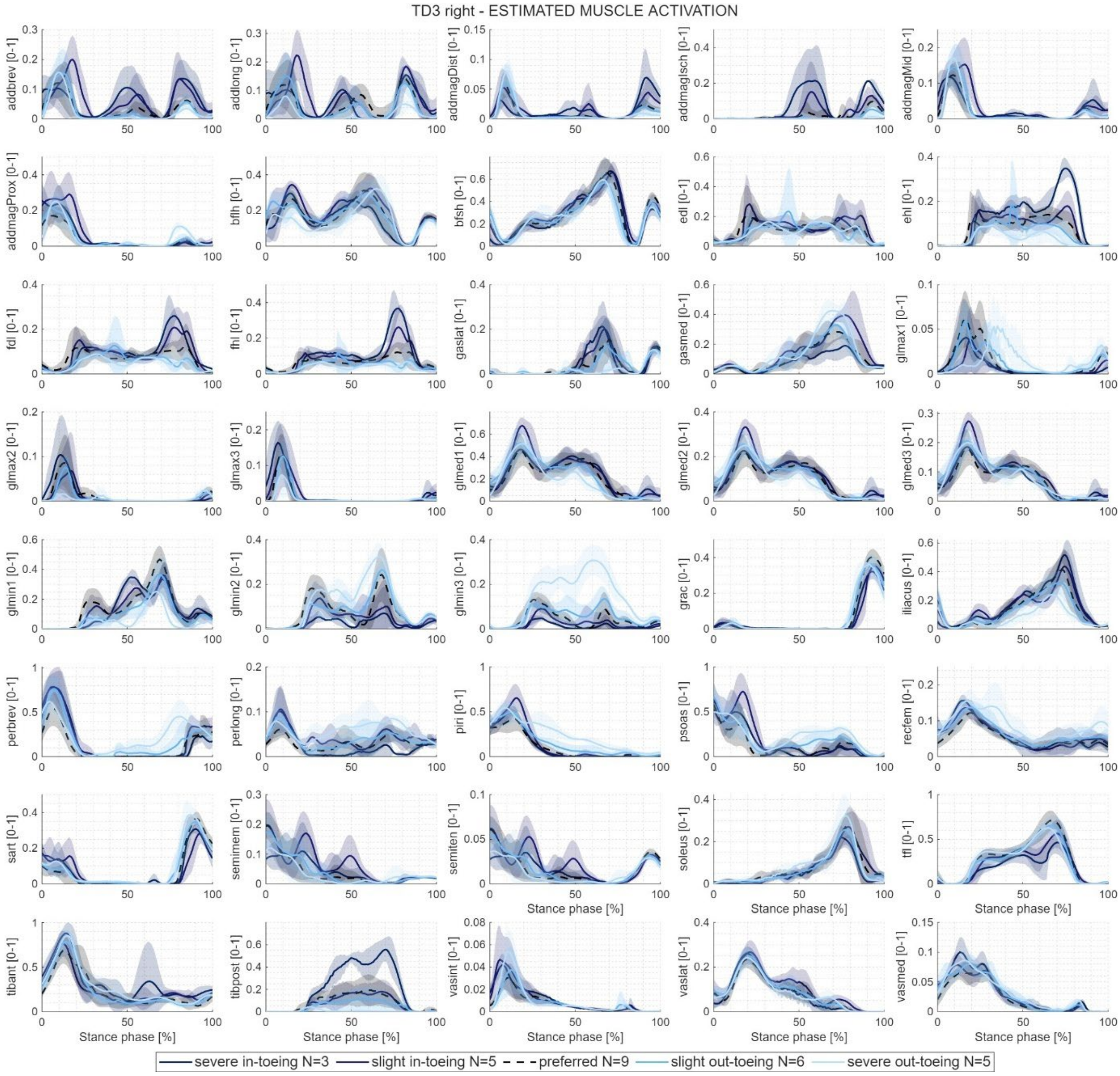

Figure S44: Mean and standard deviation (shaded) of estimated muscle activations obtained by EMG-informed simulations performed with the MuscleRedundancySolver.

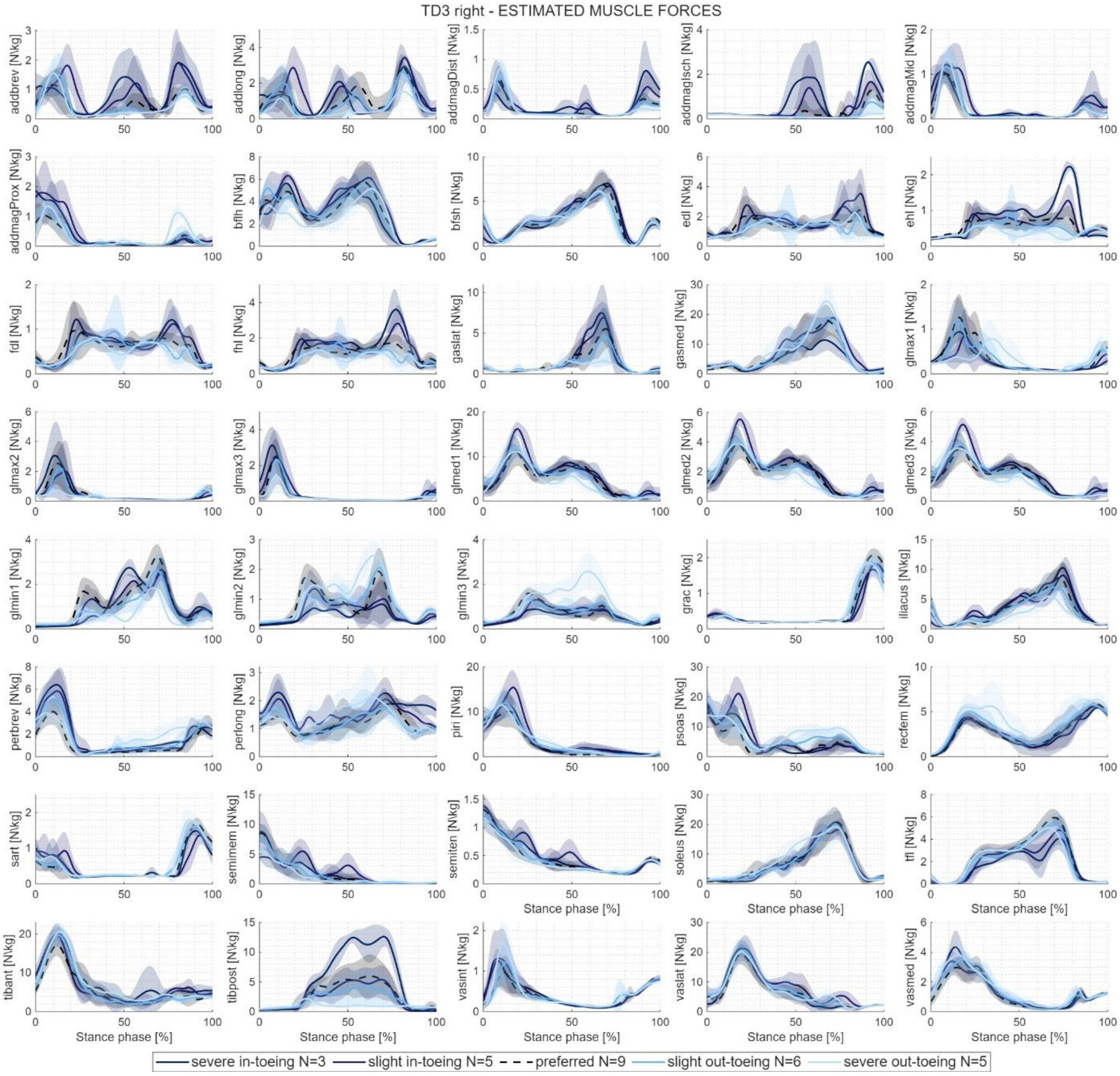

Figure S45: Mean and standard deviation (shaded) of estimated muscle forces obtained by EMG-informed simulations performed with the MuscleRedundancySolver.

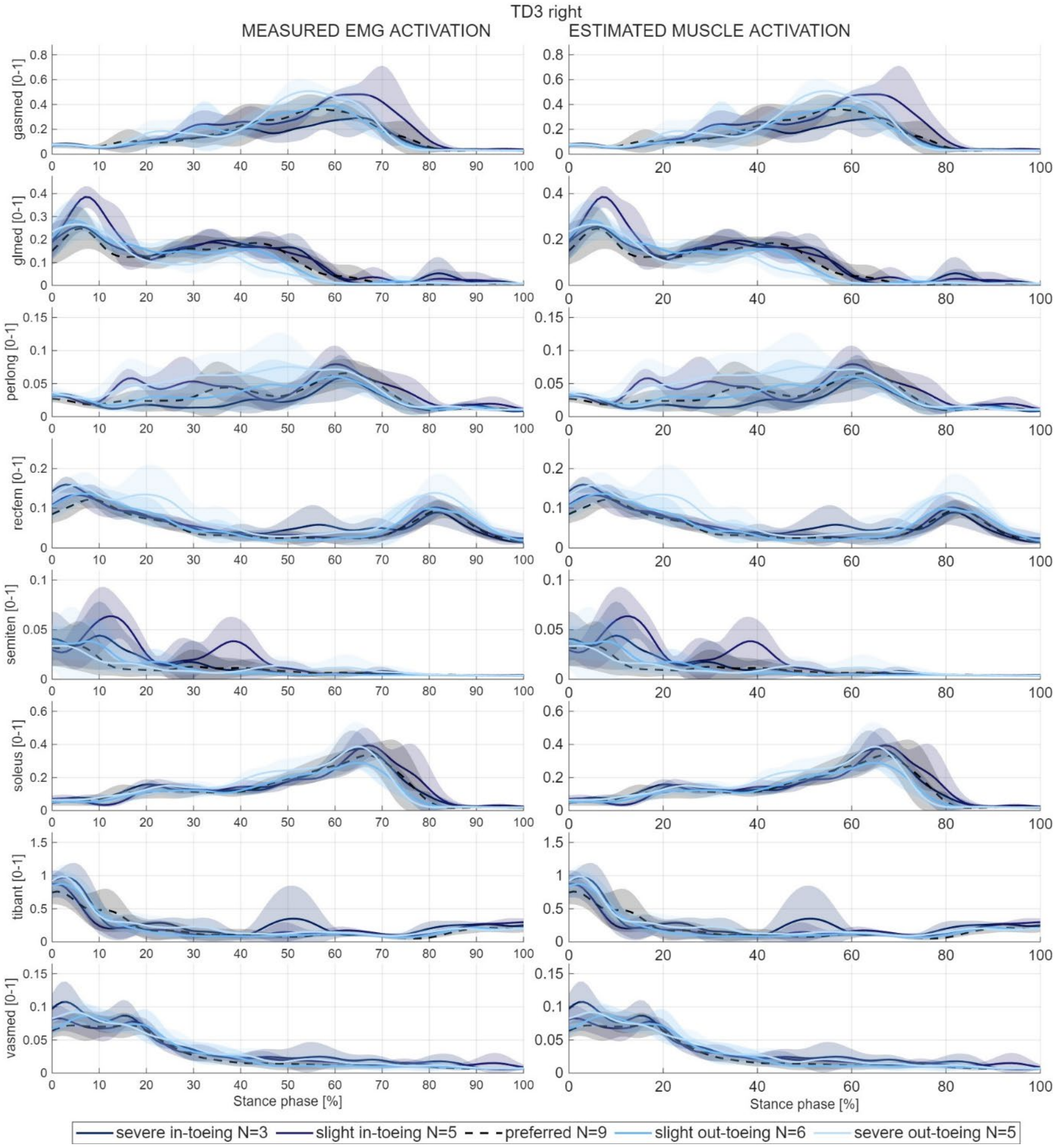

Figure S46: Mean and standard deviation (shaded) of measured muscle activation with electromyography (EMG) compared with the estimated muscle activation of the corresponding muscle in musculoskeletal simulations. Amplitude of EMG was scaled based on each muscle's maximum activation obtained from Static Optimization for the representative preferred gait trial.

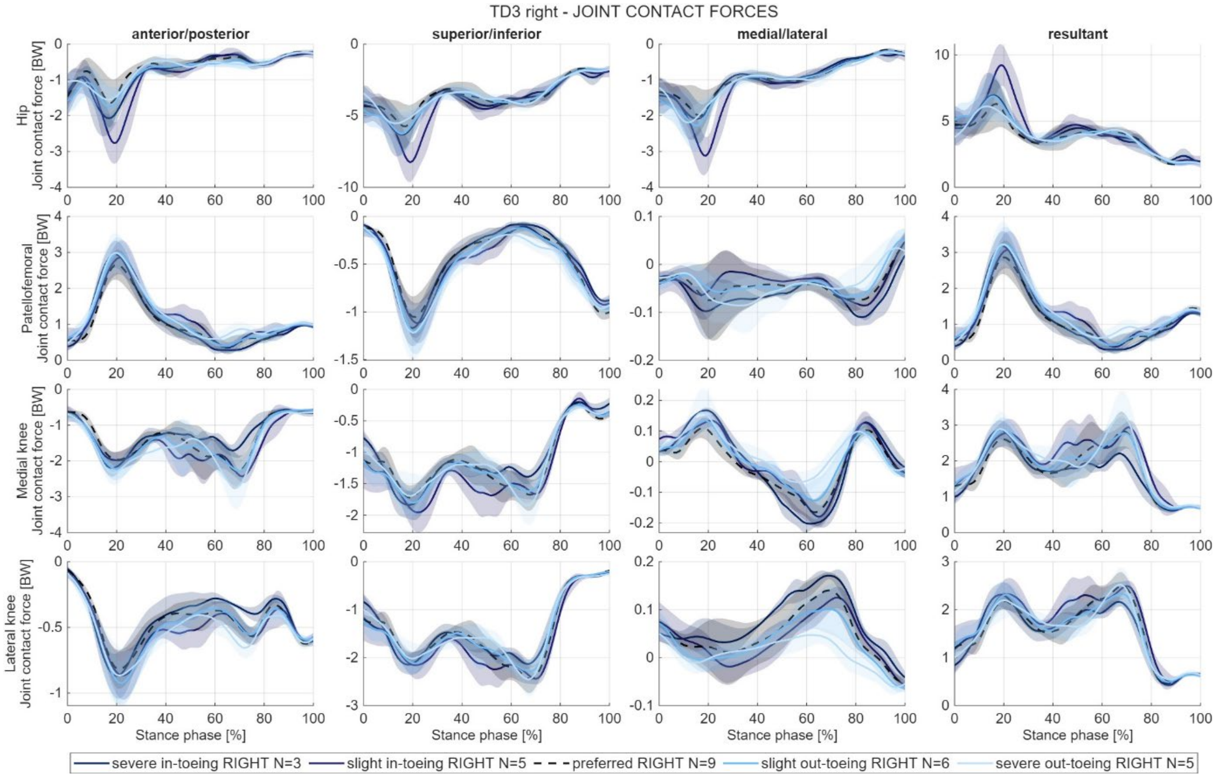

Figure S47: Mean and standard deviation (shaded) of hip, patellofemoral, medial and lateral knee joint contact forces obtained with OpenSim's Joint Reaction Analysis.

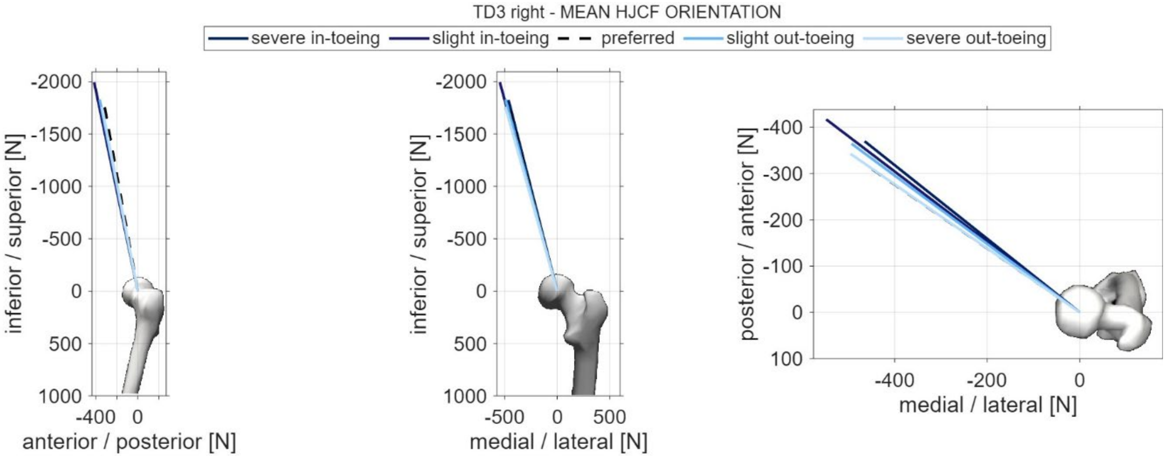

Figure S48:Visualization of the orientation vector of the mean resultant hip joint contact force (HJCF)
